# Multiorgan MRI findings after hospitalisation with COVID-19 in the UK (C-MORE): a prospective, multicentre, observational cohort study

**DOI:** 10.1016/S2213-2600(23)00262-X

**Published:** 2023-09-22

**Authors:** C-MORE/PHOSP-COVID Collaborative group, Core Management Group, C E Brightling, R A Evans, L V Wain, J D Chalmers, V C Harris, L P Ho, A Horsley, M Marks, K Poinasamy, B Raman, A Shikotra, A Singapuri, PHOSP-COVID Study Central Coordinating Team, C E Brightling, R A Evans, L V Wain, R Dowling, C Edwardson, O Elneima, S Finney, N J Greening, B Hargadon, V C Harris, L Houchen--Wolloff, O C Leavy, H J C McAuley, C Overton, T Plekhanova, R M Saunders, M Sereno, A Singapuri, A Shikotra, C Taylor, S Terry, C Tong, B Zhao, Steering Committee, D Lomas, E Sapey, C Berry, C E Bolton, N Brunskill, E R Chilvers, R Djukanovic, Y Ellis, D Forton, N French, J George, N A Hanley, N Hart, L McGarvey, N Maskell, H McShane, M Parkes, D Peckham, P Pfeffer, A Sayer, A Sheikh, A A R Thompson, N Williams, core management group representation, Executive Board, C E Brightling, Bioresource, W Greenhalf, M G Semple, M Ashworth, H E Hardwick, L Lavelle-Langham, W Reynolds, M Sereno, R M Saunders, A Singapuri, V Shaw, A Shikotra, B Venson, L V Wain, Data Hub, A B Docherty, E M Harrison, A Sheikh, K Baillie, C E Brightling, L Daines, R Free, R A Evans, S Kerr, O C Leavy, N I Lone, H J C McAuley, R Pius, J Quint, M Richardson, M Sereno, M Thorpe, L V Wain, Imaging Alliance, M Halling-Brown, F Gleeson, J Jacob, S Neubauer, B Raman, S Siddiqui, J M Wild, S Aslani, P Jezzard, H Lamlum, W Lilaonitkul, E Tunnicliffe, J Willoughby, C-MORE Investigators (Part of Imaging Alliance), C-MORE, S Neubauer, B Raman, M Beggs, M P Cassar, A Chiribiri, E Cox, D J Cuthbertson, V M Ferreira, L Finnigan, S Francis, M Halling-Brown, G J Kemp, H Lamlum, E Lukaschuk, C Manisty, G P McCann, K McGlynn, R Menke, C A Miller, A J Moss, C Nikolaidou, C O’Brien, D P O’Regan, S Piechnik, S Plein, I Propescu, A A Samat, L Saunders, R Steeds, T Treibel, E M Tunnicliffe, J Weir McCall, J M Wild, C Xie, G Ogbole, Z B Sanders, M Webster, S Smith, Miller K, C McCracken, T E Nichols, P Jezzard, Omics, L V Wain, J K Baillie, H Baxendale, C E Brightling, M Brown, J D Chalmers, R A Evans, B Gooptu, W Greenhalf, H E Hardwick, R G Jenkins, D Jones, I Koychev, C Langenberg, A Lawrie, P L Molyneaux, A Shikotra, J Pearl, M Ralser, N Sattar, R M Saunders, J T Scott, T Shaw, D Thomas, D Wilkinson, Working Groups, Airways, L G Heaney, A De Soyza, D Adeloye, C E Brightling, J S Brown, J Busby, J D Chalmers, C Echevarria, L Daines, O Elneima, RA Evans, J Hurst, P Novotny, P Pfeffer, K Poinasamy, J Quint, I Rudan, E Sapey, M Shankar-Hari, A Sheikh, S Siddiqui, S Walker, B Zheng, Brain, J R Geddes, M Hotopf, K Abel, R Ahmed, L Allan, C Armour, D Baguley, D Baldwin, C Ballard, K Bhui, G Breen, M Broome, T Brugha, E Bullmore, D Burn, F Callard, J Cavanagh, T Chalder, D Clark, A David, B Deakin, H Dobson, B Elliott, J Evans, R Francis, E Guthrie, P Harrison, M Henderson, A Hosseini, N Huneke, M Husain, T Jackson, I Jones, T Kabir, P Kitterick, A Korszun, I Koychev, J Kwan, A Lingford-Hughes, P Mansoori, H McAllister-Williams, K McIvor, L Milligan, R Morriss, E Mukaetova-Ladinska, K Munro, A Nevado-Holgado, T Nicholson, S Paddick, C Pariante, J Pimm, K Saunders, M Sharpe, G Simons, R Upthegrove, S Wessely, Cardiac, G P McCann, S Amoils, C Antoniades, A Banerjee, R Bell, A Bularga, C Berry, P Chowienczyk, J P Greenwood, A D Hughes, K Khunti, L Kingham, C Lawson, K Mangion, N L Mills, A J Moss, S Neubauer, B Raman, A N Sattar, C L Sudlow, M Toshner, Immunology, P J M Openshaw, D Altmann, J K Baillie, R Batterham, H Baxendale, N Bishop, C E Brightling, P C Calder, R A Evans, J L Heeney, T Hussell, P Klenerman, J M Lord, P Moss, S L Rowland-Jones, W Schwaeble, M G Semple, R S Thwaites, L Turtle, L V Wain, S Walmsley, D Wraith, Intensive Care, M J Rowland, A Rostron, J K Baillie, B Connolly, A B Docherty, N I Lone, D F McAuley, D Parekh, A Rostron, J Simpson, C Summers, Lung Fibrosis, R G Jenkins, J Porter, R J Allen, R Aul, J K Baillie, S Barratt, P Beirne, J Blaikley, R C Chambers, N Chaudhuri, C Coleman, E Denneny, L Fabbri, P M George, M Gibbons, F Gleeson, B Gooptu, B Guillen Guio, I Hall, N A Hanley, L P Ho, E Hufton, J Jacob, I Jarrold, G Jenkins, S Johnson, M G Jones, S Jones, F Khan, P Mehta, J Mitchell, P L Molyneaux, J E Pearl, K Piper Hanley, K Poinasamy, J Quint, D Parekh, P Rivera-Ortega, L C Saunders, M G Semple, J Simpson, D Smith, M Spears, L G Spencer, S Stanel, I Stewart, A A R Thompson, D Thickett, R Thwaites, L V Wain, S Walker, S Walsh, J M Wild, D G Wootton, L Wright, Metabolic, S Heller, M J Davies, H Atkins, S Bain, J Dennis, K Ismail, D Johnston, P Kar, K Khunti, C Langenberg, P McArdle, A McGovern, T Peto, J Petrie, E Robertson, N Sattar, K Shah, J Valabhji, B Young, Pulmonary and Systematic Vasculature, L S Howard, Mark Toshner, C Berry, P Chowienczyk, D Lasserson, A Lawrie, O C Leavy, J Mitchell, J Newman, L Price, J Quint, A Reddy, J Rossdale, N Sattar, C Sudlow, A A R Thompson, J M Wild, M Wilkins, Rehabilitation, Sarcopenia and Fatigue, S J Singh, W D-C Man, J M Lord, N J Greening, T Chalder, J T Scott, N Armstrong, E Baldry, M Baldwin, N Basu, M Beadsworth, L Bishop, C E Bolton, A Briggs, M Buch, G Carson, J Cavanagh, H Chinoy, E Daynes, S Defres, R A Evans, P Greenhaff, S Greenwood, M Harvie, M Husain, S MacDonald, A McArdle, H J C McAuley, A McMahon, M McNarry, G Mills, C Nolan, K O’Donnell, D Parekh, Pimm, J Sargent, L Sigfrid, M Steiner, D Stensel, A L Tan, J Whitney, D Wilkinson, D Wilson, M Witham, D G Wootton, T Yates, Renal, D Thomas, N Brunskill, S Francis, S Greenwood, C Laing, K Bramham, P Chowdhury, A Frankel, L Lightstone, S McAdoo, K McCafferty, M Ostermann, N Selby, C Sharpe, M Willicombe, Local Clinical Centre PHOSP-COVID trial staff, Airedale NHS Foundation Trust, A Shaw, L Armstrong, B Hairsine, H Henson, C Kurasz, L Shenton, Aneurin Bevan University Health Board, S Fairbairn, A Dell, N Hawkings, J Haworth, M Hoare, A Lucey, V Lewis, G Mallison, H Nassa, C Pennington, A Price, C Price, A Storrie, G Willis, S Young, Barts Health NHS Trust & Queen Mary University of London, P Pfeffer, K Chong-James, C David, W Y James, A Martineau, O Zongo, Charlotte Manisty, T Treibel, Barnsley Hospital NHS Foundation Trust, A Sanderson, Belfast Health and Social Care Trust & Queen’s University Belfast, L G Heaney, C Armour, V Brown, T Craig, S Drain, B King, N Magee, D McAulay, E Major, L McGarvey, J McGinness, R Stone, Betsi Cadwaladr University Health Board, A Haggar, A Bolger, F Davies, J Lewis, A Lloyd, R Manley, E McIvor, D Menzies, K Roberts, W Saxon, D Southern, C Subbe, V Whitehead, Borders General Hospital, NHS Borders, H El-Taweel, J Dawson, L Robinson, Bradford Teaching Hospitals NHS Foundation Trust, D Saralaya, L Brear, K Regan, K Storton, Cambridge University Hospitals NHS Foundation Trust, NIHR Cambridge Clinical Research Facility & University of Cambridge, J Fuld, A Bermperi, I Cruz, K Dempsey, A Elmer, H Jones, S Jose, S Marciniak, M Parkes, C Ribeiro, J Taylor, M Toshner, L Watson, J Worsley, J Weir McCall, Cardiff and Vale University Health Board, R Sabit, L Broad, A Buttress, T Evans, M Haynes, L Jones, L Knibbs, A McQueen, C Oliver, K Paradowski, J Williams, Chesterfield Royal Hospital NHS Trust, E Harris, C Sampson, Cwm Taf Morgannwg University Health Board, C Lynch, E Davies, C Evenden, A Hancock, K Hancock, M Rees, L Roche, N Stroud, T Thomas-Woods, East Cheshire NHS Trust, M Babores, J Bradley-Potts, M Holland, N Keenan, S Shashaa, H Wassall, East Kent Hospitals University NHS Foundation Trust, E Beranova, H Weston, T Cosier, L Austin, J Deery, T Hazelton, C Price, H Ramos, R Solly, S Turney, Gateshead NHS Trust, L Pearce, W McCormack, S Pugmire, W Stoker, A Wilson, Guy’s and St Thomas’ NHS Foundation Trust, N Hart, LA Aguilar Jimenez, G Arbane, S Betts, K Bisnauthsing, A Dewar, P Chowdhury, A Dewar, G Kaltsakas, H Kerslake, MM Magtoto, P Marino, LM Martinez, M Ostermann, J Rossdale, TS Solano, E Wynn, Hampshire Hospitals NHS Foundation Trust, N Williams, W Storrar, M Alvarez Corral, A Arias, E Bevan, D Griffin, J Martin, J Owen, S Payne, A Prabhu, A Reed, C Wrey Brown, Harrogate and District NHD Foundation Trust, C Lawson, T Burdett, J Featherstone, A Layton, C Mills, L Stephenson, Hull University Teaching Hospitals NHS Trust & University of Hull, N Easom, P Atkin, K Brindle, M G Crooks, K Drury, R Flockton, L Holdsworth, A Richards, D L Sykes, S Thackray-Nocera, C Wright, Hywel Dda University Health Board, K E Lewis, A Mohamed, G Ross, S Coetzee, K Davies, R Hughes, R Loosley, L O’Brien, Z Omar, H McGuinness, E Perkins, J Phipps, A Taylor, H Tench, R Wolf-Roberts, Imperial College Healthcare NHS Trust & Imperial College London, L S Howard, O Kon, D C Thomas, S Anifowose, L Burden, E Calvelo, B Card, C Carr, E R Chilvers, D Copeland, P Cullinan, P Daly, L Evison, T Fayzan, H Gordon, S Haq, R G Jenkins, C King, K March, M Mariveles, L McLeavey, N Mohamed, S Moriera, U Munawar, J Nunag, U Nwanguma, L Orriss-Dib, A Ross, M Roy, E Russell, K Samuel, J Schronce, N Simpson, L Tarusan, C Wood, N Yasmin, D P O’Regan, Kettering General Hospital NHS Trust, R Reddy, A-M Guerdette, M Hewitt, K Warwick, S White, King’s College Hospital NHS Foundation Trust & Kings College London, A M Shah, C J Jolley, O Adeyemi, R Adrego, H Assefa-Kebede, J Breeze, M Brown, S Byrne, T Chalder, P Dulawan, N Hart, A Hayday, A Hoare, A Knighton, M Malim, S Patale, I Peralta, N Powell, A Ramos, K Shevket, F Speranza, A Te, Leeds Teaching Hospitals & University of Leeds, P Beirne, A Ashworth, J Clarke, C Coupland, M Dalton, E Wade, C Favager, J Greenwood, J Glossop, L Hall, T Hardy, A Humphries, J Murira, D Peckham, S Plein, J Rangeley, G Saalmink, A L Tan, B Whittam, N Window, J Woods, S Plein, Lewisham & Greenwich NHS Trust, G Coakley, Liverpool University Hospitals NHS Foundation Trust & University of Liverpool, D G Wootton, L Turtle, L Allerton, AM All, M Beadsworth, A Berridge, J Brown, S Cooper, A Cross, S Defres, S L Dobson, J Earley, N French, W Greenhalf, H E Hardwick, K Hainey, J Hawkes, V Highett, S Kaprowska, AL Key, L Lavelle-Langham, N Lewis-Burke, G Madzamba, F Malein, S Marsh, C Mears, L Melling, M J Noonan, L Poll, J Pratt, E Richardson, A Rowe, M G Semple, V Shaw, K A Tripp, L O Wajero, S A Williams-Howard, J Wyles, G J Kemp, D J Cuthbertson, London North West University Healthcare NHS Trust, S N Diwanji, P Papineni, S Gurram, S Quaid, G F Tiongson, E Watson, Manchester University NHS Foundation Trust & University of Manchester, B Al-Sheklly, A Horsley, C Avram, J Blaikely, M Buch, N Choudhury, D Faluyi, T Felton, T Gorsuch, N A Hanley, T Hussell, Z Kausar, N Odell, R Osbourne, K Piper Hanley, K Radhakrishnan, S Stockdale, C A Miller, Newcastle upon Tyne Hospitals NHS Foundation Trust & University of Newcastle, A De Soyza, C Echevarria, A Ayoub, J Brown, G Burns, G Davies, H Fisher, C Francis, A Greenhalgh, P Hogarth, J Hughes, K Jiwa, G Jones, G MacGowan, D Price, A Sayer, J Simpson, H Tedd, S Thomas, S West, M Witham, S Wright, A Young, NHS Dumfries and Galloway, M J McMahon, P Neill, NHS Greater Glasgow and Clyde Health Board & University of Glasgow, D Anderson, H Bayes, C Berry, D Grieve, I B McInnes, N Basu, A Brown, A Dougherty, K Fallon, L Gilmour, K Mangion, A Morrow, K Scott, R Sykes, NHS Highland, E K Sage, F Barrett, A Donaldson, NHS Lanarkshire, M Patel, D Bell, A Brown, M Brown, R Hamil, K Leitch, L Macliver, J Quigley, A Smith, B Welsh, NHS Lothian & University of Edinburgh, G Choudhury, J K Baillie, S Clohisey, A Deans, A B Docherty, J Furniss, E M Harrison, S Kelly, N I Lone, A Sheikh, NHS Tayside & University of Dundee, J D Chalmers, D Connell, A Elliott, C Deas, J George, S Mohammed, J Rowland, A R Solstice, D Sutherland, C J Tee, North Bristol NHS Trust & University of Bristol, N Maskell, D Arnold, S Barrett, H Adamali, A Dipper, S Dunn, A Morley, L Morrison, L Stadon, S Waterson, H Welch, North Middlesex Hospital NHS Trust, B Jayaraman, T Light, Nottingham University Hospitals NHS Trust & University of Nottingham, C E Bolton, P Almeida, J Bonnington, M Chrystal, C Dupont, P Greenhaff, A Gupta, L Howard, W Jang, S Linford, L Matthews, R Needham, A Nikolaidis, S Prosper, K Shaw, A K Thomas, S Francis, Oxford University Hospitals NHS Foundation Trust & University of Oxford, L P Ho, N M Rahman, M Ainsworth, A Alamoudi, A Bates, A Bloss, A Burns, P Carter, J Chen, F Conneh, T Dong, R I Evans, E Fraser, X Fu, J R Geddes, F Gleeson, P Harrison, M Havinden-Williams, P Jezzard, N Kanellakis, I Koychev, P Kurupati, X Li, H McShane, C Megson, K Motohashi, S Neubauer, D Nicoll, G Ogg, E Pacpaco, M Pavlides, Y Peng, N Petousi, N Rahman, B Raman, M J Rowland, K Saunders, M Sharpe, N Talbot, E Tunnicliffe, Royal Brompton and Harefield Clinical Group, Guy’s and St Thomas’ NHS Foundation Trust, W D-C Man, B Patel, R E Barker, D Cristiano, N Dormand, M Gummadi, S Kon, K Liyanage, C M Nolan, S Patel, O Polgar, P Shah, S J Singh, J A Walsh, Royal Free London NHS Foundation Trust, J Hurst, H Jarvis, S Mandal, S Ahmad, S Brill, L Lim, D Matila, O Olaosebikan, C Singh, Royal Papworth Hospital NHS Foundation Trust, M Toshner, H Baxendale, L Garner, C Johnson, J Mackie, A Michael, J Pack, K Paques, H Parfrey, J Parmar, Salford Royal NHS Foundation Trust, N Diar Bakerly, P Dark, D Evans, E Hardy, A Harvey, D Holgate, S Knight, N Mairs, N Majeed, L McMorrow, J Oxton, J Pendlebury, C Summersgill, R Ugwuoke, S Whittaker, Salisbury NHS Foundation Trust, W Matimba-Mupaya, S Strong-Sheldrake, Sheffield Teaching NHS Foundation Trust & University of Sheffield, S L Rowland-Jones, A A R Thompson, J Bagshaw, M Begum, K Birchall, R Butcher, H Carborn, F Chan, K Chapman, Y Cheng, L Chetham, C Clark, Z Coburn, J Cole, M Dixon, A Fairman, J Finnigan, H Foot, D Foote, A Ford, R Gregory, K Harrington, L Haslam, L Hesselden, J Hockridge, A Holbourn, B Holroyd-Hind, L Holt, A Howell, E Hurditch, F Ilyas, C Jarman, A Lawrie, E Lee, J-H Lee, R Lenagh, A Lye, I Macharia, M Marshall, A Mbuyisa, J McNeill, S Megson, J Meiring, L Milner, S Misra, H Newell, T Newman, C Norman, L Nwafor, D Pattenadk, M Plowright, J Porter, P Ravencroft, C Roddis, J Rodger, P Saunders, J Sidebottom, J Smith, L Smith, N Steele, G Stephens, R Stimpson, B Thamu, N Tinker, K Turner, H Turton, P Wade, S Walker, J Watson, I Wilson, A Zawia, J M Wild, St George’s University Hospitals NHS Foundation Trust, R Aul, M Ali, A Dunleavy, D Forton, N Msimanga, M Mencias, T Samakomva, S Siddique, J Teixeira, V Tavoukjian, Sherwood Forest Hospitals NHS Foundation Trust, J Hutchinson, L Allsop, K Bennett, P Buckley, M Flynn, M Gill, C Goodwin, M Greatorex, H Gregory, C Heeley, L Holloway, M Holmes, J Kirk, W Lovegrove, TA Sewell, S Shelton, D Sissons, K Slack, S Smith, D Sowter, S Turner, V Whitworth, I Wynter, Shropshire Community Health NHS Trust, L Warburton, S Painter, J Tomlinson, Somerset NHS Foundation Trust, C Vickers, T Wainwright, D Redwood, J Tilley, S Palmer, Swansea Bay University Health Board, G A Davies, L Connor, A Cook, T Rees, F Thaivalappil, C Thomas, Tameside and Glossop Integrated Care NHS Foundation, A Butt, M Coulding, H Jones, S Kilroy, J McCormick, J McIntosh, H Savill, V Turner, J Vere, The Great Western Hospital Foundation Trust, E Fraile, J Ugoji, The Hillingdon Hospitals NHS Foundation Trust, S S Kon, H Lota, G Landers, M Nasseri, S Portukhay, The Rotherham NHS Foundation Trust, A Hormis, A Daniels, J Ingham, L Zeidan, United Lincolnshire Hospitals NHS Trust, M Chablani, L Osborne, University College London Hospital & University College London, M Marks, J S Brown, N Ahwireng, B Bang, D Basire, R C Chambers, A Checkley, R Evans, M Heightman, T Hillman, J Hurst, J Jacob, S Janes, R Jastrub, M Lipman, S Logan, D Lomas, M Merida Morillas, H Plant, J C Porter, K Roy, E Wall, T Treibel, University Hospital Birmingham NHS Foundation Trust & University of Birmingham, D Parekh, N Ahmad Haider, C Atkin, R Baggott, M Bates, A Botkai, A Casey, B Cooper, J Dasgin, K Draxlbauer, N Gautam, J Hazeldine, T Hiwot, S Holden, K Isaacs, T Jackson, S Johnson, V Kamwa, D Lewis, J M Lord, S Madathil, C McGhee, K Mcgee, A Neal, A Newton Cox, J Nyaboko, D Parekh, Z Peterkin, H Qureshi, L Ratcliffe, E Sapey, J Short, T Soulsby, J Stockley, Z Suleiman, T Thompson, M Ventura, S Walder, C Welch, D Wilson, S Yasmin, K P Yip, R Steeds, University Hospitals of Derby and Burton, P Beckett, C Dickens, U Nanda, University Hospitals of Leicester NHS Trust & University of Leicester, C E Brightling, R A Evans, M Aljaroof, N Armstrong, H Arnold, H Aung, M Bakali, M Bakau, M Baldwin, M Bingham, M Bourne, C Bourne, N Brunskill, P Cairns, L Carr, A Charalambou, C Christie, M J Davies, S Diver, S Edwards, C Edwardson, O Elneima, H Evans, J Finch, S Glover, N Goodman, B Gootpu, N J Greening, K Hadley, P Haldar, B Hargadon, V C Harris, L Houchen-Wolloff, W Ibrahim, L Ingram, K Khunti, A Lea, D Lee, G P McCann, H J C McAuley, P McCourt, T Mcnally, G Mills, A Moss, W Monteiro, M Pareek, S Parker, A Rowland, A Prickett, I N Qureshi, R Russell, M Sereno, A Shikotra, S Siddiqui, A Singapuri, S J Singh, J Skeemer, M Soares, E Stringer, T Thornton, M Tobin, L V Wain, T J C Ward, F Woodhead, T Yates, A Yousuf, University Hospital Southampton NHS Foundation Trust & University of Southampton, M G Jones, C Childs, R Djukanovic, S Fletcher, M Harvey, E Marouzet, B Marshall, R Samuel, T Sass, T Wallis, H Wheeler, Whittington Health NHS, R Dharmagunawardena, E Bright, P Crisp, M Stern, Wirral University Teaching Hospital, A Wight, L Bailey, A Reddington, Wrightington Wigan and Leigh NHS trust, A Ashish, J Cooper, E Robinson, Yeovil District Hospital NHS Foundation Trust, A Broadley, York & Scarborough NHS Foundation Trust, K Howard, L Barman, C Brookes, K Elliott, L Griffiths, Z Guy, D Ionita, H Redfearn, C Sarginson, A Turnbull, Health and Care Research Wales, Y Ellis, London School of Hygiene & Tropical Medicine (LSHTM), M Marks, A Briggs, NIHR Office for Clinical Research Infrastructure, K Holmes, Patient Public Involvement Leads, Asthma UK and British Lung Foundation Partnership, K Poinasamy, S Walker, Royal Surrey NHS Foundation Trust, M Halling-Brown, South London and Maudsley NHS Foundation Trust & Kings College London, G Breen, M Hotopf, Swansea University & Swansea Welsh Network, K Lewis, N Williams

## Abstract

Introduction The multiorgan impact of moderate to severe coronavirus infections in the post-acute phase is still poorly understood. We aimed to evaluate the excess burden of multiorgan abnormalities after hospitalisation with COVID-19, evaluate their determinants, and explore associations with patient-related outcome measures.

**Methods:**

In a prospective, UK-wide, multicentre MRI follow-up study (C-MORE), adults (aged ≥18 years) discharged from hospital following COVID-19 who were included in Tier 2 of the Post-hospitalisation COVID-19 study (PHOSP-COVID) and contemporary controls with no evidence of previous COVID-19 (SARS-CoV-2 nucleocapsid antibody negative) underwent multiorgan MRI (lungs, heart, brain, liver, and kidneys) with quantitative and qualitative assessment of images and clinical adjudication when relevant. Individuals with end-stage renal failure or contraindications to MRI were excluded. Participants also underwent detailed recording of symptoms, and physiological and biochemical tests. The primary outcome was the excess burden of multiorgan abnormalities (two or more organs) relative to controls, with further adjustments for potential confounders. The C-MORE study is ongoing and is registered with ClinicalTrials.gov, NCT04510025.

**Findings:**

Of 2710 participants in Tier 2 of PHOSP-COVID, 531 were recruited across 13 UK-wide C-MORE sites. After exclusions, 259 C-MORE patients (mean age 57 years [SD 12]; 158 [61%] male and 101 [39%] female) who were discharged from hospital with PCR-confirmed or clinically diagnosed COVID-19 between March 1, 2020, and Nov 1, 2021, and 52 non-COVID-19 controls from the community (mean age 49 years [SD 14]; 30 [58%] male and 22 [42%] female) were included in the analysis. Patients were assessed at a median of 5·0 months (IQR 4·2–6·3) after hospital discharge. Compared with non-COVID-19 controls, patients were older, living with more obesity, and had more comorbidities. Multiorgan abnormalities on MRI were more frequent in patients than in controls (157 [61%] of 259 *vs* 14 [27%] of 52; p<0·0001) and independently associated with COVID-19 status (odds ratio [OR] 2·9 [95% CI 1·5–5·8]; p_adjusted_=0·0023) after adjusting for relevant confounders. Compared with controls, patients were more likely to have MRI evidence of lung abnormalities (p=0·0001; parenchymal abnormalities), brain abnormalities (p<0·0001; more white matter hyperintensities and regional brain volume reduction), and kidney abnormalities (p=0·014; lower medullary T1 and loss of corticomedullary differentiation), whereas cardiac and liver MRI abnormalities were similar between patients and controls. Patients with multiorgan abnormalities were older (difference in mean age 7 years [95% CI 4–10]; mean age of 59·8 years [SD 11·7] with multiorgan abnormalities *vs* mean age of 52·8 years [11·9] without multiorgan abnormalities; p<0·0001), more likely to have three or more comorbidities (OR 2·47 [1·32–4·82]; p_adjusted_=0·0059), and more likely to have a more severe acute infection (acute CRP >5mg/L, OR 3·55 [1·23–11·88]; p_adjusted_=0·025) than those without multiorgan abnormalities. Presence of lung MRI abnormalities was associated with a two-fold higher risk of chest tightness, and multiorgan MRI abnormalities were associated with severe and very severe persistent physical and mental health impairment (PHOSP-COVID symptom clusters) after hospitalisation.

**Interpretation:**

After hospitalisation for COVID-19, people are at risk of multiorgan abnormalities in the medium term. Our findings emphasise the need for proactive multidisciplinary care pathways, with the potential for imaging to guide surveillance frequency and therapeutic stratification.

## Introduction

Long-standing multiorgan impairment following SARS-CoV-2 infection has been a major concern for individuals recovering from severe disease (eg, after hospitalisation^1,2^) and is thought to be caused by a multitude of factors, including direct viral cytotoxicity,^3^ chronic inflammation,^4^ ischaemic injury,^5^ acute reactivation of other viruses,^6^ metabolic derangements, and acute treatment effects (especially invasive ventilation). Numerous reports of delayed organ complications such as myocarditis, stroke, and pulmonary emboli have led to speculations that the multiorgan dysfunction caused by COVID-19 might be responsible for impaired recovery and ongoing symptoms in individuals, a condition referred to as long COVID. However, what is currently unknown is the precise burden of persistent multiorgan impairment after hospitalisation with COVID-19 and how this impairment might affect patient recovery and symptoms.

MRI has several strengths as a safe and reproducible tool for the assessment of post-COVID-19 organ manifestations.^7^ In a previous pilot study, we applied multiorgan MRI^8^ to delineate the extent of organ injury after hospital admission in survivors of COVID-19, and noted moderate associations between inflammatory markers and abnormal tissue characteristics, implying a dominant role of inflammation in persistent multiorgan dysfunction. Others have also observed persistent deviations in haemostatic pathways after infection.^9^ To further evaluate the burden of multiorgan dysfunction and its effects on patient recovery following moderate to severe SARS-CoV-2 infection, we embarked on a prospective, UK-wide, multiorgan, multicentre MRI follow-up study of post-hospitalised patients with COVID-19 called the Capturing Multiorgan Effects of COVID-19 (C-MORE) study.

C-MORE was developed to (1) characterise the excess prevalence of multiorgan abnormalities among survivors of COVID-19 relative to SARS-CoV-2-uninfected controls; (2) provide mechanistic insights into the source of multiorgan dysfunction; and (3) evaluate the associations of multiorgan MRI abnormalities with patient-reported outcome measures after COVID-19. Here we present the results of an interim analysis in our study.

## Methods

### Study design and participants

This prospective, observational, multicentre cohort study (C-MORE) was nested within a nationally prioritised COVID-19 follow-up programme called the Post-hospitalisation COVID-19 study (PHOSP-COVID).^10^ The C-MORE study enrolled patients admitted to hospital with either PCR-confirmed or clinically diagnosed COVID-19 between March 1, 2020, and Nov 1, 2021, and who gave written informed consent to participate in the PHOSP-COVID study (Leeds West Research Ethics Committee [20/YH/0225]).^10,11^ As part of Tier 2 of PHOSP-COVID,^10^ prospective clinical evaluation (eg, blood sampling, questionnaires, and lung function test) took place within 4 weeks of the MRI in a minimum of 500 consenting patients. Individuals who were asymptomatic for previous or recent COVID-19, had not been hospitalised over the past year, and had a negative SARS-CoV-2 PCR and nucleocapsid antibody test were invited to serve as controls, by word of mouth and through poster advertisements. Control participants had to be aged 18 years or older, without any MRI contraindications, and were prospectively enrolled from the community in Oxford, UK. Controls also underwent routine blood tests, selected questionnaires, and lung function testing.

C-MORE was set up in 13 of 40 Tier 2 PHOSP-COVID sites, which also hosted a 3 Tesla MRI scanner (minimum requirement). The sites were in Oxford, London (four centres), Manchester, Sheffield, Leeds, Liverpool, Leicester, Cambridge, Nottingham, and Birmingham (further details are provided in the appendix, pp 12–13). We excluded individuals with end-stage renal failure (estimated glomerular filtration rate [eGFR] <30 mL/min per 1·73 m^2^) or contraindications to MRI (eg, claustrophobia, relevant metal implant, or implanted device like defibrillator or pacemaker). A full list of inclusion and exclusion criteria is provided in the appendix (p 12). Data about the sex of participants were derived from patient history and were self-reported, with participants allowed to identify as any sex; all participants in this study were either male or female.

C-MORE was registered with ClinicalTrials.gov, NCT04510025, and approved by North West – Preston Research Ethics Committee (20/NW/0235).

### Procedures

On the day of the study visit, participant demographics, clinical characteristics, area of residence, WHO clinical progression scale (when relevant), and comorbidities (measured with the Charlson Comorbidity Index) were assessed. All participants also had blood tests (indicating organ health) and pulmonary function tests undertaken, and patient-reported outcomes measured (appendix p 20).^10^ Pulmonary function tests included assessment of FEV_1_, forced vital capacity (FVC), and, in some centres, transfer factor of the lung for carbon monoxide (TLCO; only available for patients). Participant-reported outcome measures were assessed using a series of questionnaires: the Generalized Anxiety Disorder 7-item scale, the Patient Health Questionnaire-9, the Montreal Cognitive Assessment, Dyspnoea-12, the Functional Assessment of Chronic Illness Therapy Fatigue Scale, the EQ-5D-5L utility index (patients only), and a bespoke COVID-19 symptom questionnaire (patients only; appendix pp 10–11). Based on the data from these questionnaires, patients who had COVID-19 were assigned to one of four previously described PHOSP-COVID symptom clusters (appendix p 22).^11^

Within 4 weeks of the study visit, participants underwent an MRI during which their heart rate, temperature, oxygen saturation, and blood pressure were recorded. For MRI acquisition, lungs, heart, brain, liver, and kidney scans were acquired using a 3 Tesla MRI scanner; further details of acquisition and analyses are provided in the appendix (pp 12–13). Lung MRI included a T2-weighted scan and perfusion imaging to assess the extent of lung parenchymal abnormalities and perfusion impairment. Cardiac MRI included cine imaging to assess biventricular volumes and function, T1 and T2 mapping for assessment of inflammation, post-contrast T1 mapping for assessment of diffuse fibrosis, and late gadolinium enhancement (LGE) imaging for assessment of focal fibrosis. Brain MRI included T1-weighted and T2-weighted imaging to evaluate global and regional brain volumes and assess for inflammatory changes. Diffusion-weighted imaging and susceptibility-weighted imaging were qualitatively assessed for ischaemic and haemorrhagic injury. Liver MRI included T2* imaging to assess liver iron and liver T1 mapping to assess liver fibrosis and inflammation. A multi-echo gradient echo sequence was also acquired to assess liver fat via proton density fat fraction (PDFF). Renal MRI included T2-weighted anatomical imaging to assess renal volumes and T1 mapping to assess renal fibro-inflammation and corticomedullary differentiation.

For MRI analyses, quantitative and qualitative (specifically for lungs, heart, and brain scans) analyses were undertaken by organ-specific core laboratories, where the analysts were masked to all participant characteristics including symptoms and COVID-19 status. Lung scans were qualitatively assessed by two accredited MRI experts, with a third experienced radiologist adjudicating cases of disagreement. Both the presence or absence (ie, a binary score of 0 to indicate ≤5% of lung involvement, or 1 when lung involvement was >5%) and extent of parenchymal abnormalities (up to 25%, 26–50%, 51–75%, and 76–100%) were evaluated. Lung perfusion was semi-quantitatively assessed as described earlier, and global pulmonary blood flow, blood volume, and mean transit time were computed. Cardiac analyses were undertaken using cvi42 software (version 5.12; Circle Cardiovascular Imaging, Calgary, Canada).^12^ Qualitative readouts of LGE imaging were undertaken by three experienced cardiac MRI readers. Brain image processing was undertaken using an adapted version of the processing pipeline created for the UK Biobank brain imaging analysis. Qualitative assessments of brain images were undertaken by an experienced neuroradiologist. Quantitative analyses of liver metrics, including PDFF, liver iron, and iron-corrected T1 (Liver cT1) were also undertaken using well established methods.^13^ Renal volumes and cortical and medullary T1, markers of microstructural health, were computed for both kidneys. Further details on image analyses are provided in the appendix (pp 13–17). Patients were assessed for single-organ or multiorgan (involvement of two or more organs) abnormalities based on deviations in qualitative and quantitative MRI characteristics, as described in the appendix (p 18).

### Outcomes

The prespecified primary endpoint was the excess burden of multiorgan abnormalities on MRI among patients after hospitalisation for COVID-19 relative to non-COVID-19 controls at a median of 5–6 months after discharge. Secondary outcomes included the association of organ abnormality on MRI and persistent symptoms and impaired recovery in patients at 5–6 months after discharge and relative to controls. Exploratory outcomes included prevalence of MRI abnormalities at 2–3 months and 12–18 months after infection in patients with serial imaging, the association of single organ MRI abnormalities with organ-specific symptoms, the association of multiorgan MRI abnormalities with inflammatory markers, genetic associations with multiorgan MRI abnormalities, multivariate determinants of multiorgan MRI abnormalities, and the association of multiorgan MRI abnormalities with adverse clinical outcomes at 2-year and 5-year follow-up.

### Statistical analyses

Based on conservative estimates of multiorgan injury (27% of patients and 9% of matched controls from pilot data), a minimum sample size of 306 (255 cases and 51 controls) was estimated to give the study a power of 90% for 2-sided α level of 0·05.

The primary outcome was the difference in the proportion of individuals with multiorgan abnormalities (ie, abnormalities in two or more organs), a binary variable, between cases and controls. For other exploratory outcomes, correction for multiple comparisons was not applied. Statistical analysis was conducted with R (version 4.1.0) and R Studio (version 1.4.1717). Categorical summary statistics are presented as counts and percentages of non-missing data. When continuous measures were skewed, we report median and IQR; otherwise, mean and SD are reported.

We assessed group-wise differences across multiorgan imaging metrics between controls and patients at follow-up, as well as across cohort characteristics between patients with and without organ abnormalities. In univariate analyses, χ^2^ and Fisher’s exact tests were used to compare proportions where appropriate. When continuous measures were skewed, a Mann-Whitney U test was used; otherwise, a two-group Welch test was used to compare group means.

To assess multivariable group-wide differences between patients and controls, linear and logistic regression analyses were applied with inverse probability weighting to adjust for age, sex, BMI, smoking, hypertension, hypercholesterolaemia, diabetes, pre-existing comorbidities (cardiac, brain, liver, lung, and renal), and scanner manufacturer. These covariates were selected given their association with imaging abnormalities in previous work.^14^ Continuous imaging outcomes were scaled for normality when necessary, and differences in brain imaging outcomes were additionally adjusted by head size, date of imaging, and scanner table position. Differences associated with follow-up abnormality status were assessed with linear (β coefficient) and logistic (odds ratio [OR]) regression models adjusted by age, sex, smoking, obesity, hypertension, Charlson Comorbidity Index, and scanner manufacturer. We additionally undertook sensitivity analysis by excluding individuals who had a COVID-19 WHO clinical progression scale of 7–9 during admission to examine whether our associations remained robust to this exclusion.

### Role of funding source

The funder of the study had no role in study design, data collection, data analysis, data interpretation, or writing of the report.

## Results

**Figure 1:**
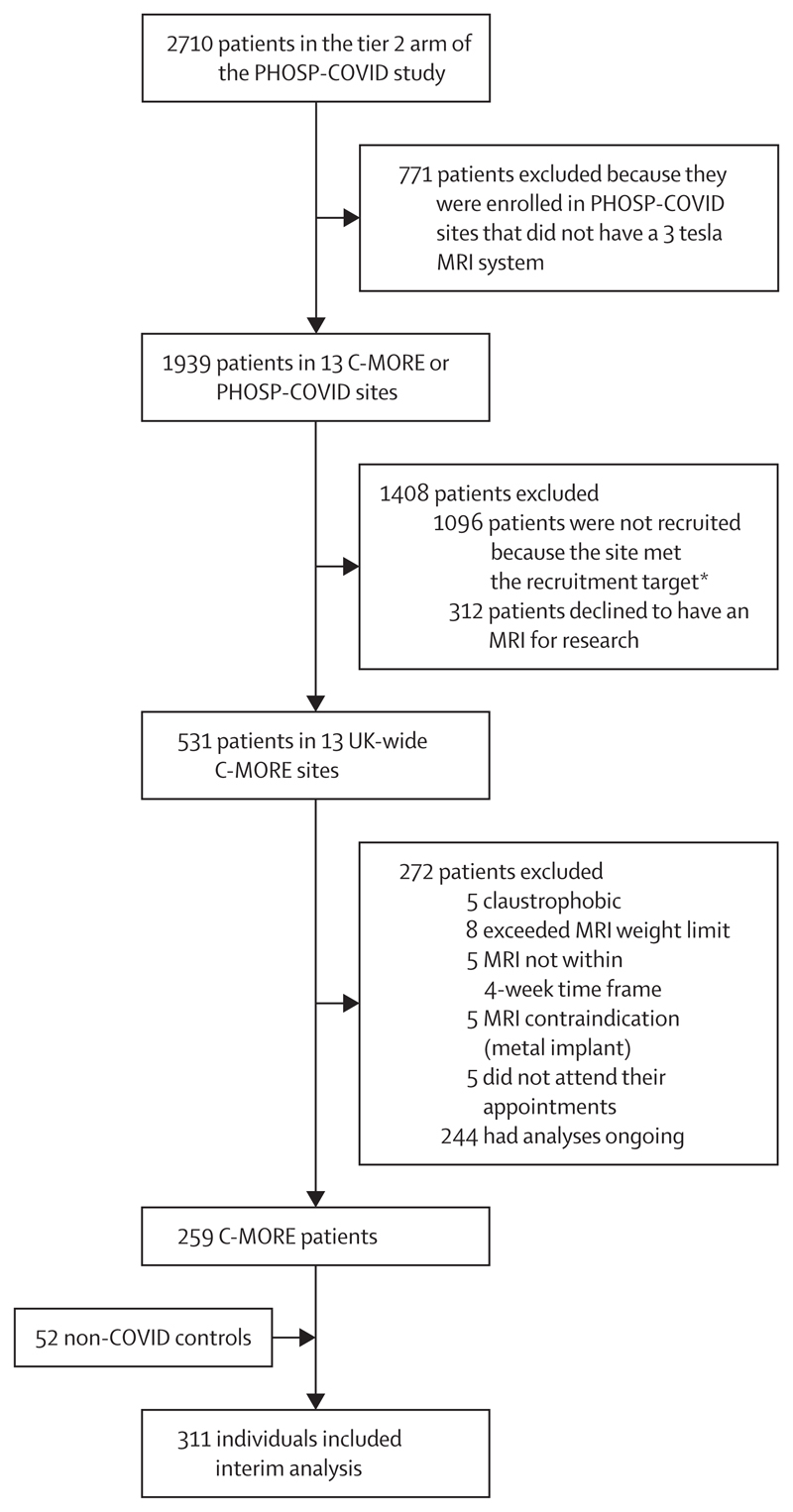
Study profile C-MORE=Capturing Multiorgan Effects of COVID-19 study. PHOSP-COVID=Post-hospitalisation COVID-19 study. *Each site was expected to contribute a minimum number of participants, based on capacity. When the site met their target recruitment, they stopped co-enrolling patients into C-MORE from PHOSP-COVID.

Of the 2710 patients in Tier 2 of PHOSP-COVID, 531 patients were recruited across 13 UK-wide C-MORE sites. 259 consecutive C-MORE patients and 52 non-COVID-19 controls were included in this interim analysis. Patient flow through the study is presented in figure 1, and baseline characteristics are shown in table 1. The median time from hospital discharge to MRI scan was 5·0 months (IQR 4·2–6·3). Of the 259 patients, 101 (39%) were female and 72 (28%) were non-White; 22 (42%) of the 52 controls were female and 14 (27%) were non-White. Patients were older than controls, with similar BMI, and were more likely to be ex-smokers than were controls (table 1). 69 (27%) of 255 patients had severe SARS-CoV-2 infection (WHO class ≥6). 191 (75%) of 254 patients were treated with steroids, 132 (52%) of 255 with anticoagulation therapy, and 29 (25%) of 115 with antiviral therapy (remdesivir). Further details on cohort characteristics are provided in the appendix (p 12, 23).

81 (32%) of 257 patients with COVID-19 reported a pre-existing respiratory condition, compared with eight (15%) of 52 controls (table 1). During hospital admission, 141 (55%) of 255 patients needed supplemental oxygen by mask or nasal prongs (WHO class 5), 51 (20%) of 255 required high-flow or non-invasive ventilation (WHO class 6), and 18 (7%) of 255 required invasive ventilation (WHO classes 7–9). The remaining 45 (18%) of 255 patients did not need oxygen therapy during hospital admission (WHO classes 3–4).

At a median time of 5 months after hospital discharge, more patients had lung MRI abnormalities affecting more than 5% of parenchyma (90 [35%] of 259) than did controls (three [6%] of 49; p_adjusted_=0·0008 from multivariable models in which the effect of the variable of interest is adjusted for covariates; table 2). Of the 90 patients with an abnormal lung MRI, 65 (72%) had no previous respiratory disease. Semi-quantitative analyses detected higher mean T2 signal (p_adjusted_=0·0009) and greater heterogeneity in T2 signal among patients relative to controls (p_adjusted_=0·0004), a finding also evident when patients with critical COVID-19 (WHO class 7–9) were excluded (appendix p 24). Mean global pulmonary blood volume, blood flow, and transit time were not different between patients and controls (p_adjusted_>0·20 for all; appendix p 24).

Follow-up lung function assessment revealed more pulmonary function defects among patients than controls. The mean FEV_1_ and FVC were lower in patients than in controls (p_adjusted_<0·0001 for both), whereas the ratio of FEV_1_ to FVC was higher in patients, in keeping with a restrictive lung function pattern (p_adjusted_=0·0050). 30 (23%) of 131 patients had abnormal FEV_1_ (<80% of predicted normal FEV_1_) and 29 (22%) of 131 had abnormal FVC, compared with one (4%) of 28 controls with abnormal FEV_1_ and none with abnormal FVC (table 2)

**Figure 2:**
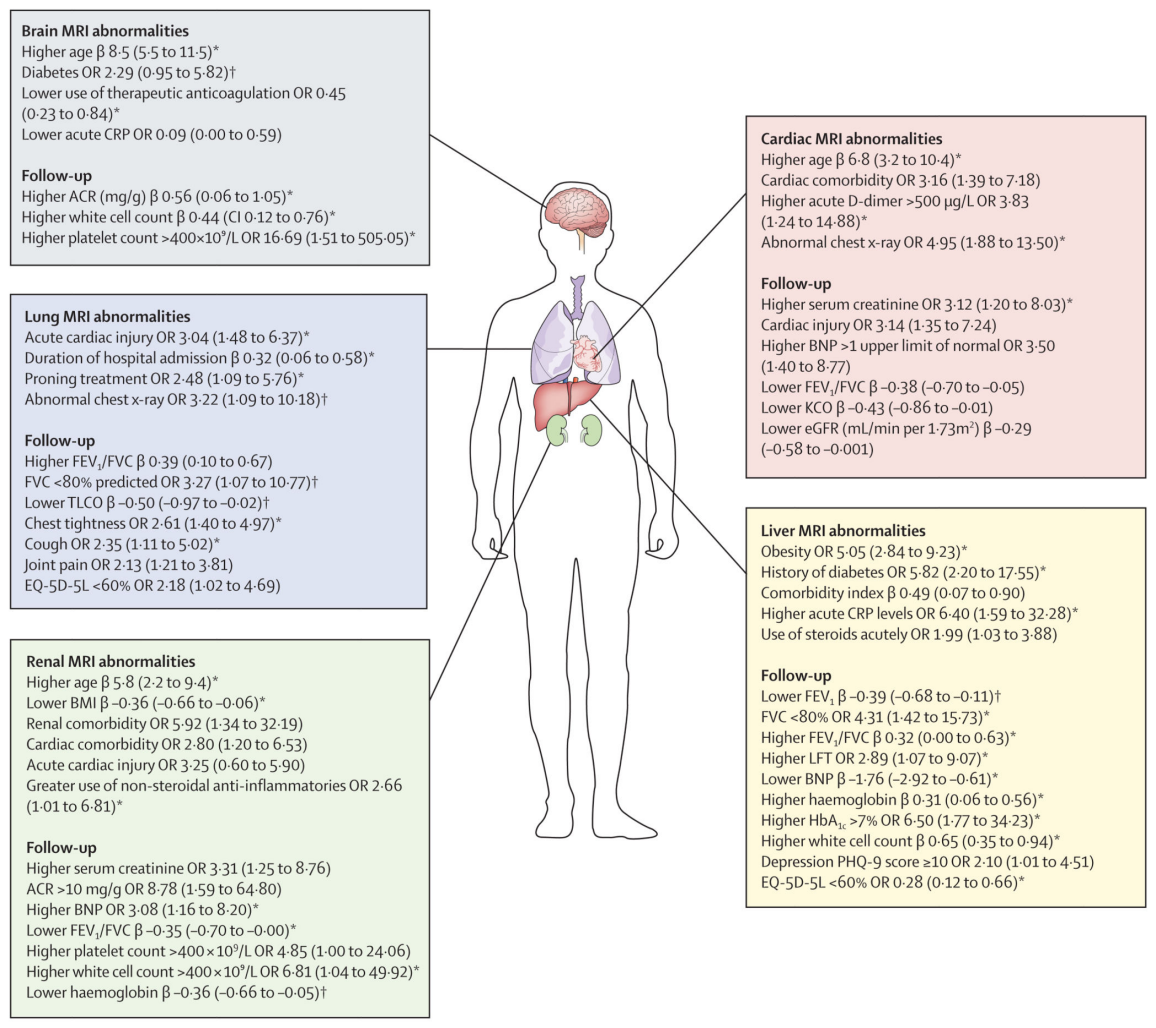
Factors associated with single-organ injury at follow-up with corresponding effect size A summary of the key clinical characteristics of patients that were associated with single-organ injury, including follow-up clinical measures. All associations (other than age in years) are adjusted for difference in age, sex, smoking, hypertension, Charlson Comorbidity Index, obesity, and scanner manufacturer. Standardised β coefficients (unit’s standard deviations) and ORs are depicted in the diagram with 95% CI in brackets. ACR=albumin to creatinine ratio. BNP=B-type natriuretic peptide. CRP=C-reactive protein. eGFR=estimated glomerular filtration rate. EQ-5D-5L=EuroQol 5-level. FVC=forced vital capacity. KCO=carbon monoxide transfer coefficient. LFT=liver function test. OR=odds ratio. PHQ-9=Patient Health Questionnaire-9. TLCO=transfer factor of the lung for carbon monoxide.*Associations present after excluding patients with corresponding organ comorbidities. †Association only present after excluding patients with corresponding organ comorbidities.

Among all patients, lung MRI abnormalities were independently associated with acute factors including longer duration of hospital admission, acute cardiac injury, and prone positioning (marker of severe hypoxaemia). At follow-up, patients with lung MRI abnormalities had lower FEV_1_ and higher FEV_1_/FVC ratio than did patients without lung MRI abnormalities (appendix p 24; figure 2). Among patients, heterogeneity in lung parenchymal signal on MRI (appendix p 28) was also associated with persistently raised C-reactive protein (CRP; ≥5 mg/L). After excluding patients with pre-existing lung disease, all associations remained, except that patients with lung MRI abnormalities were more likely to have FVC of less than 80% predicted and lower TLCO and an abnormal chest-x-ray than patients without lung MRI abnormalities (appendix p 26). Among all patients, lung MRI abnormalities were associated with patient reported outcome measures of chest tightness, joint pain, and impaired quality of life (appendix p 25). After excluding pre-existing lung disease, an abnormal lung MRI was associated with cough and chest tightness (appendix p 26).

Patients had a higher burden of cardiac comorbidities (40 [16%] of 257) relative to controls (two [4%] of 52; p=0·043; table 1). Acute myocardial injury (defined as a troponin, B-type natriuretic peptide [BNP], or N-terminal pro-BNP [NT-proBNP] concentration of more than 1 × upper limit of normal [ULN]) was reported in 38 (15%) of 255 patients. At follow-up, abnormal troponin was seen in five (4%) of 139 patients, and abnormal NT-pro BNP in 32 (18%) of 174 patients (appendix p 29).

Abnormal cardiac MRI findings were seen in 54 (21%) of 259 patients and 12 (24%) of 49 controls (p_adjusted_=0·30; table 2). Biventricular indexed stroke volumes were smaller in patients than in controls, and right ventricular function, mean myocardial T1 and T2, and mean extracellular volume fraction were similar between patients and controls (appendix p 29?). Although within the normal range, patients had lower mean left ventricular ejection fraction (60·5% [SD 6·0] *vs* 62·9% [5·5] in controls; p_adjusted_=0·048). 18 (7%) of 256 patients and one (2%) of 49 controls had left ventricular dysfunction (ejection fraction <52%) and 11 (4%) of 254 and one (2%) of 49 controls had right ventricular dysfunction (ejection fraction <48%; appendix p 29).

34 (14%) of 251 patients had a pathological pattern of LGE, similar to controls (six [12%] of 49; p_adjusted_=0·42). Among patients, 22 (9%) of 251 had a possible or probable acute or previous myocarditis LGE pattern versus six (12%) of 49 controls (p_adjusted_=0·050). 14 (6%) of 251 of patients had a possible or probable ischaemic LGE pattern versus one (2%) of 49 controls (p_adjusted_=0·80), and two (1%) of 251 had a mixed LGE pattern versus zero controls (p=1·0). Pericardial effusion was seen in three (1%) of 251 patients versus one (2%) of 49 controls (p=0·51; appendix p 29). Active myocarditis, as per the updated Lake Louise criteria^15^ (increase in both myocardial T1 and T2), was rare, affecting only four (1%) of 259 of patients at a median follow-up of 5 months versus three (6%) of 52 controls.

Of the patients with cardiac MRI abnormalities, 18 (33%) of 54 had pre-existing cardiac disease, and 11 (20%) of 54 had evidence of acute cardiac injury during hospital admission. Factors associated with cardiac MRI abnormalities in all patients included older age, cardiac comorbidity, abnormal chest x-ray, and elevated acute D-dimer levels. At follow-up, patients with cardiac MRI abnormalities were more likely to have increased NT-proBNP, reduced renal function or eGFR, and reduced lung function (FEV_1_/FVC ratio and carbon monoxide transfer coefficient) compared with patients without cardiac MRI abnormalities (figure 2; appendix p 30). After excluding patients with cardiac comorbidities, older age, abnormal chest x-ray, acute and follow-up elevated D-dimer, and follow-up renal impairment remained associated with cardiac MRI abnormalities (appendix p 31). Cardiac MRI abnormalities were not associated with patient-reported outcome measures, including symptoms of chest pain or breathlessness.

Pre-existing neurological diagnoses were similar between patients (nine [4%] of 257) and controls (one [2%] of 52; p_adjusted_=0·43; table 1). At follow-up, qualitative brain MRI abnormalities were noted in 109 (50%) of 218 patients versus nine (18%) of 50 controls (p<0·0001, p_adjusted_=0·0029; table 2). White matter hyperintensities and small vessel disease were more common among patients, who also had smaller grey matter volumes relative to controls, specifically in areas important for higher cognitive function, including memory and emotional processing and autonomic nervous function (hippocampus, amygdala, cerebellum, and thalamic nuclei), motor control (bilateral putamen), audiovisual processing (bilateral middle temporal gyrus), visual processing (bilateral cuneal and intracalcarine cortex) and wakefulness or consciousness, and thermoregulation (thalamic nuclei). Patients also had lower regional brain volumes involving areas important for spatial memory formation (left posterior cingulate cortex), language processing and perception (supramarginal cortex), and pain perception (insula; appendix p 33). Total brain white matter volume did not significantly differ between patients and controls. Brain MRI abnormalities were more common among patients even after excluding patients who were critically ill with COVID-19 (appendix p 33).

Of patients with brain MRI abnormalities, only five (5%) of 109 had pre-existing known neurological disease. Older age, diabetes, lower acute CRP, higher bilirubin, and lower use of therapeutic anticoagulation were associated with brain MRI abnormalities among patients (appendix pp 34–35; figure 2). At follow-up, higher white cell count, higher urinary albumin to creatinine ratio, and higher platelet count were associated with brain MRI abnormalities. These associations remained even after excluding cases with pre-existing neurological disease (appendix p 36). Of note, persistently raised CRP (≥5 mg) was associated with regional brain atrophy involving areas important for memory, emotional processing (amygdala nuclei), audiovisual processing (superior, middle, and inferior temporal gyrus), control of autonomic functions (brainstem), and visuospatial memory (cuneus) among patients (appendix p 28). Qualitative and quantitative brain MRI abnormalities were not linked to any patient-reported outcome measure after confounder adjustment.

14 (5%) of 257 patients reported pre-existing liver diagnoses, and no known liver comorbidities were reported in controls (table 1). Acute liver biochemistry (combining alanine transaminase, alkaline phosphatase, bilirubin, or gamma-glutamyl transferase) was abnormal in 141 (58%) of 244 patients during the acute phase (in hospital). At follow-up, liver biochemistry was abnormal (>1 × ULN) in 30 (14%) of 221 patients and 16 (31%) of controls (appendix p 38). Abnormal liver MRI findings (ie, elevated liver inflammation, liver iron, or fat) were frequent among patients but similarly prevalent among controls (p_adjusted_=0·31; table 2). Patients had mean liver cT1 (p_adjusted_=0·47) and liver fat (p_adjusted_=0·19) similar to those of controls, but lower liver iron (p_adjusted_<0·0001; appendix p 38).

Among all patients, liver MRI abnormalities were associated with obesity, diabetes, higher Charlson Comorbidity Index, acute CRP of more than 5 mg/L, and acute steroid treatment. At follow-up, patients with liver MRI abnormalities had higher serum alanine transaminase, poorer lung function (lower FEV_1_, FVC, and higher FEV1/FVC ratio), lower BNP, higher HbA1_c_, and higher haemoglobin and white cell count than did patients without MRI abnormalities (appendix p 38; figure 2). After excluding patients with pre-existing liver disease, all associations remained except those with acute CRP and steroid treatment (appendix p 39). There were no associations between liver MRI abnormalities and abdominal or gastrointestinal symptoms, cognitive impairment, or breathlessness. However, patients with liver MRI abnormalities were more likely to report symptoms of depression and less likely to report an impaired quality of life than those without liver MRI abnormalities.

14 (5%) of 257 patients had pre-existing kidney disease versus one (2%) of 52 controls (p_adjusted_=0·44; table 1). During the acute phase in hospital, 42 (17%) of 255 patients with COVID-19 had acute kidney injury (appendix p 41). At follow-up, renal impairment (eGFR <60 mL/min per 1·73 m^2^) persisted in 13 (6%) of 220 patients versus none of the controls. Renal abnormalities on MRI were more frequent among patients (57 [23%] of 246 *vs* three [6%] of 48 controls; p=0·014; table 2). Total renal volumes indexed to body surface were similar between patients and controls. The mean renal medullary T1 was lower in patients (1895 ms [SD 88] *vs* 1935 ms [72]; p=0·0021), and cortical T1 did not differ between patients and controls (p=0·14). Mean renal corticomedullary differentiation, a marker of renal microstructural health, was lower in patients (371 ms [SD 58] *vs* controls 402 ms [50]; p=0·0012). After adjusting for confounders, renal MRI abnormalities tended to be more frequent in patients (OR 2·36 [95% CI 0·89–8·02]) than in controls (appendix p 41).

Of the patients with abnormal renal MRI, eight (14%) of 57 had pre-existing known renal disease, and 16 (29%) of 56 had acute kidney injury during admission. In all patients, renal MRI abnormalities were associated with older age, lower BMI, comorbid conditions including renal and cardiac disease, higher acute cardiac troponin, and use of non-steroidal anti-inflammatories. At follow-up, patients with renal MRI abnormalities were more likely to have higher serum creatinine, urinary albumin and creatinine ratio of more than 10 mg/g, higher serum NT-proBNP, lower FEV_1_/FVC ratio, higher platelet counts, lower white cell count, and a tendency to lower haemoglobin when compared with patients without MRI abnormalities (appendix p 42; figure 2). In patients without pre-existing renal disease, all associations remained except those with platelet count, serum creatinine, and urinary albumin to creatinine ratio (appendix p 43). There were no associations between renal MRI abnormalities and patient-reported outcome measures.

**Figure 3:**
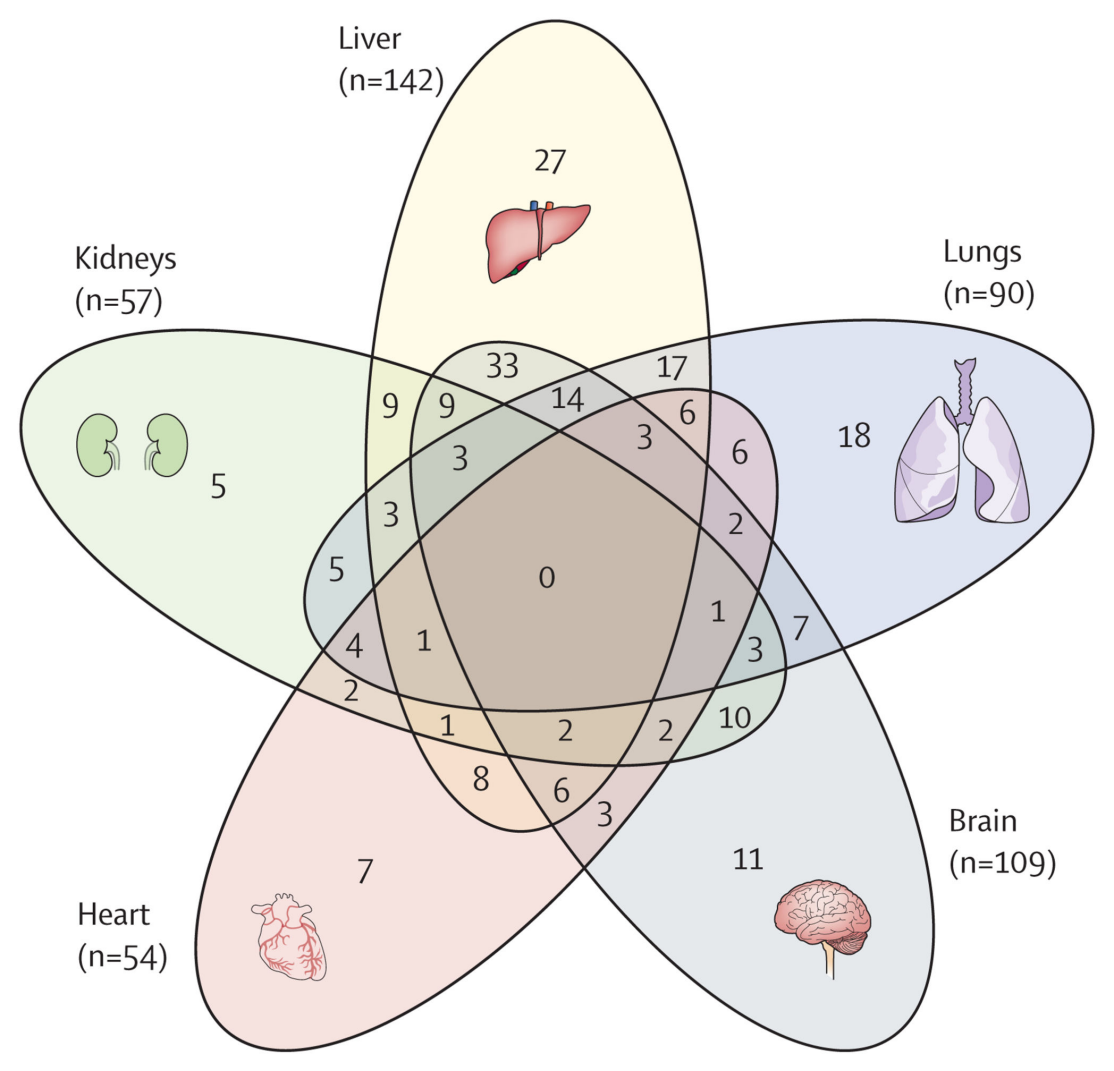
Venn diagram showing overlap of organ abnormalities on MRI after hospitalisation for COVID-19 and the most common triad of organ abnormalities Numbers in brackets represent the absolute number of people with relevant organ abnormalities on MRI.

**Figure 4:**
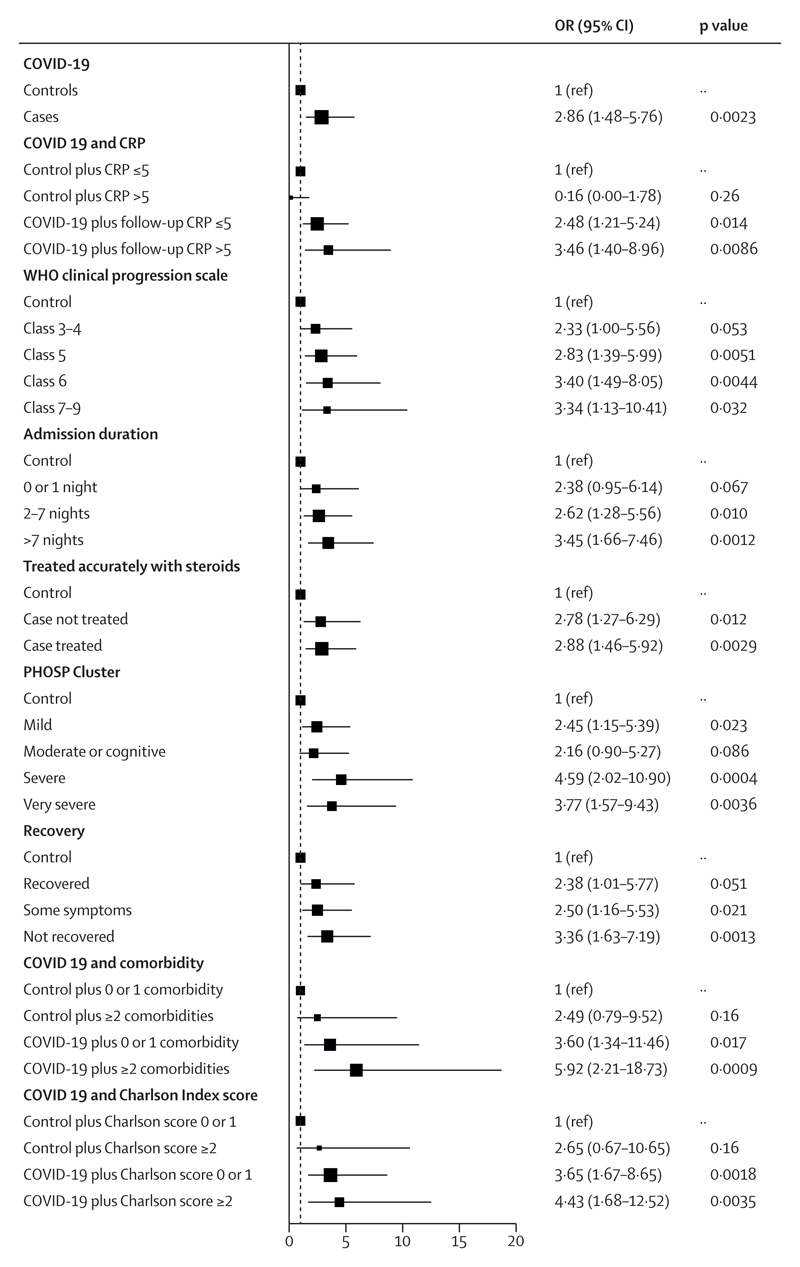
Determinants of multiorgan injury on MRI among post-hospitalised patients recovering from COVID-19 Forest plot depicts the effect of hospitalisation for COVID-19 on medium-term multiorgan (ie, two or more organs) health stratified by WHO severity, comorbidity status, severity of acute infection, inflammatory burden, and recovery status relative to controls. This analysis is adjusted for pre-existing comorbidities. ORs and 95% CIs are from logistic regression models with multiorgan injury as the outcome and the displayed variable as the exposure. Associations are adjusted by age, sex, BMI, smoking, scanner manufacturer, existing risk factors (hypertension, diabetes, high cholesterol), and pre-existing disease (cardiac, neurological, respiratory, kidney, and liver disease). CRP=C-reactive protein. OR=odds ratio. PHOSP=Post-hospitalisation COVID-19 study.

Overall, after hospitalisation for COVID-19, patients had a higher burden of multiorgan MRI abnormalities than did controls (157 [61%] of 259 *vs* 14 [27%] of 52; p<0·0001; table 2). The combination of lung, liver, and brain abnormalities was the most common combination of organ abnormalities (figure 3), probably reflecting a combination of pre-existing (for liver) and post-COVID-19 injury. After hospitalisation for COVID-19, patients were at higher risk (OR 2·9 [95% CI 1·5–5·8]; p_adjusted_=0·0023) of abnormalities in two or more organs than were controls after adjusting for pre-existing comorbidities (figure 4; table 2) or for Charlson Comorbidity Index (appendix p 23). Patients with multiorgan abnormalities were older (difference in mean ages 7 years [95% CI 4–10]; mean age of 59·8 years (SD 11·7) with multiorgan abnormalities *vs* mean age of 52·8 years (11·9) without multiorgan abnormalities; p<0·0001), more likely to have three or more comorbidities (OR 2·47 [1·32–4·82]; p_adjusted_=0·0059), and more likely to have a more severe acute infection (acute CRP >5mg/L, OR 3·55 [1·23–11·88]; p_adjusted_=0·025) than those without multiorgan abnormalities.

Patients with more severe acute COVID-19 disease (WHO clinical progression score ≥6) had the highest risk of multiorgan MRI abnormalities compared with controls. However, even after excluding individuals with critical COVID-19 (WHO 7–9), multiorgan abnormalities were more frequent in patients than controls (table 2, figure 4). Acute treatment with steroids in hospital was not associated with a lower burden of multiorgan abnormalities among patients. Patients with a follow-up CRP of 5 mg/L or less, and those with CRP of more than 5 mg/L, were at a two-times and three-times increased risk of multiorgan MRI abnormalities, respectively, compared with controls with normal or abnormal CRP (figure 4).

Patients from the severe and the very severe PHOSP-COVID symptom clusters (indicating severe and very severe persistent mental and physical health impairment) were four times more likely to have multiorgan MRI abnormalities than were controls (figure 4).

## Discussion

Our study demonstrates the substantial burden of multiorgan abnormalities in patients after hospitalisation for COVID-19, with nearly one in three patients having an excess burden of multiorgan injury. When compared with controls, we noted a higher proportion of lung, brain, and renal MRI abnormalities among patients. Multiorgan abnormalities on imaging were associated with older age, comorbidities, and severity of acute infection, with evidence of both vascular and inflammatory patterns of injury. We found that lung MRI abnormalities were associated with symptoms of chest tightness and impaired lung function, and multiorgan abnormalities were linked to persistent severe and very severe physical and mental health impairment (PHOSP-COVID recovery clusters) at 5 months after hospital discharge.

Follow-up studies of patients after hospitalisation for COVID-19 have previously found a high prevalence of pulmonary diffusion abnormalities,^16^ with a meta-analysis of pooled data from 15 studies suggesting that 32·6% of patients exhibit ground glass pulmonary interstitial changes up to 12 months after infection.^17^ In the present study, an excess of 28% of patients had lung MRI abnormalities, which were also associated with impaired gas transfer (TLCO). Although longitudinal studies^18,19^ suggest that such abnormalities can resolve over time, 10% of individuals have been seen to develop pulmonary fibrosis by 2 years after acute infection.^20^ Tropism of SARS-CoV-2 for endothelial cells^21^ and pericytes^22^ has led to speculation that pulmonary vascular dysfunction might prevail in the long term. Numerous retrospective investigations^1,23,24^ have shown an increased risk of arterial and venous thrombosis, with COVID-19 hospitalisation conferring the highest risk. To our knowledge, our study is the first multicentre study to evaluate pulmonary perfusion as assessed on MRI in a large post-hospitalised COVID-19 patient cohort and, contrary to expectations, pulmonary perfusion measures (global pulmonary blood flow, blood volume, or mean transit time) did not differ between patients and controls. Our findings suggest that complex mechanisms probably underpin pulmonary perfusion changes seen after COVID-19.

The medium-term to long-term effects of COVID-19 on the heart have been a subject of intense debate.^25^ Early imaging studies of convalescent patients raised concerns about a high burden of myocarditis.^26^ However, subsequent post-mortem studies and cardiac biopsies failed to confirm these findings.^27^ In the present study, blinded image analyses by an experienced core lab team found comparable abnormalities in patients and controls. Specifically, a diagnosis of myocarditis was no more common in patients than in controls, and cardiac MRI abnormalities did not predict ongoing symptoms. By contrast, patients did exhibit reduced left ventricular systolic function relative to controls. Investigators of the Hamburg City Health Study^28^ also noted a significant reduction in cardiac function on follow-up transthoracic echocardiography in non-hospitalised patients. Although these findings are intriguing, unmeasured residual confounders might contribute to differences and, whereas the routine use of transthoracic echocardiography might be sufficient as a screening tool, cardiac MRI might be more appropriate if the pretest probability of ongoing ischaemic or inflammatory injury is high.^29^

Cognitive impairment and brain fog are common manifestations after acute COVID-19. In a study of UK Biobank participants with brain MRI before and after SARS-CoV-2 infection, detailed quantitative analyses revealed grey matter volume reduction and abnormal diffusion parameters in regions of the brain involved in olfactory signal processing and memory.^30^ In the present study, we noted a high burden of white matter hyperintensities and small vessel disease in patients not specifically referred for neurological symptoms, a finding that persisted after excluding severe cases and adjusting for pre-existing neurological diagnoses. Our findings suggest that vascular patterns of injury are common, and that patients with more severe infections have lower grey matter volumes^30^ involving multiple regions of the brain relevant to working memory, emotion, visuo-auditory processing, and autonomic nervous function. By contrast, clinically evident MRI abnormalities were not associated with cognitive function. These findings highlight the diminished neurological reserve and vulnerability of post-hospitalised patients with COVID-19 for future insults.

Histologically validated, quantitative liver MRI metrics are gaining importance as prognostically relevant surrogates of liver fat and inflammation in infectious and metabolic diseases.^13^ In one previous study of non-hospitalised patients with long COVID, liver fat was associated with breathlessness.^31^ In the present study, there were similar rates of liver abnormalities among patients and controls, and liver abnormalities were not significantly associated with gastrointestinal symptoms and breathlessness, implying that the liver might not be a major organ contributing to persistent post-COVID-19 symptoms.

Follow-up studies of patients hospitalised with COVID-19 report a decline in renal function in up to 30% of patients.^16^ A retrospective study of electronic health records from the US Department of Veterans Affairs national health-care databases observed a 13% increase in relative risk of renal impairment among patients who were not hospitalised.^32^ Others^8,33^ have also noted abnormalities in the renal cortex and medulla on renal MRI. As the largest multicentre study examining renal health on MRI, we noted that 23% of our patients had renal abnormalities. Specifically, corticomedullary differentiation, a reliable marker of renal health, was reduced in patients, and MRI abnormalities were associated with follow-up renal function. Our study also newly reports the determinants of renal MRI abnormalities, aligning well with factors that predict long-term renal outcomes in other studies.^34^

Although lung MRI abnormalities were associated with chest tightness and impaired lung function, there was a surprising disconnect between other organ injury and persistent symptoms, underscoring the complex biology that underlies long COVID. However, when evaluating the impact of multiorgan injury on persistent severe and very severe physical and mental health impairment, a novel association was noted relative to controls. Therefore, although the yield of multiorgan MRI for elucidating organ-specific symptoms is uncertain, a normal MRI might provide reassurance to patients. Indeed, this hypothesis is currently being investigated in a randomised UK study of non-hospitalised, post-COVID-19 patients called STIMULATE-ICP.^35^

Severe SARS-CoV-2 infections are declining following widespread vaccination. However, older vulnerable patients globally still require hospital care and millions continue to suffer from the long-term complications of COVID-19. International consensus on multidisciplinary follow-up care pathways remains opaque.^36^ As the most comprehensive follow-up MRI study so far, we provide important insights into the considerable burden of persistent multiorgan pathology, with implications for ongoing management. Specifically, impaired patient recovery was linked to multiorgan dysfunction after COVID-19, and follow-up multidisciplinary services should focus on pulmonary, renal, vascular, and neurological health in the long term.^35^

Although biochemical and MRI abnormalities were associated in some organs, neither biochemistry nor an absence of symptoms could exclude underlying imaging abnormalities, highlighting the value of more sensitive imaging-based assessments. Although the long-term functional implications of some MRI abnormalities require clarification, many of these markers have been shown to associate with poor clinical outcomes in other diseases, reminding us to be vigilant of the future risk of moderate to severe COVID-19.

Our study is ongoing, and this early report is intended to guide clinical follow-up for patients. The study is limited by its small sample size, inadequate power to examine multiple associations, and susceptibility to acquisition and survival bias. Our controls were not previously hospitalised, but this study aimed to describe the excess burden of abnormalities in patients hospitalised with acute COVID-19 relative to a general non-COVID-19 population. Lung MRI is less precise than CT for the quantification of lung parenchymal abnormalities, and might have underestimated the prevalence of lung abnormalities. Recent work from our consortium also suggests that lung perfusion after COVID-19 improves longitudinally on MRI at 1·5 Tesla, and thus the timing of the scan might be relevant when assessing perfusion measures.^37^

Our patients were younger, less obese, and less likely to need intensive care or mechanical ventilation than individuals who currently need hospital admission for COVID-19; hence, the burden of multiorgan dysfunction is likely to be an underestimate of the current burden of post-hospitalisation multiorgan dysfunction. Missing data was variable across parameters, but the study was adequately powered for its primary endpoint. The study’s cross-sectional design did not allow us to distinguish premorbid disease from infection-specific emergent manifestations, and only patients with non-omicron SARS-CoV-2 variants were enrolled, limiting the generalisability of our findings. Additionally, factors related to hospitalisation alone might have contributed to organ abnormalities (compared with non-hospitalised controls), and thus further studies with post-hospitalised control patients secondary to non-COVID-19 infections are needed.

In summary, in our prospective MRI study of post-hospitalised patients with COVID-19, multiorgan MRI abnormalities were about three times more common among patients after hospital discharge than in uninfected, non-hospitalised controls, and linked to persistent severe and very severe physical and mental health impairment. Both inflammatory and vascular or haemostatic patterns of injury were observed across some organs. These findings underscore the need for multi-targeted therapies and integrated multidisciplinary follow-up services for patients recovering after hospital admission with COVID-19.

## Supplementary Material

Supplementary appendix

## Figures and Tables

**Table 1:** Baseline characteristics of patients with COVID-19 discharged from hospital and non-COVID-19 controls

	Controls (n=52)	Patients with COVID-19 (n=259)	p value	Univariate test	Multivariable analysis adjusted inverse probability weighting*		Multivariable analysis adjusted inverse probability weighting* excluding patients with WHO clinical progression score ≥7	
					OR or β coefficient (95% CI)	p value	OR or β coefficient (95% CI)	p value
Age, years	49·3 (13·9)	57·0 (12·2)	0·0004	Welch T	· ·	··	· ·	· ·
Sex	··	··	0·76	Fisher’s exact	··	··	··	··
Female	22/52 (42%)	101/259 (39%)	··	··	··	··	··	··
Male	30/52 (58%)	158/259 (61%)	··	··	··	··	··	··
Ethnicity	··	··	1·0	χ^2^	OR 3·06 (1·35 to 8·05)	0·013	OR 2·73 (1·20 to 7·24)	0·026
Non-White	14/52 (27 %)	72/259 (28%)	··	··	··	··	··	··
White	38/52 (73%)	187/259 (72%)	··	··	··	··	··	··
BMI, kg/m^2^	28·8 (7·5)	30·6 (5·8)	0·11	Welch T	β 0·03 (–0·29 to 0·34)	0·87	0·05 (–0·26 to 0·37)	0·74
Obesity (>30 kg/m^2^)	19/52 (37%)	129/259 (50%)	0·11	χ^2^	OR 1·11 (0·59 to 2·11)	0·75	OR 1·17 (0·62 to 2·22)	0·64
BMI category	··	··	0·0062	Fisher’s exact	··	··	··	··
<25 kg/m^2^	18/52 (35%)	36/259 (14 %)	··		··	··	··	··
25–29 kg/m^2^	15/52 (29%)	93/259 (36%)	··	··	··	··	··	··
30–34 kg/m^2^	10/52 (19%)	79/259 (31%)	··	··	··	··	··	··
35–39 kg/m^2^	4/52 (8%)	36/259 (14%)	··	··	··	··	··	··
>40 kg/m^2^	5/52 (10%)	15/259 (6%)	··	··	··	··	··	··
Systolic blood pressure, mm Hg	134·9 (21·2)	133·0 (15·3)	0·55	Welch T	β –0·48 (–0·80 to –0·16)	0·0034	–0·47 (–0·78 to –0·15)	0·0042
Smoking status	··	··	0·0011	Fisher’s exact	··	··	··	··
Never smoked	43/52 (83%)	155/259 (60%)	··	··	··	··	··	··
Ex smoker	6/52 (12%)	92/259 (36%)	··	··	··	··	··	··
Current smoker	3/52 (6%)	12/259 (5%)	··	··	··	··	··	··
Alcohol intake, units per week	1·00 (0·00 to 1·00)	1·50 (0·00 to 6·50)	0·012	Mann-Whitney U	β 0·32 (–0·00 to 0·63)	0·050	0·31 (–0·01 to 0·63)	0·056
Index of multiple deprivation								
1, most deprived	Not available	53/254 (21%)	··	··	··	··	··	··
2	Not available	49/254 (19%)	··	··	··	··	··	··
3	Not available	40/254 (16%)	··	··	··	··	··	··
4	Not available	60/254 (24%)	··	··	··	··	··	··
5, least deprived	Not available	52/254 (20%)	··	··	··	··	··	··
Pre-existing comorbidities								
Diabetes	7/52 (13%)	55/257 (21%)	0·27	χ^2^	OR 0·57 (0·28 to 1·19)	0·13	OR 0·58 (0·28 to 1·22)	0·14
High cholesterol	7/52 (13%)	46/257 (18%)	0·57	χ^2^	OR 0·79 (0·37 to 1·81)	0·55	OR 0·75 (0·35 to 1·75)	0·49
Hypertension	15/52 (29%)	128/257 (50%)	0·0073	χ^2^	OR 0·88 (0·46 to 1·66)	0·68	OR 0·86 (0·45 to 1·64)	0·65
Respiratory comorbidity	8/52 (15%)	81/257 (32%)	0·030	χ^2^	OR 1·61 (0·79 to 3·50)	0·205	OR 1·71 (0·84 to 3·74)	0·16
Cardiac comorbidity	2/52 (4%)	40/257 (16%)	0·043	χ^2^	OR 1·67 (0·66 to 5·19)	0·32	OR 1·67 (0·65 to 5·20)	0·33
Neurological comorbidity	1/52 (2%)	9/257 (4%)	1·0	Fisher’s exact	OR 0·54 (0·13 to 3·12)	0·43	OR 0·59 (0·14 to 3·40)	0·50
Liver disease	0/52	14/257 (5%)	0·14	Fisher’s exact	Not calculable	0·99	Not calculable	0·99
Kidney disease	1/52 (2%)	14/257 (5%)	0·48	Fisher’s exact	OR 2·08 (0·44 to 25·06)	0·44	OR 1·87 (0·38 to 22·81)	0·51
Charlson Comorbidity Index								
Median score	0·00 (0·00 to 0·00)	0·00 (0·00 to 1·00)	0·0039	Mann-Whitney U	··	··	··	··
Score category	··	··	0·68	Fisher’s exact	··	··	··	··
0 OR 1	45/52 (87%)	215/259 (83%)	··	··	··	··	··	··
≥2	7/52 (13%)	44/259 (17%)	··	··	··	··	··	··
WHO clinical progression scale fOR COVID-19 severity								
WHO classes 3–4	Not applicable	45/255 (18%)	··	··	··	··	··	··
WHO class 5	Not applicable	141/255 (55%)	··	··	··	··	··	··
WHO class 6	Not applicable	51/255 (20%)	··	··	··	··	··	··
WHO classes 7–9	Not applicable	18/255 (7%)	··	··	··	··	··	··
Admission duration, days	Not applicable	6·0 (3·0 to 10·0)		··	··	··	··	··
Follow-up time, months	Not applicable	5·0 (4·2 to 6·3)	··	··	··	··	··	··
Vaccinated at follow-up	20/52 (40%)	112/255 (44%)	0·59	χ^2^	··	··	··	··
Treatments in acute phase								
Remdesivir	Not applicable	29/115 (25%)	··	··	··	··	··	··
Systemic ORal OR intravenous steroids	Not applicable	191/254 (75%)	··	··	··	··	··	··
Therapeutic dose anticoagulation	Not applicable	132/255 (52%)	··	··	··	··	··	··
Non-steroidal anti-inflammatory	Not applicable	28/255 (11%)	··	··	··	··	··	··

Data are n/N (%), mean (SD) for normally distributed continuous variables, or median (IQR) if not normally distributed, unless stated otherwise. OR=odds ratio.

* Inverse probability weighting was used to adjust imaging variable for confounders which included age, sex, BMI, smoking, hypertension, hypercholesterolaemia, diabetes, and cardiac, brain, liver, lung, and renal comorbidities; ORs were calculated by logistic regression and standardised β coefficients by linear regression.

**Table 2:** Comparison of clinical and selected MRI findings of patients with COVID-19 discharged from hospital and non-COVID-19 controls

	Controls (n=52)	Patients with COVID-19 (n=259)	p value	Univariate test	Multivariable analysis adjusted inverse probability weighting*		Multivariable analysis adjusted inverse probability weighting* excluding patients with WHO clinical progression score ≥7	
					OR or β coefficient (95% CI)	p value	OR or β coefficient (95% CI)	p value
Follow-up biochemistry or blood								
Follow-up CRP, mg/L	1·20 (0·65 to 2·40)	5·00 (1·90 to 5·00)	<0·0001	Mann-Whitney U	β 0·96 (0·67 to 1·26)	<0·0001	β 0·95 (0·66 to 1·25)	<0·0001
Follow-up eGFR, mL/min per 1·73 m^2^	90·0 (85·0 to 90·0)	90·0 (76·0 to 90·0)	0·016	Mann-Whitney U	β –0·37 (–0·70 to –0·04)	0·029	β –0·39 (–0·71 to –0·07)	0·019
Follow-up D-dimer, μg/L	266 (191 to 402)	227 (150 to 315)	0·029	Mann-Whitney U	β –0·45 (–0·79 to –0·11)	0·010	β –0·42 (–0·76 to –0·08)	0·016
Follow-up fibrinogen, g/L	2·80 (2–60 to 3·35)	3·40 (3·00 to 3·90)	<0·0001	Mann-Whitney U	β 0·38 (0·01 to 0·74)	0·043	β 0·41 (0·04 to 0·77)	0·029
Albumin to creatinine ratio, mg/mmol	0·15 (0·00 to 1·15)	1·30 (0·80 to 2·50)	0·0024	Mann-Whitney U	β 0·53 (–0·03 to 1·09)	0·061	β 0·53 (–0·03 to 1·08)	0·064
Serum creatinine, μmol/L	80·0 (64·5 to 86·5)	76·0 (64·0 to 87·0)	0·80	Mann-Whitney U	β –0·07 (–0·39 to 0·25)	0·67	β –0·07 (–0·39 to 0·26)	0·68
Follow-up lung function								
FEV1	3·71(0·98)	2·82(0·82)	<0·0001	Welch T	β –0·98 (–1·39 to –0·57)	<0·0001	β –0·96 (–1·37 to –0·55)	<0·0001
FVC	5·00(1·17)	3·61(0·97)	<0·0001	Welch T	β –1·22 (–1·62 to –0·82)	<0·0001	β –1·18 (–1·58 to –0·79)	<0·0001
FEV_1_/FVC	0·74 (0·06)	0·78 (0·09)	0·0044	Welch T	β 0·61 (0·19 to 1·03)	0·0050	β 0·57 (0·14 to 1·00)	0·0095
FEV_1_<80% of predicted	1/28(4%)	30/131(23%)	0·038	χ^2^	OR 13·91 (1·91 to 1178·93)	0·056	OR 13·3 (1·8 to 1126·4)	0·061
FVC <80% of predicted	0/28	29/131(22%)	0·013	χ^2^	··	0·99	··	0·99
FEV_1_/FVC ratio <0·7	4/28(14%)	24/174(14%)	1·0	Fisher’s exact	OR 1·23 (0·38 to 5·27)	0·75	OR 1·16 (0·35 to 5·02)	0·82
TLCO, mmol/min per kPa	Not applicable	7·79(2·11)	··	··	··	··	··	··
KCO, mmol/min per kPa	Not applicable	1·45 [1·29 to 1·60]	··	··	··	··	··	··
Participant-reported outcomes								
Cough	Not measured	77/231(33%)	··	··	··	··	··	··
Breathlessness	7/49(14%)	122/232(53%)	<0·0001	χ^2^	OR 3·95 (1·88 to 9·11)	0·0006	OR 3·74 (1·76 to 8·65)	0·0010
Chest pain	3/49(6%)	50/236(21%)	0·024	χ^2^	OR 6·77 (1·71 to 61·89)	0·025	OR 6·76 (1·83 to 66·48)	0·021
Chest tightness	Not measured	79/235(34%)	··	··	··	··	··	··
Palpitations	9/49(18%)	63/234(27%)	0·28	χ^2^	OR 1·07 (0·52 to 2·34)	0·86	OR 1·05 (0·50 to 2·32)	0·89
Abdominal pain	10/49(20%)	52/236(22%)	0·95	χ^2^	OR 0·83 (0·40 to 1·86)	0·64	OR 0·75 (0·35 to 1·70)	0·47
Nausea or vomiting	18/49(37%)	17/236(7%)	<0·0001	χ^2^	OR 0·20 (0·08 to 0·48)	0·00024	OR 0·18 (0·07 to 0·45)	0·00020
Diarrhoea	13/49(27%)	24/235(10%)	0·0043	χ^2^	OR 0·27 (0·11 to 0·69)	0·0054	OR 0·30 (0·12 to 0·75)	0·0094
Loss of control of passing urine	Not measured	25/236(11%)	··	··	··	··	··	··
Joint pain	28/49(57%)	106/235(45%)	0·17	χ^2^	OR 0·53 (0·27 to 1·02)	0·058	OR 0·50 (0·25 to 0·96)	0·041
Headache	20/49(41%)	91/235(39%)	0·91	χ^2^	OR 0·80 (0·42 to 1·58)	0·52	OR 0·71 (0·37 to 1·41)	0·32
Confusion or fuzzy head	Not measured	87/236(37%)	··	··	··	··	··	··
Difficulty with communication	Not measured	51/236(22%)	··	··	··	··	··	··
Dizziness or light-headedness	10/49(20%)	72/235(31%)	0·21	χ^2^	OR 1·29 (0·62 to 2·85)	0·51	OR 1·29 (0·62 to 2·87)	0·51
Fainting or blackouts	2/49(4%)	3/235(1%)	0·21	Fisher’s exact	··	··	··	··
Short term memory loss	Not measured	108/236(46%)	··	··	··	··	··	··
Loss of sense of smell	Not measured	34/236(14%)	··	··	··	··	··	··
Loss of taste	Not measured	33/236(14%)	··	··	··	··	··	··
Fatigue	27/49(55%)	146/231(63%)	0·37	χ^2^	OR 1·92 (1·00 to 3·74)	0·052	OR 1·98 (1·02 to 3·89)	0·044
Dyspnoea-12 score	0·00 (0·00 to 0·25)	3·00 (0·00 to 10–00)	<0·0001	Mann-Whitney U	β 0·70 (0·38 to 1·01)	<0·0001	β 0·66 (0·35 to 0·98)	<0·0001
FACIT V4 Score	Not measured	35·6(11·9)	··	··	··	··	··	··
Anxiety GAD-7 score	1·00 (0·00 to 4·00)	3·00 (0·00 to 8·00)	0·0017	Mann-Whitney U	β 0·64 (0·31 to 0·97)	0·00017	β 0·65 (0·32 to 0·98)	0·00014
Anxiety GAD-7 score >8	3/47(6%)	47/226(21%)	0·034	χ^2^	OR 7·67 (1·91 to 73·83)	0·019	OR 8·01 (1·98 to 76·70)	0·017
Depression PHQ-9 score	1·00 (0·00 to 5·00)	5·00 (2·00 to 11·00)	<0·0001	Mann-Whitney U	β 0·67 (0·34 to 0·99)	<0·0001	β 0·67 (0·35 to 1·00)	<0·0001
Depression PHQ-9 score ≥10	2/47(4%)	68/224(30%)	0·0004	χ^2^	OR 15·21 (3·35 to 223..19)	0·0053	OR 15·3 (3·4 to 224·5)	0·0053
MOCA corrected score	28·0 (27·0 to 29·5)	27·0 (25–0 to 28–0)	0·0020	Mann-Whitney U	β –0·46 (–0·83 to –0·09)	0·015	β –0·49 (–0·87 to –0·12)	0·010
MOCA corrected score <23	1/31(3%)	16/200(8%)	0·48	Fisher’s exact	··	··	··	··
PHOSP-COVID cluster								
Mild	Not applicable	78/226(35%)	··	··	··	··	··	··
Moderate or cognitive	Not applicable	43/226(19%)	··	··	··	··	··	··
Severe	Not applicable	62/226(27%)	··	··	··	··	··	··
Very severe	Not applicable	43/226(19%)	··	··	··	··	··	··
Symptom status at follow-up after hospitalisation								
Recovered	Not applicable	42/236(18%)	··	··	··	··	··	··
Some symptoms at follow-up visit	Not applicable	79/236(33%)	··	··	··	··	··	··
Not recovered after hospitalisation	Not applicable	115/236(49%)	··	··	··	··	··	··
MRI-derived abnormality								
Lung abnormality affecting >5% parenchyma	3/49(6%)	90/259(35%)	0·0001	χ^2^	OR 14·69 (4·00 to 114·57)	0·0008	OR 13·8 (3·8 to 108·2)	0·0011
Cardiac abnormality	12/49(24%)	54/259(21%)	0·70	χ^2^	OR 0·68 (0·33 to 1·45)	0·30	OR 0·69 (0·33 to 1·49)	0·33
Brain abnormality	9/50(18%)	109/218(50%)	<0·0001	χ^2^	OR 3·47 (1·60 to 8·34)	0·0029	OR 3·32 (1·52 to 8·01)	0·0043
Liver abnormality	28/48(58%)	142/236(60%)	0·94	χ^2^	OR 0·69 (0·34 to 1·38)	0·31	OR 0·71 (0·34 to 1·42)	0·34
Kidney abnormality	3/48(6%)	57/246(23%)	0·014	χ^2^	OR 2·36 (0·89 to 8·02)	0·12	OR 2·36 (0·88 to 8·03)	0·12
Abnormality organ count	··	··	<0·0001	Fisher’s exact	··	··	··	··
No organs	12/52(23%)	34/259(13%)	··	··	··	··	··	··
One organ	26/52(50%)	68/259(26%)	··	··	··	··	··	··
Multiorgan dysfunction (≥2 organs)	14/52(27%)	157/259(61%)	<0·0001	χ^2^	OR 2·86 (1·48 to 5·76)	0·0023	OR 2·77 (1·43 to 5·61)	0·0033

Data are n/N (%), mean (SD) for normally distributed continuous variables, or median (IQR) if not normally distributed, unless stated otherwise. CRP=C-reactive protein. eGFR=estimated glomerular filtration rate. FACIT=Functional Assessment of Chronic Illness Therapy. FVC=forced vital capacity. GAD-7=Generalized Anxiety Disorder 7-item scale. KCO=carbon monoxide transfer coefficient. MOCA=Montreal Cognitive Assessment. OR=odds ratio. PHOSP=Post-hospitalisation COVID-19 study. PHQ-9=Patient Health Questionnaire-9. TLCO=transfer factor of the lung for carbon monoxide.

* Inverse probability weighting was used to adjust imaging variables for confounders, which included age, sex, BMI, smoking, hypertension, hypercholesterolaemia, diabetes, and cardiac, brain, liver, lung, and renal comorbidities; ORs were calculated by logistic regression and β coefficients by linear regression.

## Data Availability

The protocol, consent form, definition and derivation of clinical characteristics and outcomes, training materials, regulatory documents, requests for data access and other relevant study materials are available online at https://www.phosp.org.

## Research in context

### Evidence before this study

A systematic search of PubMed and Embase databases for studies that included multiorgan imaging of lungs, heart, brain, liver, and kidneys published between Jan 1, 2021, and Dec 31, 2022, without language restrictions was conducted. Search terms related to COVID-19 (“COVID-19”, “COVID-2019”, “SARS-CoV-2”, “2019-nCoV”, “2019-SARSCoV-2”), hospitalisation (“hospital”), long-term follow-up (“follow-up”, “long term”, “sequelae”, “long Covid”), multiorgan (“multiorgan”, “multi-organ”) and MRI (“magnetic resonance imaging”, “MRI”) were used. We found only one comprehensive multiorgan MRI study covering five major organs, which was conducted by the C-MORE investigators (n=58); two studies (UK n=159; Hamburg n=443) evaluated three or four organs on MRI and investigated predominantly non-hospitalised patients. Single-organ MRI studies were common, but did not examine the cumulative burden of multimorbidity or multiorgan dysfunction on patient recovery following severe acute infection. At the time of the search, there were no multiorgan MRI follow-up studies in post-hospitalised patients with COVID-19 at the 5–6 month follow-up timepoint. One follow-up MRI study of non-hospitalised patients with COVID-19 in the UK (n=534) used a limited non-contrast (non-clinical) protocol and evaluated longitudinal organ health in patients following mild infection. This study reported a prevalence of 59% for single-organ abnormalities and 27% for multiorgan abnormalities. None of these studies evaluated the association of multiorgan health (lungs, heart, brain, liver, or kidneys) with recently identified post-COVID-19 symptom clusters relevant to post-hospitalised patients (eg, PHOSP-COVID recovery clusters based on physical, mental, and cognitive impairment). We also found no study investigating the association of systemic inflammation and multiorgan (combination of up to five organs) health among post-hospitalised patients.

### Added value of this study

In one of the largest prospective, multicentre, multiorgan MRI follow-up studies of post-hospitalised patients with COVID-19 and contemporary non-COVID-19 controls, we report several novel findings: (1) 5 months after hospitalisation with COVID-19, nearly one in three patients had evidence of multiorgan abnormalities on MRI; (2) patients had an excess burden of lung, brain, and renal abnormalities; (3) both cardiac and liver MRI abnormalities were similar between patients and our non-COVID-19 control population; (4) older age with comorbidities, more severe acute infection, and prolonged hospitalisation were linked to multiorgan abnormalities on MRI; and (5) patients with MRI evidence of multiorgan injury were more likely to have severe or very severe persistent physical and mental health impairment.

### Implications of all the available evidence

Our research builds on the expanding body of evidence indicating that patients previously hospitalised due to COVID-19 face a heightened risk of multiorgan damage, during both the acute phase and the post-acute period. Imaging studies of post-hospitalised patients suggest a higher likelihood of sustained injuries to the lungs, brain, and kidneys, while the heart and liver appear to be relatively resilient. These insights emphasise the urgency of multidisciplinary health care, particularly aimed at screening and addressing both pulmonary and extrapulmonary complications, including neurocognitive and renal health. The potential benefits of advanced multiorgan imaging in determining surveillance frequency and guiding therapeutic interventions remain an active area of exploration. The question of whether this approach can effectively assess individual vulnerability to subsequent complications is still open. However, the prevailing evidence strongly advocates for detailed follow-ups, especially for older patients, those with a severe onset of infection, and those with multiple underlying conditions. Furthermore, patients with ongoing severe physical and mental health challenges after hospitalisation would probably benefit from a thorough multisystem health evaluation.
