# Supplementary material for: Multiorgan MRI findings after hospitalisation with COVID-19 in the UK (C-MORE): a prospective, multicentre, observational cohort study
Source: Lancet Respir Med. Author manuscript; Available in PMC 2023 Nov 1. (PMC7615263; doi:10.1016/S2213-2600(23)00262-X)
Supplement: Supplementary appendix [file EMS188591-supplement-Supplementary_appendix.pdf]

THE LANCET
Respiratory Medicine

Supplementary appendix

This appendix formed part of the original submission and has been peer reviewed. We post it as supplied by the authors.

Supplement to: The C-MORE/PHOSP-COVID Collaborative Group. Multiorgan MRI findings after hospitalisation with COVID-19 in the UK (C-MORE): a prospective, multicentre, observational cohort study. *Lancet Respir Med* 2023; published online Sept 22. https://doi.org/10.1016/S2213-2600(23)00262-X.

Table of Contents

C-MORE/PHOSP-COVID Collaborative group 1

Abbrevations 11

Methods 12

Inclusion Criteria 12

Exclusion Criteria 12

Definitions 12

Multiorgan magnetic resonance imaging (MRI) 12

MRI acquisition and analyses 13

Lung MRI 13
Lung MRI Analysis 13
Cardiac MRI 14
Cardiac MRI image analysis 14
Brain MRI 15
Brain MRI analysis 16
Liver MRI 16
Liver MRI Analysis 17
Kidney MRI 17
Kidney MRI Analysis 18
Definition of multiorgan abnormalities 18

Spirometry 20

Patient related outcomes Measures 20

Patient health questionnaire (PHQ-9) 20
General Anxiety Disorder Questionnaire (GAD-7) 20
Montreal Cognitive Assessment (MOCA) Tool 20
Dyspnoea-12 questionnaire 21
FACIT-F 21
PCL-5 21
Symptom and recovery questionnaire 21
PHOSP-COVID Symptom clusters 21
Tables Legend 24

Supplementary Table 1. A) Comparison of lung MRI in patients vs controls; B) Clinical characteristics of patients with and without lung MRI abnormalities; C) Clinical characteristics of patients with and without lung MRI abnormalities – excluding pre-existing respiratory conditions. ***24***

Part A: Comparison of lung health between patients versus controls 24
Part B: Clinical characteristics of patients with and without lung MRI abnormalities 25
Part C: Clinical characteristics of patients with and without lung MRI abnormalities after excluding those with pre-existing respiratory conditions 26

Supplementary Table. 2. Comparison of quantitative multiorgan MRI metrics across patients with and without an abnormal C-reactive protein at follow visit (median 5 months). ***28***

Supplementary Table 3. A) Comparison of heart MRI in patients vs controls; B) Clinical characteristics of patients with and without heart MRI abnormalities; C) Clinical characteristics of patients with and without heart MRI abnormalities after excluding pre-existing cardiac conditions. ***29***

Part B: Clinical characteristics of patients with and without cardiac MRI abnormalities 30

Part C: Clinical characteristics of patients with and without cardiac MRI abnormalities after excluding pre-existing cardiac conditions 31

Supplementary Table 4. A) Comparison of brain MRI in patients vs controls; B) Clinical characteristics of patients with and without brain MRI abnormalities; C) Clinical characteristics of patients with and without brain MRI abnormalities after excluding pre-existing neurological conditions..... 33

Part A: Comparison of brain health between patients and controls 33

Part B: Clinical characteristics of patients with and without brain MRI abnormalities 34

Part C: Clinical characteristics of patients with and without brain MRI abnormalities after excluding pre-existing neurological conditions..... 36

Supplementary Table 5. A) Comparison of liver MRI in patients vs controls; B) Clinical characteristics of patients with and without liver MRI abnormalities; C) Clinical characteristics of patients with and without liver MRI abnormalities after excluding pre-existing liver conditions. 38

Part A: Comparison of liver health between patients and controls 38

Part B: Clinical characteristics of patients with and without liver MRI abnormalities 38

Part C: Clinical characteristics of patients with and without liver MRI abnormalities after excluding pre-existing liver conditions 39

Supplementary Table 6. A) Comparison of Renal MRI in patients vs controls; B) Clinical characteristics of patients with and without renal abnormalities c) Clinical characteristics of patients with and without renal abnormalities after excluding pre-existing renal conditions . 41

Part A: Comparison of renal health between patients vs controls 41

Part B: Clinical characteristics of patients with and without renal MRI abnormalities..... 41

Part C: Clinical characteristics of patients with and without renal abnormalities after excluding pre-existing renal conditions 42

Supplementary Table 7. Post-hoc comparison of clinical characteristics and MRI organ abnormalities between post-hospitalised COVID-19 patients and controls matched for age, sex and obesity. 45

References 47

C-MORE/PHOSP-COVID Collaborative group

Core Management Group

Chief Investigator C E Brightling, *Members* R A Evans (Lead Co-I), L V Wain (Lead Co-I), J D Chalmers, V C Harris, L P Ho, A Horsley, M Marks, K Poinasamy, B Raman, A Shikotra, A Singapuri

PHOSP-COVID Study Central Coordinating Team

C E Brightling (Chief Investigator), R A Evans (*Lead Co-I*), L V Wain (*Lead Co-I*), R Dowling, C Edwardson, O Elneima, S Finney, N J Greening, B Hargadon, V C Harris, L Houchen--Wolloff, O C Leavy, H J C McAuley, C Overton, T Plekhanova, R M Saunders, M Sereno, A Singapuri, A Shikotra, C Taylor, S Terry, C Tong, B Zhao

Steering Committee

Co-chairs D Lomas, E Sapey, *Institution representatives* C Berry, C E Bolton, N Brunskill, E R Chilvers, R Djukanovic, Y Ellis, D Forton, N French, J George, N A Hanley, N Hart, L McGarvey, N Maskell, H McShane, M Parkes, D Peckham, P Pfeffer, A Sayer, A Sheikh, A A R Thompson, N Williams and core management group representation

Executive Board

Chair C E Brightling, representation from the core management group, each working group and platforms

Platforms

Bioresource

W Greenhalf (*Co-Lead*), M G Semple (*Co-Lead*), M Ashworth, H E Hardwick, L Lavelle-Langham, W Reynolds, M Sereno, R M Saunders, A Singapuri, V Shaw, A Shikotra, B Venson, L V Wain

Data Hub

A B Docherty (*Co-Lead*), E M Harrison (*Co-Lead*), A Sheikh (*Co-Lead*), J K Baillie, C E Brightling, L Daines, R Free, R A Evans, S Kerr, O C Leavy, N I Lone, H J C McAuley, R Pius, J Quint, M Richardson, M Sereno, M Thorpe, L V Wain

Imaging Alliance

M Halling-Brown (*Co-Lead*), F Gleeson (*Co-Lead*), J Jacob (*Co-Lead*), S Neubauer (*Co-Lead*) B Raman (*Co-Lead*) S Siddiqui (*Co-Lead*) J M Wild (*Co-Lead*), S Aslani, P Jezzard, H Lamlum, W Lilaonitkul, E Tunnicliffe, J Willoughby

C-MORE Investigators (Part of Imaging Alliance)

C-MORE *(Co-lead)* S Neubauer and B Raman (*Co-lead*), M Beggs , M P Cassar, A Chiribiri, E Cox, D J Cuthbertson, V M Ferreira, L Finnigan, S Francis, M Halling-Brown, G J Kemp, H Lamlum, E Lukaschuk, C Manisty, G P McCann , K McGlynn , R Menke , C A Miller , A J Moss , C Nikolaidou , C O'Brien , D P O'Regan , S Piechnik , S Plein, I

Propescu, A A Samat, L Saunders, R Steeds, T Treibel, E M Tunnicliffe, J Weir McCall, J M Wild, C Xie, G Ogbole, Z B Sanders, M Webster, S Smith, Miller K, C McCracken, T E Nichols, P Jezzard.

Omics

L V Wain (*Co-Lead)*, J K Baillie (*Co-Lead*), H Baxendale, C E Brightling, M Brown, J D Chalmers, R A Evans, B Gooptu, W Greenhalf, H E Hardwick, R G Jenkins, D Jones, I Koychev, C Langenberg, A Lawrie, P L Molyneaux, A Shikotra, J Pearl, M Ralser, N Sattar, R M Saunders, J T Scott, T Shaw, D Thomas, D Wilkinson

Working Groups

Airways

L G Heaney (*Co-Lead*), A De Soyza (*Co-Lead*), D Adeloye, C E Brightling, J S Brown, J Busby, J D Chalmers, C Echevarria, L Daines, O Elneima, RA Evans, J Hurst, P Novotny, P Pfeffer, K Poinasamy, J Quint, I Rudan, E Sapey, M Shankar-Hari, A Sheikh, S Siddiqui, S Walker, B Zheng

Brain

J R Geddes (*Lead*), M Hotopf *(Co-Lead),* K Abel, R Ahmed, L Allan, C Armour, D Baguley, D Baldwin, C Ballard, K Bhui, G Breen, M Broome, T Brugha, E Bullmore, D Burn, F Callard, J Cavanagh, T Chalder, D Clark, A David, B Deakin, H Dobson, B Elliott, J Evans, R Francis, E Guthrie, P Harrison, M Henderson, A Hosseini, N Huneke, M Husain, T Jackson, I Jones, T Kabir, P Kitterick, A Korszun, I Koychev, J Kwan, A Lingford-Hughes, P Mansoori, H McAllister-Williams, K McIvor, L Milligan, R Morriss, E Mukaetova-Ladinska, K Munro, A Nevado-Holgado, T Nicholson, S Paddick, C Pariante, J Pimm, K Saunders, M Sharpe, G Simons, R Upthegrove, S Wessely

Cardiac

G P McCann (*Lead*), S Amoils, C Antoniades, A Banerjee, R Bell, A Bularga, C Berry, P Chowienczyk, J P Greenwood, A D Hughes, K Khunti, L Kingham, C Lawson, K Mangion, N L Mills, A J Moss, S Neubauer, B Raman, A N Sattar, C L Sudlow, M Toshner,

Immunology

P J M Openshaw (*Lead*), D Altmann, J K Baillie, R Batterham, H Baxendale, N Bishop, C E Brightling, P C Calder, R A Evans, J L Heeney, T Hussell, P Klenerman, J M Lord, P Moss, S L Rowland-Jones, W Schwaeble, M G Semple, R S Thwaites, L Turtle, L V Wain, S Walmsley, D Wraith

Intensive Care

M J Rowland (*Lead*), A Rostron (*Co-Lead*), J K Baillie, B Connolly, A B Docherty, N I Lone, D F McAuley, D Parekh, A Rostron, J Simpson, C Summers

Lung Fibrosis

R G Jenkins (*Co-Lead*), J Porter (*Co-Lead*), R J Allen, R Aul, J K Baillie, S Barratt, P Beirne, J Blaikley, R C Chambers, N Chaudhuri, C Coleman, E Denneny, L Fabbri, P M George, M Gibbons, F Gleeson, B Gooptu, B Guillen Guio, I Hall, N A Hanley, L P Ho, E Hufton, J Jacob, I Jarrold, G Jenkins, S Johnson, M G Jones, S Jones, F Khan, P Mehta, J Mitchell, P L Molyneaux, J E Pearl, K Piper Hanley, K Poinasamy, J Quint, D Parekh, P Rivera-Ortega,

L C Saunders, M G Semple, J Simpson, D Smith, M Spears, L G Spencer, S Stanel, I Stewart, A A R Thompson, D Thickett, R Thwaites, L V Wain, S Walker, S Walsh, J M Wild, D G Wootton, L Wright

Metabolic

S Heller (*Co-Lead*), M J Davies (*Co-Lead*), H Atkins, S Bain, J Dennis, K Ismail, D Johnston, P Kar, K Khunti, C Langenberg, P McArdle, A McGovern, T Peto, J Petrie, E Robertson, N Sattar, K Shah, J Valabhji, B Young

Pulmonary and Systematic Vasculature

L S Howard (*Co-Lead*), Mark Toshner (*Co-Lead*), C Berry, P Chowienczyk, D Lasserson, A Lawrie, O C Leavy, J Mitchell, J Newman, L Price, J Quint, A Reddy, J Rossdale, N Sattar, C Sudlow, A A R Thompson, J M Wild, M Wilkins

Rehabilitation, Sarcopenia and Fatigue

S J Singh (*Co-Lead*), W D-C Man (*Co-Lead*), J M Lord (*Co-Lead*), N J Greening (*Co-Lead*), T Chalder (*Co-Lead*), J T Scott (*Co-Lead*), N Armstrong, E Baldry, M Baldwin, N Basu, M Beadsworth, L Bishop, C E Bolton, A Briggs, M Buch, G Carson, J Cavanagh, H Chinoy, E Daynes, S Defres, R A Evans, P Greenhaff, S Greenwood, M Harvie, M Husain, S MacDonald, A McArdle, H J C McAuley, A McMahon, M McNarry, G Mills, C Nolan, K O'Donnell, D Parekh, Pimm, J Sargent, L Sigfrid, M Steiner, D Stensel, A L Tan, J Whitney, D Wilkinson, D Wilson, M Witham, D G Wootton, T Yates

Renal

D Thomas (*Lead*), N Brunskill (*Co-Lead*), S Francis (*Co-Lead*), S Greenwood (*Co-Lead*), C Laing (*Co-Lead*), K Bramham, P Chowdhury, A Frankel, L Lightstone, S McAdoo, K McCafferty, M Ostermann, N Selby, C Sharpe, M Willicombe

Local Clinical Centre PHOSP-COVID trial staff

(listed in alphabetical order)

Airedale NHS Foundation Trust

A Shaw (PI), L Armstrong, B Hairsine, H Henson, C Kurasz, L Shenton

Aneurin Bevan University Health Board

S Fairbairn (PI), A Dell, N Hawkings, J Haworth, M Hoare, A Lucey, V Lewis, G Mallison, H Nassa, C Pennington, A Price, C Price, A Storrie, G Willis, S Young

Barts Health NHS Trust & Queen Mary University of London

P Pfeffer (PI), K Chong-James, C David, W Y James, A Martineau, O Zongo, Charlotte Manisty, T Treibel

Barnsley Hospital NHS Foundation Trust

A Sanderson (PI)

Belfast Health and Social Care Trust & Queen's University Belfast
L G Heaney (PI), C Armour, V Brown, T Craig, S Drain, B King, N Magee, D McAulay, E Major, L McGarvey, J McGinness, R Stone

Betsi Cadwaladr University Health Board
A Haggar (PI), A Bolger, F Davies, J Lewis, A Lloyd, R Manley, E McIvor, D Menzies, K Roberts, W Saxon, D Southern, C Subbe, V Whitehead

Borders General Hospital, NHS Borders

H El-Taweel (PI), J Dawson, L Robinson

Bradford Teaching Hospitals NHS Foundation Trust

D Saralaya (PI), L Brear, K Regan, K Storton

Cambridge University Hospitals NHS Foundation Trust, NIHR Cambridge Clinical Research Facility & University of Cambridge
J Fuld (PI), A Bermperi, I Cruz, K Dempsey, A Elmer, H Jones, S Jose, S Marciniak, M Parkes, C Ribeiro, J Taylor, M Toshner, L Watson, J Worsley, J Weir McCall

Cardiff and Vale University Health Board

R Sabit (PI), L Broad, A Buttress, T Evans, M Haynes, L Jones, L Knibbs, A McQueen, C Oliver, K Paradowski, J Williams

Chesterfield Royal Hospital NHS Trust

E Harris (PI), C Sampson

Cwm Taf Morgannwg University Health Board

C Lynch (PI), E Davies, C Evenden , A Hancock, K Hancock, M Rees , L Roche, N Stroud, T Thomas-Woods

East Cheshire NHS Trust

M Babores (PI), J Bradley-Potts, M Holland, N Keenan, S Shashaa, H Wassall

East Kent Hospitals University NHS Foundation Trust

E Beranova (PI), H Weston (PI), T Cosier, L Austin, J Deery, T Hazelton, C Price, H Ramos, R Solly, S Turney

Gateshead NHS Trust

L Pearce (PI), W McCormack, S Pugmire, W Stoker, A Wilson

Guy’s and St Thomas’ NHS Foundation Trust
N Hart (PI), LA Aguilar Jimenez, G Arbane, S Betts, K Bisnauthsing, A Dewar, P Chowdhury, A Dewar, G Kaltsakas, H Kerslake, MM Magtoto, P Marino, LM Martinez, M Ostermann, J Rossdale, TS Solano, E Wynn

Hampshire Hospitals NHS Foundation Trust
N Williams (PI), W Storrar (PI), M Alvarez Corral, A Arias, E Bevan, D Griffin, J Martin, J Owen, S Payne, A Prabhu, A Reed, C Wrey Brown

Harrogate and District NHD Foundation Trust

C Lawson (PI), T Burdett, J Featherstone, A Layton, C Mills, L Stephenson,

Hull University Teaching Hospitals NHS Trust & University of Hull
N Easom (PI), P Atkin, K Brindle, M G Crooks, K Drury, R Flockton, L Holdsworth, A Richards, D L Sykes, S Thackray-Nocera, C Wright

Hywel Dda University Health Board

K E Lewis (PI), A Mohamed (PI), G Ross (PI), S Coetzee, K Davies, R Hughes, R Loosley, L O'Brien, Z Omar, H McGuinness, E Perkins, J Phipps, A Taylor, H Tench, R Wolf-Roberts

Imperial College Healthcare NHS Trust & Imperial College London
L S Howard (PI), O Kon (PI), D C Thomas (PI), S Anifowose, L Burden, E Calvelo, B Card, C Carr, E R Chilvers, D Copeland, P Cullinan, P Daly, L Evison, T Fayzan, H Gordon, S Haq, R G Jenkins, C King, K March, M Mariveles, L McLeavey, N Mohamed, S Moriera, U Munawar, J Nunag, U Nwanguma, L Orriss- Dib, A Ross, M Roy, E Russell, K Samuel, J Schronce, N Simpson, L Tarusan, C Wood, N Yasmin, D P O'Regan

Kettering General Hospital NHS Trust

R Reddy (PI), A-M, Guerdette, M Hewitt, K Warwick, S White

King's College Hospital NHS Foundation Trust & Kings College London
A M Shah (PI), C J Jolley (PI), O Adeyemi, R Adrego, H Assefa-Kebede, J Breeze, M Brown, S Byrne, T Chalder, P Dulawan, N Hart, A Hayday, A Hoare, A Knighton, M Malim, S Patale, I Peralta, N Powell, A Ramos, K Shevket, F Speranza, A Te

Leeds Teaching Hospitals & University of Leeds
P Beirne (PI), A Ashworth, J Clarke, C Coupland, M Dalton, E Wade, C Favager, J Greenwood, J Glossop, L Hall, T Hardy, A Humphries, J Murira, D Peckham, S Plein, J Rangeley, G Saalmink, A L Tan, B Whittam, N Window, J Woods, S Plein.

Lewisham & Greenwich NHS Trust

G Coakley (PI)

Liverpool University Hospitals NHS Foundation Trust & University of Liverpool

D G Wootton (PI), L Turtle (PI), L Allerton, AM All, M Beadsworth, A Berridge, J Brown, S Cooper, A Cross, S Defres, S L Dobson, J Earley, N French, W Greenhalf, H E Hardwick, K Hainey, J Hawkes, V Highett, S Kaprowska, AL Key, L Lavelle-Langham, N Lewis-Burke, G Madzamba, F Malein, S Marsh, C Mears, L Melling, M J Noonan,

L Poll, J Pratt, E Richardson, A Rowe, M G Semple, V Shaw, K A Tripp, L O Wajero, S A Williams-Howard, J Wyles, G J Kemp, D J Cuthbertson.

London North West University Healthcare NHS Trust
S N Diwanji (PI), P Papineni (PI), S Gurram, S Quaid, G F Tiongson, E Watson

Manchester University NHS Foundation Trust & University of Manchester
B Al-Sheklly (PI), A Horsley (PI), C Avram, J Blaikely, M Buch, N Choudhury, D Faluyi, T Felton, T Gorsuch, N A Hanley, T Hussell, Z Kausar, N Odell, R Osbourne, K Piper Hanley, K Radhakrishnan, S Stockdale, C A Miller.

Newcastle upon Tyne Hospitals NHS Foundation Trust & University of Newcastle
A De Soyza (PI), C Echevarria (PI), A Ayoub, J Brown, G Burns, G Davies, H Fisher, C Francis, A Greenhalgh, P Hogarth, J Hughes, K Jiwa, G Jones, G MacGowan, D Price, A Sayer, J Simpson, H Tedd, S Thomas, S West, M Witham, S Wright, A Young

NHS Dumfries and Galloway
M J McMahon (PI), P Neill

NHS Greater Glasgow and Clyde Health Board & University of Glasgow
D Anderson (PI), H Bayes (PI), C Berry (PI), D Grieve (PI), I B McInnes (PI), N Basu, A Brown, A Dougherty, K Fallon, L Gilmour, K Mangion, A Morrow, K Scott, R Sykes

NHS Highland
E K Sage (PI), F Barrett, A Donaldson

NHS Lanarkshire
M Patel (PI), D Bell, A Brown, M Brown, R Hamil, K Leitch, L Macliver, J Quigley, A Smith, B Welsh

NHS Lothian & University of Edinburgh

G Choudhury (PI), J K Baillie, S Clohisey, A Deans, A B Docherty, J Furniss, E M Harrison, S Kelly, N I Lone, A Sheikh

NHS Tayside & University of Dundee
J D Chalmers (PI), D Connell, A Elliott, C Deas, J George, S Mohammed, J Rowland, A R Solstice, D Sutherland, C J Tee

North Bristol NHS Trust & University of Bristol

N Maskell (PI), D Arnold, S Barrett, H Adamali, A Dipper, S Dunn, A Morley, L Morrison, L Stadon, S Waterson, H Welch

North Middlesex Hospital NHS Trust
B Jayaraman (PI), T Light

Nottingham University Hospitals NHS Trust & University of Nottingham

C E Bolton (PI), P Almeida, J Bonnington, M Chrystal, C Dupont, P Greenhaff, A Gupta, L Howard, W Jang, S Linford, L Matthews, R Needham, A Nikolaidis, S Prosper, K Shaw, A K Thomas, S Francis,

Oxford University Hospitals NHS Foundation Trust & University of Oxford
L P Ho (PI), N M Rahman (PI), M Ainsworth, A Alamoudi, A Bates, A Bloss, A Burns, P Carter, J Chen, F Conneh, T Dong, R I Evans, E Fraser, X Fu, J R Geddes, F Gleeson, P Harrison, M Havinden-Williams, P Jezzard, N Kanellakis, I Koychev, P Kurupati, X Li, H McShane, C Megson, K Motohashi, S Neubauer, D Nicoll, G Ogg, E Pacpaco, M Pavlides, Y Peng, N Petousi, N Rahman, B Raman, M J Rowland, K Saunders, M Sharpe, N Talbot, E Tunnicliffe

Royal Brompton and Harefield Clinical Group, Guy's and St Thomas' NHS Foundation Trust.

W D-C Man (PI), B Patel (PI), R E Barker, D Cristiano, N Dormand, M Gummadi, S Kon, K Liyanage, C M Nolan, S Patel, O Polgar, P Shah, S J Singh, J A Walsh

Royal Free London NHS Foundation Trust
J Hurst (PI), H Jarvis (PI), S Mandal (PI), S Ahmad, S Brill, L Lim, D Matila, O Olaosebikan, C Singh

Royal Papworth Hospital NHS Foundation Trust

M Toshner (PI), H Baxendale, L Garner, C Johnson, J Mackie, A Michael, J Pack, K Paques, H Parfrey, J Parmar

Salford Royal NHS Foundation Trust
N Diar Bakerly (PI), P Dark, D Evans, E Hardy, A Harvey, D Holgate, S Knight, N Mairs, N Majeed, L McMorrow, J Oxton, J Pendlebury, C Summersgill, R Ugwuoke, S Whittaker

Salisbury NHS Foundation Trust

W Matimba-Mupaya (PI), S Strong-Sheldrake

Sheffield Teaching NHS Foundation Trust & University of Sheffield

S L Rowland-Jones (PI), A A R Thompson (Co PI), J Bagshaw, M Begum, K Birchall, R Butcher, H Carborn, F Chan, K Chapman, Y Cheng, L Chetham, C Clark, Z Coburn, J Cole, M Dixon, A Fairman, J Finnigan, H Foot, D Foote, A Ford, R Gregory, K Harrington, L Haslam, L Hesselden, J Hockridge, A Holbourn, B Holroyd-Hind, L Holt, A Howell, E Hurditch, F Ilyas, C Jarman, A Lawrie, E Lee, J-H Lee, R Lenagh, A Lye, I Macharia, M Marshall, A Mbuyisa, J McNeill, S Megson, J Meiring, L Milner, S Misra, H Newell, T Newman, C Norman, L Nwafor, D Pattenadk, M Plowright, J Porter, P Ravencroft, C Roddis, J Rodger, P Saunders, J Sidebottom, J Smith, L Smith, N Steele, G Stephens, R Stimpson, B Thamu, N Tinker, K Turner, H Turton, P Wade, S Walker, J Watson, I Wilson, A Zawia, J M Wild

St George's University Hospitals NHS Foundation Trust
R Aul (PI), M Ali, A Dunleavy (PI), D Forton, N Msimanga, M Mencias, T Samakomva, S Siddique, J Teixeira, V Tavoukjian

Sherwood Forest Hospitals NHS Foundation Trust

J Hutchinson (PI), L Allsop, K Bennett, P Buckley, M Flynn, M Gill, C Goodwin, M Greatorex, H Gregory, C Heeley, L Holloway, M Holmes, J Kirk, W Lovegrove, TA Sewell, S Shelton, D Sissons, K Slack, S Smith, D Sowter, S Turner, V Whitworth, I Wynter

Shropshire Community Health NHS Trust
L Warburton (PI), S Painter, J Tomlinson

Somerset NHS Foundation Trust
C Vickers (PI), T Wainwright, D Redwood, J Tilley, S Palmer

Swansea Bay University Health Board
G A Davies (PI), L Connor, A Cook, T Rees, F Thaivalappil, C Thomas

Tameside and Glossop Integrated Care NHS Foundation

A Butt (PI), M Coulding, H Jones, S Kilroy, J McCormick, J McIntosh, H Savill, V Turner, J Vere

The Great Western Hospital Foundation Trust

E Fraile (PI), J Ugoji

The Hillingdon Hospitals NHS Foundation Trust
S S Kon (PI), H Lota, G Landers, M Nasseri, S Portukhay

The Rotherham NHS Foundation Trust

A Hormis (PI), A Daniels, J Ingham, L Zeidan

United Lincolnshire Hospitals NHS Trust
M Chablani (PI), L Osborne

University College London Hospital & University College London
M Marks (PI), J S Brown (PI), N Ahwireng, B Bang, D Basire, R C Chambers, A Checkley, R Evans, M Heightman, T Hillman, J Hurst, J Jacob, S Janes, R Jastrub, M Lipman, S Logan, D Lomas, M Merida Morillas, H Plant, J C Porter, K Roy, E Wall, T Treibel.

University Hospital Birmingham NHS Foundation Trust & University of Birmingham
D Parekh (PI), N Ahmad Haider, C Atkin, R Baggott, M Bates, A Botkai, A Casey, B Cooper, J Dasgin, K Draxlbauer, N Gautam, J Hazeldine, T Hiwot, S Holden, K Isaacs, T Jackson, S Johnson, V Kamwa, D Lewis,
J M Lord, S Madathil, C McGhee, K Mcgee, A Neal, A Newton Cox, J Nyaboko, D Parekh, Z Peterkin, H Qureshi, L Ratcliffe, E Sapey, J Short, T Soulsby, J Stockley, Z Suleiman, T Thompson, M Ventura, S Walder, C Welch, D Wilson, S Yasmin, K P Yip, R Steeds.

University Hospitals of Derby and Burton

P Beckett (PI) C Dickens, U Nanda

University Hospitals of Leicester NHS Trust & University of Leicester

C E Brightling (CI), R A Evans (PI), M Aljaroof, N Armstrong, H Arnold, H Aung, M Bakali, M Bakau, M Baldwin, M Bingham, M Bourne, C Bourne, N Brunskill, P Cairns, L Carr, A Charalambou, C Christie, M J Davies, S Diver,

S Edwards, C Edwardson, O Elneima, H Evans, J Finch, S Glover, N Goodman, B Gootpu, N J Greening, K Hadley, P Haldar, B Hargadon, V C Harris, L Houchen-Wolloff, W Ibrahim, L Ingram, K Khunti, A Lea, D Lee, G P McCann, H J C McAuley, P McCourt, T Mcnally, G Mills, A Moss, W Monteiro, M Pareek, S Parker, A Rowland, A Prickett, I N Qureshi, R Russell, M Sereno, A Shikotra, S Siddiqui, A Singapuri, S J Singh, J Skeemer, M Soares, E Stringer, T Thornton, M Tobin, L V Wain, T J C Ward, F Woodhead, T Yates, A Yousuf

University Hospital Southampton NHS Foundation Trust & University of Southampton
M G Jones (PI), C Childs, R Djukanovic, S Fletcher, M Harvey, E Marouzet, B Marshall, R Samuel, T Sass, T Wallis, H Wheeler

Whittington Health NHS
R Dharmagunawardena (PI), E Bright, P Crisp, M Stern

Wirral University Teaching Hospital

A Wight (PI), L Bailey, A Reddington

Wrightington Wigan and Leigh NHS trust

A Ashish (PI), J Cooper, E Robinson

Yeovil District Hospital NHS Foundation Trust

A Broadley (PI)

York & Scarborough NHS Foundation Trust

K Howard (PI), L Barman, C Brookes, K Elliott. L Griffiths, Z Guy, D Ionita, H Redfearn, C Sarginson A Turnbull

Health and Care Research Wales

Y Ellis

London School of Hygiene & Tropical Medicine (LSHTM)
M Marks, A Briggs

NIHR Office for Clinical Research Infrastructure

K Holmes

Patient Public Involvement Leads

Asthma UK and British Lung Foundation Partnership - K Poinasamy, S Walker

Royal Surrey NHS Foundation Trust
M Halling-Brown

South London and Maudsley NHS Foundation Trust & Kings College London

G Breen, M Hotopf

Swansea University & Swansea Welsh Network

K Lewis, N Williams

Abbrevations
COVID-19, coronavirus disease
PHOSP-COVID, Post-hospitalisation COVID-19 follow-up study
WHO, World health organisation
NIV, Non-invasive ventilation
ECMO, Extra-corporeal membrane oxygenation
AKI, Acute kidney injury
AST, Aspartate aminotransferase
MRI, Magnetic resonance imaging
TR, Repetition Time
TE, Echo time
TI, Inversion time
GRE. Gradient echo
sPGR Spoiled gradient echo
HASTE, Half-Fourier acquisition single-shot turbo spin-echo
SENSE sensitivity encoding
T2 FLAIR fluid attenuated inversion recovery
MPRAGE, Magnetization-Prepared Rapid Acquisition Gradient Echo
EPI, echo planar imaging
ASL, Arterial spin labelling
MD, Mean diffusivity
QSM, quantitative susceptibility mapping
SSFP, steady state free precession imaging
ShMOLLI, Shortened modified Look Locker Inverstion recovery
MOLLI Modified Look Locker Inverstion recovery
LV, Left ventricle
RV, Right ventricle
EDV, End diastolic volume
ESV, End systolic volume
SV, Stroke volume
EF, Ejection fraction
LGE, Late gadolinium enhancement
ECV, Extracellular volume
SD, Standard deviation
PDFF, Proton density fat fraction
IDEAL, Iterative decomposition of water and fat with echo asymmetry and least-squares estimation
DIXON Method of GRE imaging
LIC, Liver iron concentration
GRAPPA, GeneRalized Autocalibrating Partial Parallel Acquisition
CNN, Convolutional neural network
TKV, Total kidney volume
ROI, Region of interest
FEV1, Forced expiratory volume in 1 second
FVC, Forced vital capacity

Methods

Inclusion Criteria

Patients with moderate to severe COVID-19 (coronavirus disease) who were enrolled in the PHOSP-COVID study (details of inclusion criteria published elsewhere[1]) were eligible for this study. Controls were non-hospitalised subjects (invited from the community) without symptoms or signs of a respiratory tract infection of coronavirus disease, who were screened for SARS-CoV-2 core antibodies and tested negative.

Exclusion Criteria

Subjects with contraindications to magnetic resonance imaging (metal implant in body, known claustrophobia, pacemakers, contrast allergy) and end stage renal failure were excluded from the study.

Definitions

Moderate to severe SARS-CoV-2 infection: Severity of acute illness on admission was defined by the World Health Organisation (WHO) progression scale.[2] All patients with clinical signs of pneumonia such as respiratory rate > 30 breaths/min; or severe respiratory distress; or SpO2 < 90% (on room air) and requiring hospital admission for more than 48 hours were assessed to have moderate to severe COVID-19.[3]

Patient State	Descriptor	Score
Uninfected	Uninfected; no viral RNA detected	0
Ambulatory mild disease	Asymptomatic; viral RNA detected	1
	Symptomatic; independent	2
	Symptomatic; assistance needed	3
Hospitalised: moderate disease	Hospitalised; no oxygen therapy*	4
	Hospitalised; oxygen by mask or nasal prongs	5
Hospitalised: severe diseases	Hospitalised; oxygen by NIV or high flow	6
	Intubation and mechanical ventilation, pO_2/FiO_2 ≥150 or SpO_2/FiO_2 ≥200	7
	Mechanical ventilation pO_2/FIO_2 <150 (SpO_2/FiO_2 <200) or vasopressors	8
	Mechanical ventilation pO_2/FiO_2 <150 and vasopressors, dialysis, or ECMO	9
Dead	Dead	10

Adapted from WHO Working group on the Clinical Characterisation and Management of COVID-19. Lancet Infect Dis 20.8 (2020): e192-e197.[2]

Severity of disease (or clinical response) during admission: Patient response in hospital was defined by the WHO clinical progression scale.[4] A score 0 was given to an uninfected individual, 1 to an ambulatory patient without limitations of activities, 2 where there was limitations of activities, 3 were patient was symptomatic and assistance was needed, 4 for hospitalised individuals with no oxygen therapy, 5 for hospitalised individuals receiving O_2 supplementation via simple face mask or nasal prongs, 6 for administration of non-invasive ventilation (NIV) or high flow O_2, 7 for being intubated and on mechanical ventilation, 8 for requiring ventilation, and 9 for additional organ support such as renal replacement therapy and extracorporeal membrane oxygenation (ECMO), and 10 for death.[4]

Multiorgan magnetic resonance imaging (MRI)

Scans were carried out during a single scan session. Details of the scanners at each sites are listed below.

Site	Scanner
Oxford	3T, Siemens, Prisma

Leicester	3T, Siemens, Skyra
Cambridge	3T, Siemens, Prisma
Leeds	3T, Siemens, Prisma
Nottingham	3T, Philips, Ingenia
Sheffield	3T, Philips, Ingenia
Manchester	3T, Siemens, Magnetom Vida
Birmingham	3T, Siemens, Skyra
Imperial College, London	3T, Siemens, Prisma
University College London Hospitals, London	3T, Siemens, Prisma
Kings College, London	3T, Philips, Achieva
Barts Hospital, London	3T, Siemens, Prisma
Liverpool	3T, Siemens, Prisma

To model scanner vendor differences in multiorgan metrics we undertook paired scans in individuals across Siemens (Prisma) and Philips (Ingenio) scanner two weeks apart and considered the optimal approach for harmonization of metrics for individual organs (further details provided below).

MRI acquisition and analyses

Lung MRI

Typical Lung MRI parameters for Siemens Scan

1) Free breathing half-Fourier-acquisition single-shot turbo spin-echo (HASTE) MRI – [Axial; repetition time/echo time (TR/TE) = 750/49 ms; flip angle 120°, R=2; field of view = 380 × 320 mm^2, slice thickness/spacing 8/0mm, 35 slices, matrix = 256 × 123].
2) Inspiratory and expiratory radio-frequency–spoiled 3D gradient echo (GRE) sequence [coronal; TR/TE = 1.89/0.67 ms; flip angle 3°; field of view = 400 × 400 mm^2, slice thickness/spacing 3/1mm, 88 slices, voxel size 3.1 × 3.1× 3]. Images were repeated post-contrast injection.
3) Dynamic contrast enhanced gradient echo (GRE) sequence [coronal; TR/TE = 1.51/0.52 ms; R=2; flip angle 5; field of view = 400 × 400 mm^2, slice thickness/spacing 6/1mm, 40 slices/slab, voxel size 6.3x6.3x6.3].

Typical Lung MRI parameters for Philips Scan

1) Inspiratory and expiratory half-Fourier-acquisition single-shot turbo spin-echo (HASTE) MRI –[axial; TR/TE = 7135/45.5 ms, flip angle 90°, Sensitivity encoding (SENSE) = 2, field of view = 480 × 480mm^2, slice thickness/spacing = 10/0mm, 27-33 for full lung coverage, matrix =160 × 160, voxel size 1.88 × 1.88 × 10]
2) Inspiratory and expiratory radio-frequency–spoiled 3D gradient echo (GRE) sequence [coronal; TR/TE =1.90/0.60 ms, ; flip angle 3°; field of view = = 480 × 480mm^2, slice thickness/spacing = 5/2.5mm, 102-122 slices, matrix=160 × 160, voxel size =2 × 2 × 5]
3) Dynamic contrast enhanced gradient echo (GRE) sequence [coronal; TR/TE = 2.08/0.51ms, flip angle 15°, SENSE=2, field of view = 480 × 480mm^2, slice thickness/spacing = 10/0, 30 slices, 60 temporal acquisitions, temporal resolution = 1.10s, voxel size = 6 × 6 × 10mm].

Lung MRI Analysis

Axial thoracic HASTE images were qualitatively read by an expert radiologist (XC) and CMR reader (BR) and scored 0 or 1 based on presence of ≤5% parenchymal abnormalities or >5% parenchymal abnormality respectively. Extent of lung parenchymal abnornality was also evaluated as four categories - upto 25%, 26–50%, 51–75%, and 76–100%. Where there was a disagreement in extent of abnormalities, a third experienced radiologist (FG) served as an adjudicator. Semiquantitative analysis included analysis of HASTE signal density using images which were normalised to chest muscle signal. The images were segmented with visible vessels excluded from lung segmentations. Mean and coefficient of variance of normalised lung signal was calculated for each patient. Perfusion images are co-registered to reduce patient motion using advanced neuroimaging tools (ANTs).[5] Voxelwise maps of relative pulmonary blood volume (rPBV), mean transit time (MTT) and relative pulmonary blood flow (rPBF) were calculated for each patient.[6]A lung segmentation with major vessels excluded was applied to each map, and mean was calculated for each segmented parametric map.

Harmonisation of Lung MRI parameters

In order to allow the free breathing Seimens and expiratory Philips HASTE lung analysis to be combined, HASTE mean signal Z-score was calculated for each patient. The Z-score calculation used reference data from 9 healthy volunteers scanned on both Phillips and Seimens scanners during expiration and free breathing respectively (HASTE signal during the free breathing Seimens acquisition: 0.20±0.09; HASTE signal during the expiratory Phillips scans: 0.23±0.04).

Cardiac MRI

The cardiac MRI protocol included routine clinical and advanced parametric mapping sequences. The cardiac MRI sequences were as follows:

Typical cardiac scan parameters for 3T Siemens MRI

The cardiac MRI protocol included routine clinical and advanced parametric mapping sequences.

1) Cine steady-state free precession (SSFP) imaging [Three long-axis and short-axis stack; Typically TR/TE = 3.16/1.38ms; R = 3; flip angle = 65°; field of view = 360×270 mm, matrix 208×139, slice thickness/spacing 7/3mm].

2) Shortened Modified Look Locker T_1 mapping (ShMOLLI, Siemens prototype sequence WIP1048B) [Base, mid, apex short axis slices] was used for native and post-contract (15 minutes) acquisitions as described previously [7].

3) T_2 mapping (Siemens Myomaps product sequence) [Base, mid, apex short axis; bSSFP readout; TR/TE = 3/1.3ms, R = 2, flip angle = 20°, Matrix = 192×142, field of view = 360×270 mm, slice thickness = 8 mm].

4) Late gadolinium imaging was acquired using a T_1-weighted phase-sensitive inversion recovery sequence following a bolus injection of 0.15 mmol/kg of body weight of gadolinium-based contrast agent (Dotarem) and a 10 ml saline flush. Typical scan parameters: [TR/TE/TI = 3.1/1.22 ms/subject-specific ms; R = 2; flip angle = 55°; matrix = 144×256, field of view 380×285 mm, slice thickness = 8 mm, spacing = 2mm.]

Typical cardiac scan parameters for 3T Philips MRI

1) Cine SSFP imaging [Three long-axis and short-axis stack; Typically TR/TE = 3.6/1.82ms; R = 2; flip angle = 65°; field of view = 380×312 mm, matrix 224×184, slice thickness/spacing 7/4mm].

2) Modified Look Locker Inversion Recovery (MOLLI) T_1 mapping [Base, mid, apex short axis slices; TR/TE = 2.3/1.08ms, R = 2, flip angle = 20°, field of view = 300×300 mm, matrix = 256×256; slice thickness = 8 mm; 5-3s-3 inversion recovery sampling scheme].

3) T_2 Mapping (Black blood GraSE gradient and spin echo) [Base, mid, apex short axis slices; TR/TE = 1RR/10,20,30,40,50,60,70,80,90ms, R = 2, TSE factor 9, EPI factor 7, field of view 288×288 mm, matrix 288×288, slice thickness = 10mm].

4) Late gadolinium imaging was acquired using a T_1-weighted phase-sensitive inversion recovery sequence following a bolus injection of 0.15 mmol/kg of body weight of gadolinium-based contrast agent (Dotarem) and a 10 ml saline flush. Typical scan parameters: [TR/TE/TI = 6.11/2.99/subject-specific ms; R = 3; flip angle = 25°; matrix = 288×288, field of view 350×350 mm, slice thickness = 10 mm.]

5) T_1-weighted imaging: Base, mid and apical T_1 maps (MOLLI) were acquired 15 minutes after the administration of contrast agent.

Cardiac MRI image analysis

All quantitative MRI metrics were undertaken blinded by a single image analyst (EL) with over 5 years of experience in mapping image analysis and over 10 years in cardiac MRI data analysis. EL also underwent standardised training in image analysis as previously described. [8,9] Cardiac MRI analysis was undertaken using cvi42 software (Circle Cardiovascular Imaging Inc., Server Version 5.13.7 , Calgary, Canada). Volume and function: Left and right ventricular short axis epicardial and endocardial borders were manually contoured at end-diastole and end-systole (endocardial border only). Left and right ventricular end-diastolic (EDV) and end-systolic (ESV) volumes were used to calculate stroke volume (SV) as SV = EDV-ESV.[10] Ejection fraction (EF) was calculated as a ratio of SV and EDV (EF = SV/EDV). LV mass was calculated by subtracting the endocardial volume from the epicardial volume using the myocardial specific gravity as 1.05 g/cm^3.[11] Volumes and mass were indexed to body surface area. LV papillary muscles were included as part of LV end-diastolic and end-systolic volumes, and excluded from LV mass. Epicardial and endocardial contours were manually drawn on the base, mid and apical slices of the myocardium and average slice T_1 and global T_1 derived. Care was taken to avoid partial volume with the surrounding tissue, such as fat or blood pool. All T_1 and T_2 maps also underwent strict quality control. Manual epicardial and endocardial contours were drawn conservatively on base, mid and apical slices (acquired at identical slice positions to pre and post-contrast T_1 maps).

Average slice relaxation times were derived for each slice.[12]Extra-cellular volume (ECV) for the three short slices required additional contouring of post-contrast T_1 maps and contouring of regions of interest in the blood pool of pre and post-contrast T1 maps. ECV was calculated from pre- and post-contrast T_1 maps and haematocrit (Hct) using the formula: $ECV = (\Delta[1/T_1 myocardium]/\Delta[1/T_1 blood]) * [1-Hct])$.[13] Each person's average T_1 (pre- and post-contrast) and T_2 were assessed with a quality score based on the images quality: 0 – good quality; 1 – minor issues, <50% myocardial segments affected; 2 – major issues, >50% myocardial segments corrupted; 3 – not analysable or missing data. Scores 0-1 were considered reliable and adequate reportable myocardial coverage. Whereas estimates with the quality scores 2-3 were recommended for rejection from the final statistical analysis. Late gadolinium enhancement (LGE): Quantitative LGE analysis was performed in scans where LGE was present. Endocardial and epicardial borders were drawn on the phase sensitive inversion recovery (PSIR) images alone with a reference region of interest (ROI) with normal myocardial intensity as 'remote' region. Care was taken to exclude artefacts, blood pool, fat and pericardium. Using the 5SD approach, hyperenhanced pixels were quantified as a percentage of the myocardium and multiplied by the absolute left ventricular mass on cine images to determine LGE mass.

Blinded qualitative assessment of abnormal LGE images were undertaken by two experienced (Level 3 SCMR-accredited) cardiac MRI readers and cardiologists (CN, BR – more than 5 years of experience) and where there was a disagreement, a third experienced operator (VMF, 13 years of experience) served as the adjudicator. LGE was visually classified as follows: 1) no LGE, 2) possible/probable myocarditis pattern, 3) possible/probable myocardial infarction pattern, 4) LV/RV insertion point fibrosis, 5) mixed (infarction and myocarditis), 6) other (cardiomyopathy). All cases were also assessed for presence of significant pericardial effusion (>5mm).[14]

Harmonisation of T1 and T2 mapping

Analyses of mapping data from Siemens and Philips scanners from 9 subjects revealed, as expected, higher myocardial T1 and T2 values from Philips relative to Siemens. To address these differences, local normal ranges for T1- and T2-mapping were obtained directly from the sites according to SCMR guidelines [15], or ShMOLLI T1-mapping norms were used after the sequence conformance was validated using phantoms [16], as appropriate. The normal ranges were all expressed as normal mean ± SD for each site.[17] In line with prior observation [17], the observed variation in the reported normal SDs between sites precluded the meaningful application of z-scores; instead, the normalised nT1 and nT2 were reported, obtained by dividing the individual T1 and T2 measurements by the corresponding normal mean value for the appropriate site/scanner/sequence combination. There was no significant inter-scanner variability in ECV, and ECV thus was used directly, in line with the SCMR consensus.[13] Cardiac abnormalities were defined as an abnormality in indexed left ventricular and right ventricular volumes, function or pathological pattern late gadolinium enhancement or the combination of a T1 and T2 abnormality (i.e., nT1 or nT2 Z score > 2) involving a single short-axis slice.

Brain MRI

Typical Brain MRI parameters for Siemens 3T scanner

1) High-resolution T_1-weighted Magnetization Prepared RApid Gradient Echo (MPRAGE) – [$TR_{outer}/TR_{inner}/TE/TI$ = 2000/1.95/880 ms, Acceleration Factor=2, flip angle = 8°, Matrix = 256 × 256 × 208, voxel dimension = 1 mm isotropic].

2) Fluid Attenuated Inversion Recovery (T_2-FLAIR-SPACE) – [TR/TE/TI = 5000/386/1800 ms, Acceleration Factor=3, Matrix = 256 × 256 × 192, voxel dimension = 1 × 1 × 1.05 mm].

3) Diffusion MRI (dMRI) – [TR = 8489 ms, TE = 71 ms, flip angle = 90°, Matrix = 104 × 104 × 72, 6/8 Partial Fourier, voxel dimension = 2 mm isotropic, b=0, 1000 s/mm^2, 3 sequentially applied diffusion gradient directions (x,y,z), plus blip-reversed b=0]. With just 3 orthogonal diffusion directions (as commonly done in clinical practice), we can estimate mean diffusivity only. The diffusion sequence was kindly provided by the CMRR, University of Minnesota, United States of America.

4) Pseudo-continuous 2D Echo planar imaging (EPI) arterial spin labelling (ASL) perfusion (acquired when possible) TR_{max} = 4500 ms (shorter TR used for shorter PLDs), TE = 14 ms, Matrix = 64 × 64 × 24, 6/8 Partial Fourier, voxel dimension = 3.4 × 3.4 × 4.5 mm^3, pCASL label duration 1400 ms, Post Label Delays = [300ms, 600ms, 900ms, 1200ms, 1500ms, 1800ms] plus M0 image, static signal suppression.

5) Susceptibility-weighted MR Imaging (3D-swMRI) – [TR/TE1/TE2 = 27/9.4/20 ms, Matrix = 256 × 232 × 48, Acceleration Factor = 2, voxel dimension = 0.9 × 0.9 × 3 mm^3].

Typical Brain MRI parameters for Philips 3T scanner

1) High-resolution T_1-weighted Magnetization Prepared RApid Gradient Echo (MPRAGE) – [TR_{outer}/TR_{inner}/TE/TI = 2000/7/3.2/880 ms, Acceleration Factor=2, flip angle = 8°, Matrix = 256 × 256 × 208, voxel dimension = 1 mm isotropic].
2) Fluid Attenuated Inversion Recovery (T_2-Prepped-low-refocus-angle TSE FLAIR) – [TR/TE/TI = 5000/386/1800 ms, Acceleration Factor=3, Matrix = 256 × 256 × 192, voxel dimension = 1 × 1 × 1.05 mm].
3) Diffusion MRI (dMRI) – [TR = 8489 ms, TE = 73 ms, flip angle = 90°, Matrix = 112 × 110 × 72, 6/8 Partial Fourier, voxel dimension = 2 mm isotropic, b=0, 1000 s/mm^2, 3 sequentially applied diffusion gradient directions (x,y,z), plus blip-reversed b=0]. With just 3 orthogonal diffusion directions (as commonly done in clinical practice), we can estimate mean diffusivity only.
4) Pseudo-continuous 2D EPI ASL perfusion (acquired when possible) TR_{max} = 4600 ms (shorter TR used for shorter PLDs), TE = 14 ms, Matrix = 64 × 64 × 24, 6/8 Partial Fourier, voxel dimension = 3.4 × 3.4 × 4.9 mm^3, pCASL label duration 1400 ms, Post Label Delays = [300ms, 600ms, 900ms, 1200ms, 1500ms, 1800ms] plus M0 image, static signal suppression.
5) Susceptibility-weighted MR Imaging (3D-swMRI) – [TR/TE1/TE2 = 27/9.4/20 ms, Matrix = 256 × 232 × 48, Acceleration Factor = 2, voxel dimension = 0.9 × 0.9 × 3 mm^3].

Brain MRI analysis

Data were manually quality-control checked. Image processing was carried out using an adapted version of the processing pipeline that we created for UK Biobank brain imaging as previously described.[18-21]

This is largely based around tools from FSL (FMRIB Software Library)[22] and FreeSurfer[23]. Data were corrected for gradient and EPI distortions, aligned to each other using linear alignment[23], and then aligned into standard template space (MNI152) using nonlinear alignment applied to the T1.[24,25] T_1 images were further segme[21]nted probabilistically into different tissue types[26] and also into different subcortical structures.[27] T2-FLAIR images were also segmented, to identify while matter hyperintensities (WMH).[28] pWMH (periventricular WMH) and dWMH (deep WMH) volumes were extracted as subsets of WMH, using the criterion of being less than (or more than, respectively) 10 mm distant from the lateral ventricles.[29,30] swMRI data was processed in 3 distinct ways: 1) The two echoes were combined to provide a quantitative mapping of T_2*. 2) The phase and magnitude data were processed to provide maps highlighting features such as microbleeds. Qualitative assessment of brain MRI findings were also undertaken by an experienced neuroradiologist.

Harmonisation

After review of brain MRI quantitative data from 9 subjects scanned across Philips and Siemens, no discernable systematic bias was evident across volumetric measures. Given the limited sample size of cross-scanner subject data from the harmonization experiment, scanner hardware type and scanner site were used as covariates in the assessment of group differences in volumetric measures.

Liver MRI

Liver imaging used the LiverMultiScan acquisition protocol (Perspectum, Oxford, UK), which involves up to 4 axial breath-held acquisitions that separately are sensitive to the fat content (proton density fat fraction [PDFF]), to T2* (which can yield liver iron content) and to MOLLI-T1 (providing a measurement of tissue water).

Typical Liver MRI parameters for 3T Siemens scanner

1) LiverMultiScan MOLLI: Axial; TR/TE = 365 ms/1.05 ms, flip angle 35°, FOV = 440 × 330 mm^2; slice thickness/spacing = 8 mm/7 mm, matrix = 192 × 144, voxel size = 2.3 × 2.3 × 8 mm^3, initial TI = 100 ms, TI increment = 80 ms, iPat 2 [ECG-gated shMOLLI sampling pattern and conditional processing].[7]
2) LiverMultiScan IDEAL: Axial; TR/TE/Echo spacing = 15 ms/1.1 ms/1.1 ms, flip angle = 3°, FOV = 440 x 398.7 mm^2, slice thickness/spacing = 10/5 mm, matrix = 128 × 116.
3) LiverMultiScan T2star Dixon: Axial; TR/TE/echo spacing = 500 ms/1.23 ms/1.23 ms, flip angle = 20°, FOV = 400 x 325 mm^2, slice thickness = 6 mm, matrix = 128 × 104, iPat 2, cardiac gated.
4) LiverMultiScan MOST: Axial; TR/TE/Echo spacing = 11 ms/1.31-1.23ms, flip angle = 9°, FOV = 440 x 357mm^2, slice thickness = 3 mm, matrix = 128 × 104, iPat 2.

Typical Liver MRI parameters for 3T Philips scanner

1) LMS MOLLI: Axial; TR/TE = 2.37 ms/1.05 ms, flip angle = 35°, FOV= 440 × 440 mm^2, slice thickness/spacing = 8 mm/7 mm, matrix = 192 × 192, SENSE 2.

2) LMS IDEAL: Axial; TR/TE/echo spacing = 15 ms/1.1 ms, flip angle = 5°, FOV= 440 × 440 mm^2, slice thickness/spacing = 10 mm/15 mm, matrix = 128 × 129.
3) LMS T2Star DIXON: Axial; TR/TE = ~20.24 ms/4.74 ms, flip angle = 20°, FOV = 400 × 400 mm^2, slice thickness = 6 mm, matrix = 128 × 128, SENSE 2.
4) LMS MOST: Axial; TR/TE = ~11.27 ms/8.49 ms, flip angle = 11°, FOV= 440 × 440 mm^2, slice thickness = 3 mm, matrix = 140 × 142, SENSE 2.

Liver MRI Analysis

Anonymized liver MRI data were processed and analyzed centrally by expertly trained image analysts who were blinded to the clinical data, using LiverMultiScan software (Perspectum, Oxford, UK). The analysis yielded global metrics in each liver of percentage fat (PDFF; proton density fat fraction), T2* (related to liver iron concentration), and cT1 (cT1 is a measurement of T1 that has been corrected for the confounding effects of iron and standardised across scanner manufacturers and field strengths; it is elevated in the presence of liver fibrosis and inflammation and predicts liver outcomes).[31,32] The T2* (milliseconds) and the PDFF values (%) were calculated from the average of all pixels within the liver region defined by manually-assisted automatic segmentation and artefact detection, as described previously.[33] For T2* segmentation was from repeated acquisitions of thin slice GRE data enabling higher signal-to-noise. The cT1 values (milliseconds) were calculated from the median of all pixels within the liver region defined by manually-assisted automatic segmentation and artefact detection. Liver iron concentration (LIC), expressed in mg Fe/mg dry weight tissue, provided the iron concentration of hepatic tissues and was derived from T2* by linear regression from 1/T2*.[34]

Harmonisations of measurements

For cT1, the confounding effect of iron on T1 mapping is corrected by a compensatory algorithm, based on the application of a multi-compartment model to simulate tissue and water environments in the liver during changes in iron content and in extracellular fluid (a proxy for fibrosis)[35], standardised to a 3T field strength.
The repeatability, reproducibility, and intra- and inter-operator reliability of cT1, T2*, and PDFF measurements across scanner field strength, manufacturer, and model have been systematically tested in both human participants and phantoms.[36] These studies have shown excellent repeatability and reproducibility for derived metrics, enabling utility in multi-site trials for liver disease[37,38] and large-scale population screening.[39]

Kidney MRI

Typical Kidney MRI parameters for 3T Siemens scanner

1) 2D T2-weighted HASTE sequence optimized to achieve the maximum contrast between the kidneys and surrounding tissue TE = 61 ms, TR = 438ms, GRAPPA factor = 3, refocus angle 120°, bandwidth 781 Hz, FOV = 384 × 384 mm2, voxel size = 1.5 × 1.5 × 5 mm3 with approximately 26 coronal slices, enough to image the entire kidney in a single 15-s breath-hold.
2) Kidney MOLLI T_1 map with a 5-3s-3 sampling scheme[40] [oblique coronal; TR/TE = 2.7/1.15 ms; R = 4; flip angle = 35°; field of view 320 × 320 mm2, matrix = 194 × 192, slice thickness 5.5 mm, simulated heart rate (HR) = 1s, TI_1 = 100 ms, TI_2 = 180 ms] was used to assess inflammation and fibrosis.
3) Kidney multi echo gradient echo[41] [oblique coronal; TR/TE = 81 ms/9.84 ms with 4.92 ms echo spacing and 12 echoes; GRAPPA factor = 3, flip angle = 25°; field of view 288□288mm^2, matrix = 192 × 192 but resampled to 288, slice thickness 5.0 mm] was used to assess renal oxygenation via R2*. Both magnitude and phase data were saved.

Typical Kidney MRI parameters for 3T Philips scanner

1) 2D T2-weighted HASTE sequence optimized to achieve the maximum contrast between the kidneys and surrounding tissue TE = 60 ms, TR = 928 ms, CS SENSE factor = 4 or SENSE = 2.5, refocus angle 120°, bandwidth, 792 Hz, field of view = 350 × 350 mm2, voxel size = 1.5 × 1.5 × 5 mm3, 17 coronal slices to image the entire kidney in a single 16-s (CS-SENSE) or 18.8-s (SENSE) breath-hold.
2) Kidney MOLLI T_1 map with a 5-3s-3 sampling scheme[40] [oblique coronal; TR/TE = 2.5/1.2 ms; R = 2; flip angle = 35°; field of view 320 × 320 mm2, matrix = 288 × 288, slice thickness 5.5 mm, simulated heart rate (HR) = 1s, TI1 = 102 ms, TI2 = 180 ms] was used to assess inflammation and fibrosis.
3) Kidney multi echo gradient echo (ME-GE)[41] [oblique coronal; TR/TE = 81 ms/4.6 ms with echo spacing of 4.6 ms and 12 echoes; SENSE factor =3; flip angle = 25°; field of view 288 × 288mm2, matrix = 288 × 288, slice thickness 5.0 mm] was used to assess renal oxygenation via R_2*. Both magnitude and phase data were saved, with the phase difference between the first two echoes used to generate a B_0 map.

Kidney MRI Analysis

A 2D convolutional neural network (CNN) was used to segment the left and right kidneys from the T_2-weighted MRI data[42] and compute total kidney volume (TKV). The CNN was retrained on both Philips and Siemens data for improved segmentation, and to account for an error in acquisition on data collected resulting from wrapping artefacts in selected cases where phase encoding direction was set to foot to head rather than right to left.

T_1 maps were computed from the MOLLI data. T_1 mapping was based on the three-parameter curve fitting of the data to A – B exp(–x/T 1) using the Levenberg–Marquardt algorithm, and R squared (R^2) and residual maps for quality control. The kidney was segmented by the T_2-weighted scans, and then regions of interest (ROI) of the renal cortex and medulla formed based on histogram analysis of the T_1 maps[43] with the upper and lower semi-quartiles removed to ensure a robust medulla and cortex mask. This ensured that the outer border between renal parenchyma and perirenal fat or renal sinus fat which are prone to partial volume averaging artifacts and possible residual registration error were not included, and banding artifacts in the kidney due to off-resonance were avoided, since these artifacts can cause significant error at relatively small off-resonance frequencies. The mean T_1 in left and right cortex and medulla were computed.

Harmonisations of mapping

Data collected 9 healthy volunteers scanned across Siemens (Prisma) and Philips (Achieva) were used to compare measures of TKV and T_1 measures in the cortex and medulla. A significant difference between the manual TKV and automatic TKV was observed when using the pretrained CNN (p=0.00007), whereas this became insignificant when using the retrained CNN (p=0.21). No significant difference in measured TKV was reported between vendors using the retrained CNN (p=0.95) nor was any significant difference in segmentation accuracy across vendors observed.

Definition of multiorgan abnormalities

Multiorgan abnormalities are defined by the presence of MRI abnormalities involving two or more organs. On MRI, cardiac abnormalities are defined by the presence of abnormal slice-level T1 and T2 values (abnormal refers to values that are more than two standard deviations from age and sex-matched non-COVID-19 control range), or the presence of pathological LGE, or abnormal ECV, or abnormal LV or RV ejection fraction. Lung abnormalities are defined by the presence of qualitative abnormalities involving the lung parenchyma T2 HASTE. Renal abnormalities are defined by abnormal cortical or medullary T1, abnormal corticomedullary differentiation, or abnormal renal volumes. Liver abnormalities are defined by the presence of abnormal liver proton density fat fraction, cT1, or iron content. Brain abnormalities are defined by abnormal brain volumes or qualitative abnormalities as assessed on clinical readouts.

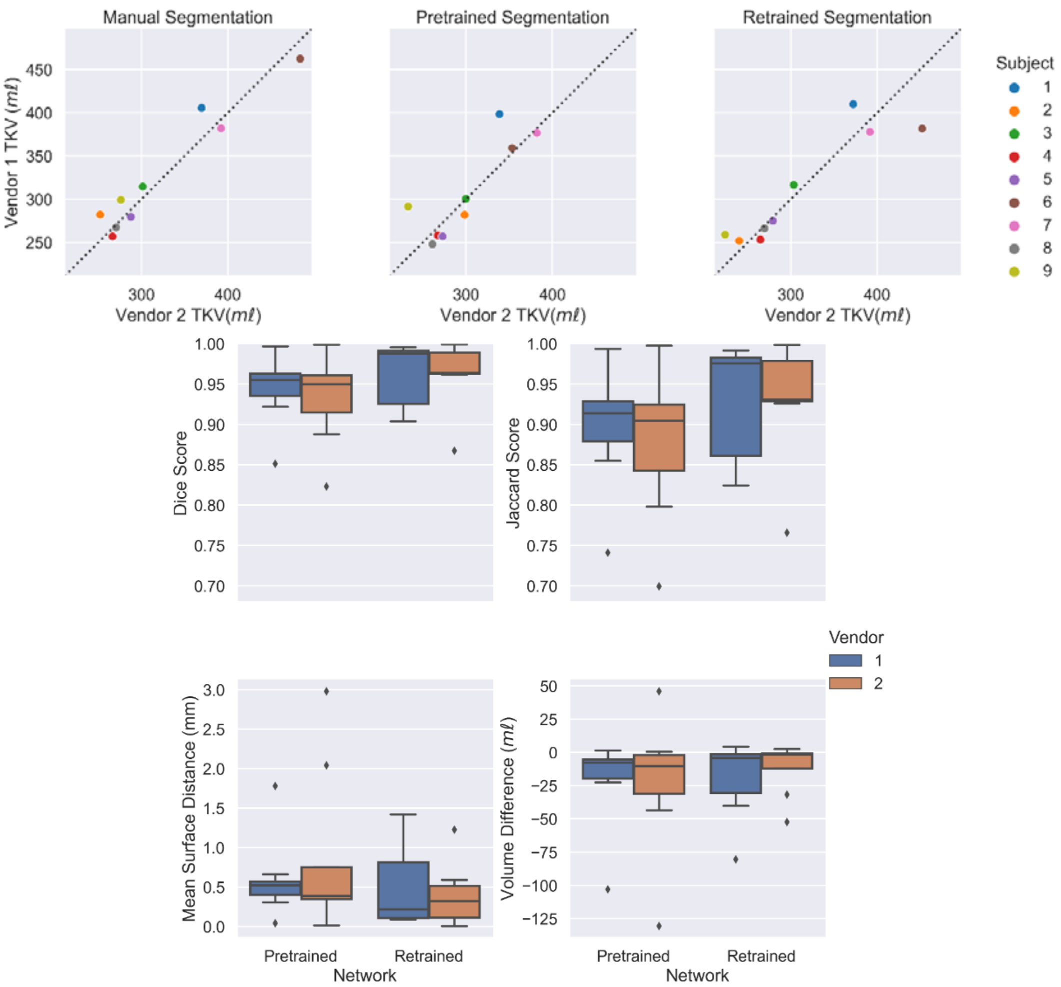

Figure 1. Segmentation of total kidney volume from manual approach and retained CNN for Siemens and Philips.

A significant difference in Dice, Jaccard and volume difference was observed between vendors when the manual masks were compared to those generated using the pretrained CNN (p=0.002, 0.001, 0.02 respectively), these became insignificant when segmentations were generated using the retrained CNN (p=0.46, 0.40, 0.88 respectively). These metrics are summarised in **Figure 2**.

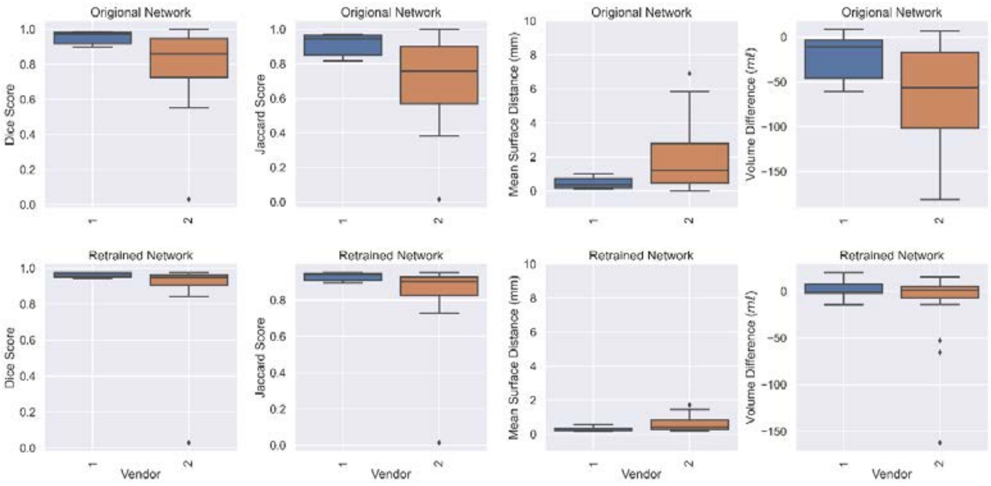

Figure 2: Intervendor differences following manual and automated segmentation across scanners.

There was no significant bias in T_1 measures between vendors for left and right kidneys.

	Cortex		Medulla	
	Paired test P value	Bland-Altman bias ± SD (ms)	Paired test P value	Bland-Altman bias ± SD (ms)
Right kidney	0.5	-7 ± 30	0.07	-23 ± 33
Left kidney	0.9	2 ± 55	0.2	28 ± 59

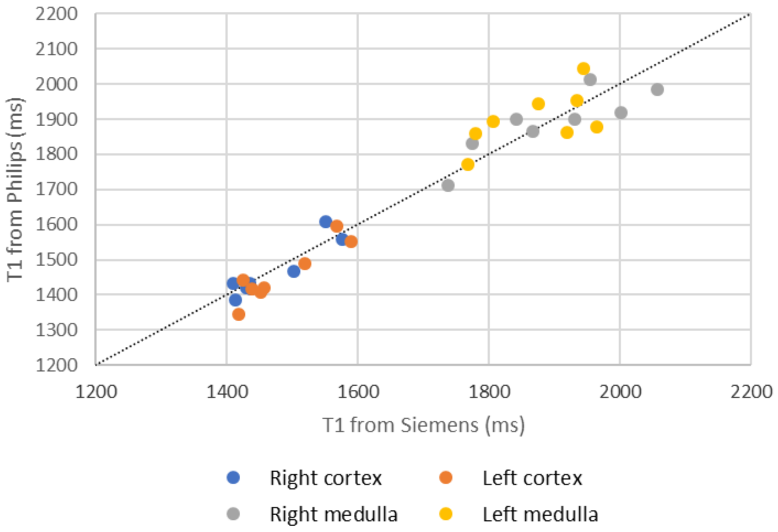

Figure 3: Level of agreement in cortical and medullary T1 across Siemens and Philiips scanners.

Spirometry

Spirometry measurements were undertaken in triplicate using a spirometer, according to the criteria established by the American Thoracic Society and European Respiratory Society.[44] Spirometry measures were not repeated after administration of bronchodilators. The largest FEV_1 and FVC of the triplicate readings and respective FEV_1/FVC ratio were reported.

Patient related outcomes Measures

Patient health questionnaire (PHQ-9)

The Patient Health Questionnaire depression module (PHQ-9),[45] a multipurpose instrument for screening, diagnosing and monitoring depression, was administered to assess for evidence of depression.[46] Scores of 5, 10, and 15 were used as cut-offs mild, moderate, and severe depression, respectively and PHQ9≥10 was considered significant.

General Anxiety Disorder Questionnaire (GAD-7)

The Generalised Anxiety Disorder Assessment (GAD-7),[46] a seven-item instrument, was used to screen for and measure the severity of generalised anxiety disorder among participants. GAD-7≥ 8 represented moderate and severe anxiety.

Montreal Cognitive Assessment (MOCA) Tool

Cognitive function was assessed by using the MOCA,[47] with scores ranging from 0 to 30 points. A score of, <23 was considered significant cognitive impairment.

Dyspnoea-12 questionnaire

The Dyspnoea-12 questionnaire is a patient reported outcome measure that consists of 12 questions and assesses breathlessness severity incorporating both “physical” and “affective” aspects. The scores range from 0 to 36 with higher scores correspond to greater severity of breathlessness.[48]

FACIT-F

The Functional Assessment of Chronic Illness Therapy – Fatigue (FACIT-Fatigue) scale was used. This patient reported outcome measure consists of 13 questions to assess self-reported fatigue and its impact on daily activities and function. Total scores range from 0-52, with lower scores corresponding to an increased burden of fatigue.[49]

PCL-5

The Post-Traumatic Stress Disorder Checklist for DSM V (PCL-5) questionnaire is a patient reported outcome measure consisting of 20 questions assessing evidence of post-traumatic stress disorder according to the DSM V criteria. Total scores range from 0-80. A PCL-5 threshold score of ≥38 was regarded as abnormal and suggestive of a provisional diagnosis of post-traumatic stress disorder.[50]

Symptom and recovery questionnaire

A bespoke study-specific symptom questionnaire[1] was also administered to cover multi-organ manifestations including chest pain, shortness of breath, abdominal pain, nausea, vomiting, headache, brain fog.

Patient perceived-recovery was also assessed using a study specific recovery question “Do you feel fully recovered?”; participants could answer “yes”, “no”, or “not sure”.

PHOSP-COVID Symptom clusters

Patients were categorized into clusters derived from unsupervised cluster analysis[1] of patients recovery measures collected as part of the PHOSP-COVID study. These included symptom questionnaires (patient-reported outcome measures) and physical performance and cognitive assessment data (Dyspnoea-12, FACIT-F Fatigue questionnaire, Post-traumatic stress disorder questionnaire (PCL-5), GAD-7, PHQ-9, short physical performance battery, and MOCA as continuous variables) from the 5-month visit (discharge dates March 7, 2020, to April 18, 2021) using the clustering large applications k-medoids approach. Cluster membership was determined for each individuals.

Cluster 1 or mild cluster referred to individuals with mild/no impairment in physical, mental or cognitive performance

Cluster 2 or moderate cluster referred to individuals with more significant impairment in cognition, but either mild of no impairment of physical and mental health domains.

Cluster 3 or severe cluster referred to individuals with moderate physical and mental health impairment, but preserved cognition

Cluster 4 or very severe cluster referred to individuals with severe physical and mental health impairment, but preserved cognition (**Figure 4**).

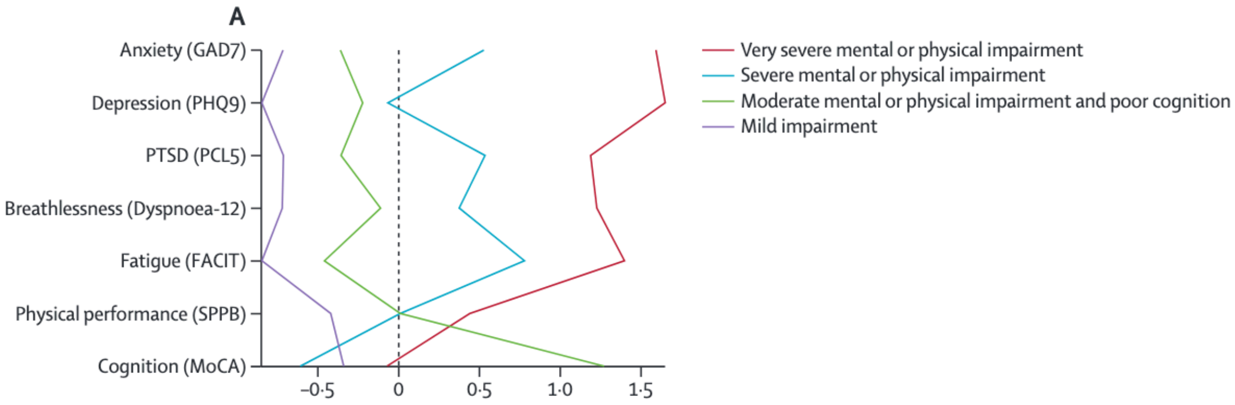

Figure 4. Clusters of mental, physical and cognitive standardized scores of patient reported outcome measures.[1] (Image adapted from Evans et al. Lancet Respir Med 2021; 9(11): 1275-87 with permission)

RESULTS

Comparison between C-MORE versus non-C-MORE Tier 2 PHOSP-COVID cohort

Compared to non-C-MORE Tier 2 PHOSP-COVID cohort, the C-MORE population were matched for age, sex, comorbidity count and smoking. However, C-MORE had significantly lower proportion of individuals admitted to ICU for invasive mechanical ventilation (7.0% vs 18.9%, p<0.0001) and thus short admission duration (median (IQR) 6 (3-10) days vs 8(4-17) days (p<0.0001)). BMI was also lower among C-MORE participants with mean(SD) BMI 30.6 (5.72) kg/m^2 vs 32.4 (7.04) kg/m^2 (P=0.0002).

Vaccination status

A similar proportion of controls and patients received at least one vaccination (41% (20/49) versus 112/257 (43·6%)) by the time of the MRI scan.

Burden of multiorgan injury (following adjustment for Charlson comorbidity index)

Multiorgan injury was nearly three times (OR 3.01. 95% more common in patients post-hospitalisation for COVID-19 when compared to controls after adjusting for relevant confounders including charlson comorbidity index.

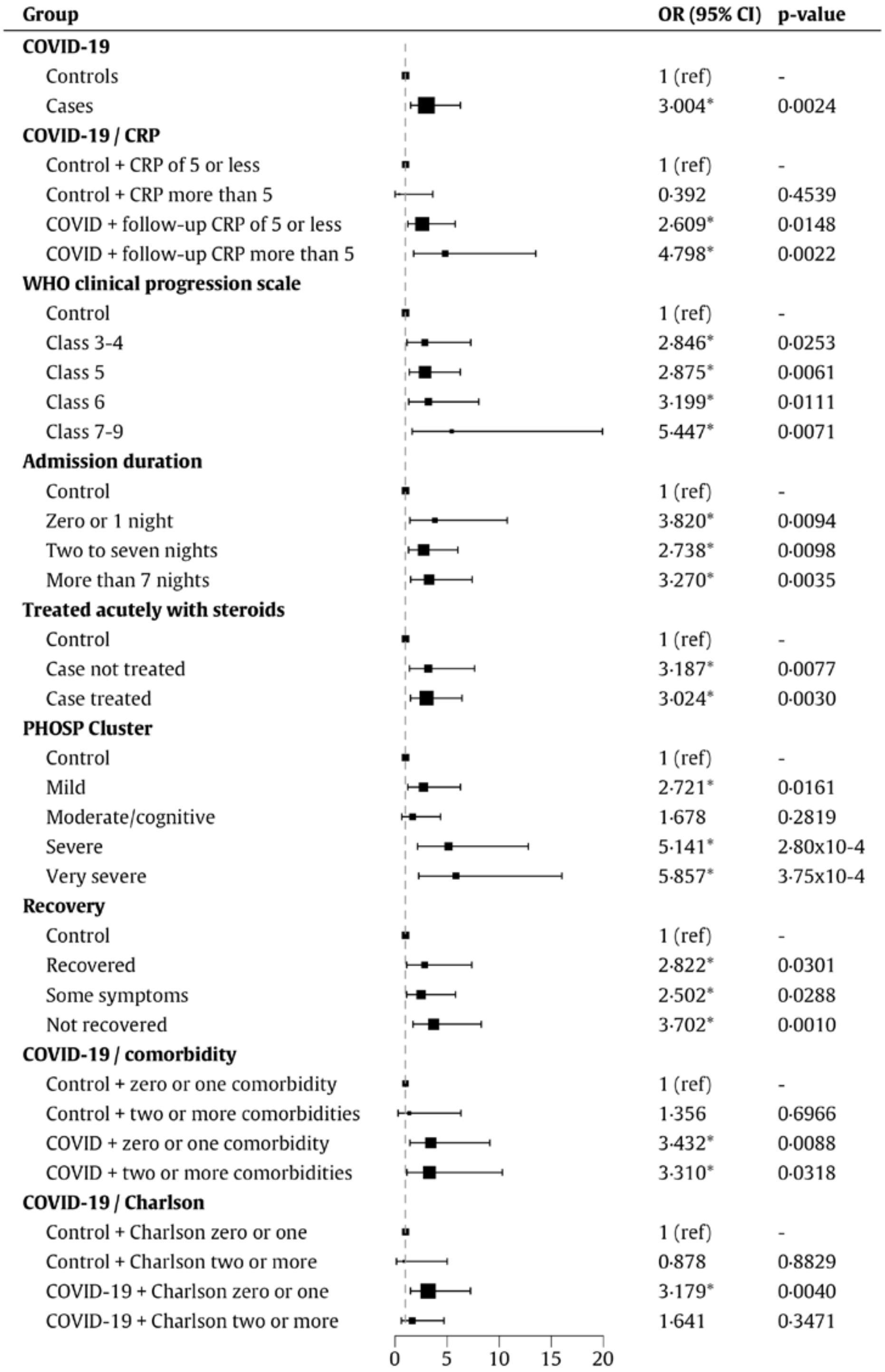

Figure 4. Determinants of multiorgan injury on MRI among post-hospitalised patients recovering from COVID-19. Forest plot depicts the effect of hospitalisation for COVID-19 on medium-term multiorgan health stratified by WHO severity, Charlson comorbidity index, severity of acute infection, inflammatory burden and recovery status relative to controls

Tables Legend

Supplementary Table 1. A) Comparison of lung MRI in patients vs controls; B) Clinical characteristics of patients with and without lung MRI abnormalities; C) Clinical characteristics of patients with and without lung MRI abnormalities – excluding pre-existing respiratory conditions.

Part A: Comparison of lung health between patients versus controls

					Multivariate analysis - adjusted inverse probability weighting (IPW)		Multivariate analysis - adjusted with IPW and excluding patients with WHO >= 7		
**Variable**	**Controls (n= 52)**	**Patients (n= 259)**	**P-value**	**Univariate test**	**Odds Ratio or Beta coefficient**	**p-value**	**Odds Ratio or Beta coefficient**	**p-value**	**Test**
Respiratory comorbidity	8 (15.4%)	81 (31.5%)	**0.030**	X²	1.61 (0.79, 3.50)	0.205	1.71 (0.84, 3.74)	0.155	Logistic
WHO clinical progression scale									
WHO – class 3-4		45 (17.6%)							
WHO – class 5		141 (55.3%)							
WHO – class 6		51 (20.0%)							
WHO – class 7-9		18 (7.1%)							
Follow-up Lung Function									
FVC (L)	5.00 (±1.17)	3.61 (±0.97)	**<0.001**	T/Welch	-1.22 (-1.62, -0.82)	**<0.001**	-1.18 (-1.58, -0.79)	**<0.001**	Linear
FEV1 (L)	3.71 (±0.98)	2.82 (±0.82)	**<0.001**	T/Welch	-0.98 (-1.39, -0.57)	**<0.001**	-0.96 (-1.37, -0.55)	**<0.001**	Linear
Ratio of FEV1 and FVC	0.74 (±0.06)	0.78 (±0.09)	**0.004**	T/Welch	0.61 (0.19, 1.03)	**0.005**	0.57 (0.14, 1.00)	**0.010**	Linear
FEV1 < 80% of predicted n (%)	1 (3.6%)	30 (22.9%)	**0.038**	X²	13.9 (1.9, 1178.9)	0.056	13.2 (1.8, 1126.4)	0.061	Logistic
FVC < 80% of predicted n (%)	0 (0.0%)	29 (22.1%)	**0.013**	X²		0.989		0.989	Logistic
Ratio FEV1:FVC less than 0.7	4 (14.3%)	24 (13.8%)	1.000	Fisher	1.23 (0.38, 5.27)	0.751	1.16 (0.35, 5.02)	0.822	Logistic
TLCO mmol/min/kPa (SI)		7.79 (±2.11)							
KCO mmol/min/kPa (SI)		1.45 [1.29, 1.60]							
**Lung MRI metrics**									
**Any lung abnormality (>5%)**	3 (6.1%)	90 (34.7%)	**<0.001**	X²	14.7 (4.0, 114.6)	**<0.001**	13.8 (3.8, 108.2)	**0.001**	Logistic
Lung parenchymal abnormality									
Less than 25%	49 (100.0%)	215 (83.0%)	**0.005**	Fisher					
26% to 50%		34 (13.1%)							
51% to 75%		9 (3.5%)							
Greater than 75%		1 (0.4%)							
Lung MRI									
Lung parenchymal average T2-HASTE signal intensity Z score	0.35 [-0.20, 1.02]	1.10 [0.33, 2.17]	**<0.001**	Mann-U	0.41 (0.10, 0.71)	**0.009**	0.37 (0.06, 0.68)	**0.018**	Linear
Lung T2-HASTE mean signal	0.23 (±0.08)	0.29 (±0.09)	**<0.001**	Mann-U	0.54 (0.23, 0.86)	**<0.001**	0.51 (0.19, 0.83)	**0.002**	Linear
Lung T2-HASTE signal standard deviation	0.15 [0.12, 0.17]	0.18 [0.15, 0.22]	**<0.001**	Mann-U	0.57 (0.26, 0.89)	**<0.001**	0.57 (0.25, 0.88)	**<0.001**	Linear
Pulmonary blood volume (area under the curve mean)	18.5 (±6.4)	17.1 (±6.8)	0.364	Mann-U	-0.25 (-0.85, 0.35)	0.413	-0.24 (-0.85, 0.36)	0.424	Linear
Pulmonary blood flow (area under the curve mean)	85.6 [61.2, 108.0]	72.1 [51.3, 100.1]	0.191	Mann-U	-0.30 (-0.89, 0.29)	0.324	-0.28 (-0.87, 0.31)	0.352	Linear
Pulmonary blood mean transit time (area under the curve mean)	12.9 [10.7, 14.5]	13.2 [11.5, 15.3]	0.228	Mann-U	0.12 (-0.47, 0.72)	0.682	0.09 (-0.50, 0.68)	0.773	Linear
Pulmonary blood volume median	16.1 (±5.6)	14.8 (±5.8)	0.323	Mann-U	-0.27 (-0.87, 0.34)	0.383	-0.26 (-0.87, 0.35)	0.395	Linear
Pulmonary blood flow median	80.4 (±32.6)	69.1 (±31.9)	0.180	Mann-U	-0.28 (-0.88, 0.31)	0.351	-0.26 (-0.85, 0.33)	0.390	Linear
Mean transit time median	12.4 (±2.1)	13.3 (±3.8)	0.347	Mann-U	0.01 (-0.58, 0.60)	0.982	-0.01 (-0.59, 0.58)	0.978	Linear

Part B: Clinical characteristics of patients with and without lung MRI abnormalities

Variable	No lung abnormalities (n= 169)	Lung abnormalities (n= 90)	Un-dadjusted p-value	Un-adjusted test	Odds Ratio or Beta coefficient	p-value	Test	Total sample size	No-abn N	Abn N
Age (years)	56·4 (±12·7)	58·1 (±11·4)	0.265	T/Welch	1.73 yrs [-1.42, 4.87]*			259	169	90
Female n (%)	63 (37·3%)	38 (42·2%)	0.520	X^2				259	169	90
Non-white ethnicity n (%)	45 (26·6%)	27 (30·0%)	0.666	X^2	1·06 (0·56, 1·99)	0.846	Logistic	259	169	90
Obesity n (%)	91 (53·8%)	38 (42·2%)	0.099	X^2	0·64 (0·38, 1·09)	0.101	Logistic	259	169	90
Smoking: Never smoker n (%)	101 (59·8%)	54 (60·0%)	0.201	Fisher				259	169	90
Smoking: Ex-smoker n (%)	63 (37·3%)	29 (32·2%)								
Smoking: Current smoker n (%)	5 (3·0%)	7 (7·8%)								
Respiratory comorbidity	56 (33·5%)	25 (27·8%)	0.420	X^2	0·74 (0·40, 1·32)	0.309	Logistic	257	167	90
Charlson index zero or one n (%)	142 (84·0%)	73 (81·1%)	0.603	Fisher				259	169	90
Charlson index of two or more n (%)	27 (16·0%)	17 (18·9%)								
WHO clinical progression scale										
WHO – class 3-4	30 (18·2%)	15 (16·7%)	0.167	Fisher				255	165	90
WHO – class 5	97 (58·8%)	44 (48·9%)								
WHO – class 6	30 (18·2%)	21 (23·3%)								
WHO – class 7-9	8 (4·8%)	10 (11·1%)								
Acute pulmonary injury n (%)	6 (3·6%)	7 (7·8%)	0.231	Fisher	2·86 (0·87, 9·87)	0.084	Logistic	255	165	90
Acute cardiac injury n (%)	16 (9·7%)	22 (24·4%)	**0.003**	X^2	3·04 (1·48, 6·37)	**0.003**	Logistic	255	165	90
Acute liver injury n (%)	92 (58·6%)	49 (56·3%)	0.834	X^2	0·98 (0·57, 1·71)	0.955	Logistic	244	157	87
Acute kidney injury n (%)	27 (16·4%)	15 (16·7%)	1.000	X^2	0·96 (0·44, 2·06)	0.924	Logistic	255	165	90
Admission duration (days)	5·00 [3·00, 9·00]	8·0 [3·2, 12·8]	**0.018**	Mann-U	0·32 (0·06, 0·58)	**0.015**	Linear	255	165	90
Abnormal chest x-ray n (%)	18 (21·7%)	19 (37·3%)	0.079	X^2	2·11 (0·91, 4·98)	0.082	Logistic	134	83	51
Treatments										
Proning n (%)	14 (10·2%)	16 (20·8%)	0.054	X^2	2·48 (1·09, 5·76)	**0.031**	Logistic	214	137	77
Remdesivir n (%)	21 (26·2%)	8 (22·9%)	0.879	X^2	0·75 (0·27, 1·91)	0.553	Logistic	115	80	35
Systemic (oral or IV) steroids n (%)	128 (77·6%)	63 (70·8%)	0.297	X^2	0·68 (0·37, 1·25)	0.208	Logistic	254	165	89
Therapeutic dose anti-coagulation n (%)	83 (50·3%)	49 (54·4%)	0.616	X^2	1·47 (0·85, 2·58)	0.170	Logistic	255	165	90
Non-steroidal anti-inflammatory (NSAID) n (%)	19 (11·5%)	9 (10·0%)	0.873	X^2	0·75 (0·30, 1·75)	0.521	Logistic	255	165	90
Follow-up Lung function										
FEV1 (L)	2·88 (±0·86)	2·71 (±0·72)	0.156	T/Welch	-0·06 (-0·32, 0·20)	0.665	Norm linear	174	111	63
FEV1 < 80% of predicted n (%)	19 (22·6%)	11 (23·4%)	1.000	X^2	1·09 (0·44, 2·63)	0.847	Logistic	131	84	47
FVC (L)	3·73 (±0·98)	3·40 (±0·94)	**0.029**	T/Welch	-0·21 (-0·47, 0·05)	0.117	Norm linear	174	111	63
FVC < 80% of predicted n (%)	16 (19·0%)	13 (27·7%)	0.358	X^2	1·63 (0·67, 3·89)	0.275	Logistic	131	84	47
FEV1:FVC	0·77 (±0·10)	0·80 (±0·08)	**0.018**	T/Welch	0·39 (0·10, 0·67)	**0.008**	Norm linear	174	111	63
Ratio FEV1:FVC less than 0.7 n (%)	18 (16·2%)	6 (9·5%)	0.317	X^2	0·55 (0·18, 1·48)	0.256	Logistic	174	111	63
TLCO mmol/min/kPa (SI)	8·14 (±2·07)	7·15 (±2·06)	**0.043**	T/Welch	-0·39 (-0·81, 0·03)	0.069	Linear	82	53	29
KCO mmol/min/kPa (SI)	1·43 [1·31, 1·59]	1·45 [1·20, 1·60]	0.484	Mann-U	-0·11 (-0·46, 0·24)	0.531	Linear	81	52	29
Do you feel recovered? n (%)										
No	67 (44·7%)	54 (64·3%)	**<0.001**	Fisher				234	150	84
Not sure	42 (28·0%)	7 (8·3%)								
Yes	41 (27·3%)	23 (27·4%)								
Reported outcomes										
Cough n (%)										
Breathlessness n (%)	73 (49·0%)	49 (59·0%)	0.183	X^2	1·64 (0·94, 2·91)	0.086	Logistic	232	149	83
Chest pain n (%)	32 (21·1%)	18 (21·4%)	1.000	X^2	1·13 (0·55, 2·25)	0.740	Logistic	236	152	84
Chest tightness n (%)	43 (28·3%)	36 (43·4%)	**0.028**	X^2	2·61 (1·40, 4·97)	**0.003**	Logistic	235	152	83
Joint pain n (%)	60 (39·5%)	46 (55·4%)	**0.027**	X^2	2·13 (1·21, 3·81)	**0.009**	Logistic	235	152	83
Dyspnoea-12 score	3·00 [0·00, 9·00]	3·00 [0·00, 10·75]	0.233	Mann-U	0·23 (-0·04, 0·50)	0.092	Norm linear	224	138	86

FACIT V4 Score	35·8 (±12·1)	35·4 (±11·5)	0.845	T/Welch	-0·13 (-0·38, 0·13)	0.341	Norm linear	224	138	86
Anxiety (GAD-7 score)	3·50 [0·00, 8·00]	3·00 [1·00, 7·75]	0.938	Mann-U	0·10 (-0·16, 0·36)	0.441	Norm linear	226	140	86
Anxiety (GAD-7 >8) n (%)	29 (20·7%)	18 (20·9%)	1.000	X²	1·28 (0·61, 2·63)	0.510	Logistic	226	140	86
Depression (PHQ-9) score	6·59 (±6·35)	7·32 (±6·47)	0.412	T/Welch	0·22 (-0·04, 0·48)	0.102	Norm linear	224	139	85
Depression (PHQ-9 >= 10) n (%)	45 (32·4%)	23 (27·1%)	0.490	X²	0·97 (0·51, 1·82)	0.916	Logistic	224	139	85
EQ5D-5L utility score	77·8 [66·9, 95·3]	75·0 [63·4, 87·8]	0.117	Mann-U	-0·28 (-0·54, -0·01)	**0.041**	Linear	226	143	83
EQ5D-5L less than 60%, n (%)	21 (14·7%)	18 (21·7%)	0.246	X²	2·18 (1·02, 4·69)	**0.044**	Logistic	226	143	83

Part C: Clinical characteristics of patients with and without lung MRI abnormalities after excluding those with pre-existing respiratory conditions

Variable	No lung abnormalities (n= 113)	Lung abnormalities (n= 90)	P-value	Univariate test	Odds Ratio or Beta coefficient	p-value	Test	Total N	No-abn N	Abn N
Age (years)	55.7 (±12.8)	57.2 (±12.0)	0.418	T/Welch	1.71 yrs [-2.15, 5.58]*			178	113	65
Female n (%)	40 (35.4%)	25 (38.5%)						178	113	65
Non-white ethnicity n (%)	37 (32.7%)	20 (30.8%)	0.916	X²	0.82 (0.39, 1.66)	0.579	Logistic	178	113	65
Obesity n (%)	58 (51.3%)	28 (43.1%)	0.366	X²	0.74 (0.39, 1.38)	0.347	Logistic	178	113	65
Smoking: Never smoker n (%)	74 (65.5%)	39 (60.0%)	0.295	Fisher				178	113	65
Smoking: Ex-smoker n (%)	36 (31.9%)	21 (32.3%)								
Smoking: Current smoker n (%)	3 (2.7%)	5 (7.7%)								
Respiratory comorbidity n (%)	0 (0.0%)	0 (0.0%)						176	111	65
Charlson index zero or one n (%)	101 (89.4%)	53 (81.5%)	0.172	Fisher				178	113	65
Charlson index of two or more n (%)	12 (10.6%)	12 (18.5%)								
WHO clinical progression scale n (%)										
WHO – class 3-4	20 (18.3%)	12 (18.5%)	0.105	Fisher				174	109	65
WHO – class 5	67 (61.5%)	30 (46.2%)								
WHO – class 6	16 (14.7%)	14 (21.5%)								
WHO – class 7-9	6 (5.5%)	9 (13.8%)								
Acute pulmonary injury n (%)	6 (5.5%)	4 (6.2%)	1.000	Fisher	1.27 (0.30, 4.88)	0.725	Logistic	174	109	65
Acute cardiac injury n (%)	10 (9.2%)	19 (29.2%)	**0.001**	X²	4.13 (1.77, 10.18)	**0.001**	Logistic	174	109	65
Acute liver injury n (%)	61 (59.8%)	34 (54.0%)	0.566	X²	0.83 (0.43, 1.60)	0.573	Logistic	165	102	63
Acute kidney injury n (%)	18 (16.5%)	9 (13.8%)	0.800	X²	0.75 (0.28, 1.85)	0.536	Logistic	174	109	65
Admission duration (days)	6.00 [3.00, 9.00]	8.0 [3.0, 13.0]	**0.048**	Mann-U	0.34 (0.03, 0.64)	**0.030**	Norm linear	174	109	65
Abnormal chest x-ray n (%)	8 (14.5%)	12 (32.4%)	0.075	X²	3.22 (1.09, 10.18)	**0.038**	Logistic	92	55	37
Treatments										
Proning n (%)	10 (11.1%)	13 (24.1%)	0.069	X²	2.63 (1.01, 7.03)	**0.049**	Logistic	144	90	54
Remdesivir n (%)	17 (29.3%)	5 (22.7%)	0.758	X²	0.66 (0.18, 2.08)	0.492	Logistic	80	58	22
Systemic (oral or IV) steroids n (%)	82 (75.2%)	44 (68.8%)	0.455	X²	0.74 (0.36, 1.52)	0.406	Logistic	173	109	64
Therapeutic dose anti-coagulation n (%)	59 (54.1%)	39 (60.0%)	0.550	X²	1.65 (0.84, 3.31)	0.149	Logistic	174	109	65
Non-steroidal anti-inflammatory (NSAID) n (%)	11 (10.1%)	7 (10.8%)	1.000	X²	0.93 (0.31, 2.57)	0.888	Logistic	174	109	65
Follow-up Lung function										
FEV1 (L)	3.01 (±0.85)	2.81 (±0.72)	0.182	T/Welch	-0.11 (-0.43, 0.21)	0.508	Norm linear	117	71	46
FEV1 < 80% of predicted n (%)	10 (18.9%)	9 (26.5%)	0.568	X²	1.62 (0.54, 4.85)	0.381	Logistic	87	53	34
FVC (L)	3.79 (±0.97)	3.48 (±0.96)	0.090	T/Welch	-0.22 (-0.56, 0.11)	0.185	Norm linear	117	71	46
FVC < 80% of predicted n (%)	7 (13.2%)	11 (32.4%)	0.060	X²	3.27 (1.07, 10.77)	**0.041**	Logistic	87	53	34
FEV1:FVC	0.80 [0.76, 0.83]	0.80 [0.78, 0.84]	0.106	Mann-U	0.32 (-0.04, 0.67)	0.081	Norm linear	117	71	46
Ratio FEV1:FVC less than 0.7 n (%)	6 (8.5%)	2 (4.3%)	0.478	Fisher	0.50 (0.06, 2.64)	0.451	Logistic	117	71	46
TLCO mmol/min/kPa (SI)	8.23 (±1.90)	7.16 (±1.82)	**0.038**	T/Welch	-0.50 (-0.97, -0.02)	**0.040**	Norm linear	59	37	22
KCO mmol/min/kPa (SI)	1.51 [1.37, 1.61]	1.46 [1.36, 1.61]	0.605	Mann-U	-0.11 (-0.57, 0.35)	0.631	Norm linear	59	37	22
Do you feel recovered? n (%)										
No	39 (40.6%)	39 (66.1%)	**0.001**	Fisher				155	96	59
Not sure	26 (27.1%)	4 (6.8%)								
Yes	31 (32.3%)	16 (27.1%)								

Reported outcomes										
Cough n (%)	21 (21.9%)	22 (37.9%)	**0.049**	X^2	2.35 (1.11, 5.02)	**0.026**	Logistic	154	96	58
Breathlessness n (%)	42 (43.8%)	33 (55.9%)	0.191	X^2	1.90 (0.96, 3.81)	0.068	Logistic	155	96	59
Chest pain n (%)	20 (20.4%)	10 (16.9%)	0.746	X^2	0.77 (0.30, 1.87)	0.570	Logistic	157	98	59
Chest tightness n (%)	21 (21.4%)	24 (40.7%)	**0.016**	X^2	3.27 (1.48, 7.51)	**0.004**	Logistic	157	98	59
Joint pain n (%)	37 (37.8%)	31 (52.5%)	0.100	X^2	1.82 (0.93, 3.61)	0.082	Logistic	157	98	59
Dyspnoea-12 score	1.00 [0.00, 5.00]	3.00 [0.00, 7.00]	0.252	Mann-U	0.28 (-0.05, 0.61)	0.093	Norm linear	147	86	61
FACIT V4 Score	38.0 (±11.7)	37.4 (±10.6)	0.768	T/Welch	-0.15 (-0.47, 0.18)	0.371	Norm linear	147	86	61
Anxiety (GAD-7 score)	2.00 [0.00, 7.00]	3.00 [0.00, 6.00]	0.986	Mann-U	0.05 (-0.27, 0.37)	0.763	Norm linear	148	87	61
Anxiety (GAD-7 >8) n (%)	16 (18.4%)	10 (16.4%)	0.924	X^2	1.11 (0.41, 3.01)	0.830	Logistic	148	87	61
Depression (PHQ-9) score	3.00 [0.00, 9.75]	5.50 [2.00, 9.00]	0.146	Mann-U	0.30 (-0.02, 0.62)	0.069	Norm linear	146	86	60
Depression (PHQ-9 >= 10) n (%)	22 (25.6%)	13 (21.7%)	0.728	X^2	1.04 (0.44, 2.43)	0.930	Logistic	146	86	60
EQ5D-5L utility score	79.5 [70.8, 100.0]	76.8 [67.2, 87.9]	0.173	Mann-U	-0.24 (-0.57, 0.08)	0.141	Norm linear	151	92	59
EQ5D-5L less than 60%, n (%)	10 (10.9%)	10 (16.9%)	0.407	X^2	2.11 (0.74, 6.13)	0.161	Logistic	151	92	59

Abn N number of cases with abnormal lung MRI, BMI body mass index, BNP B-type natriuretic peptide, C-reactive protein, EQ5D-5L quality of life, GFR glomerular filtration rate, IV intravenous, KCO carbon monoxide transfer co-efficient, FEV1 forced expiratory volume in 1 second, FVC forced vital capacity, No-Abn N number of cases without abnormal liung MRI, NT N-terminal, cT1nI troponin I, MOCA montreal cognitive assessment, GAD-7 generalised anxiety disorder, PHQ-9 personal health questionnaire, PHOSP-COVID cluster PHOSP-COVID study symptom cluster, SQR square, TLCO transfer capacity of lung, ULN upper limit of normal. For Part A, inverse probability weighting was used to adjust imaging variable for confounders which included age, sex, body mass index, smoking, hypertension, hypercholesterolemia, diabetes, cardiac, brain, liver, lung and renal comorbidities, and scanner manufacturer. Part B and C (which excludes corresponding organ comorbidities) regression models were adjusted for age, sex, smoking, hypertension, diabetes, Charlson comorbidity index, obesity and scanner manufacturer. *Confidence intervals for age difference are not adjusted.

Supplementary Table. 2. Comparison of quantitative multiorgan MRI metrics across patients with and without an abnormal C-reactive protein at follow-up visit (median 5 months).

Variable	Follow-up CRP < 5mg/L	Follow-up CRP ≥5mg/L	CRP< 5 mg/L (n)	CRP≥5 mg/L	no CRP available	Regression coefficient	Multivariate model P- Value
Left ventricular indexed stroke volume (ml/m2)	44·1 (±7·7)	41·1 (±6·5)	221	44	40	-0·377	**0·024**
Right ventricular indexed stroke volume ml/m2)	43·4 (±7·8)	40·4 (±6·3)	219	44	40	-0·351	**0·036**
Liver cT1 (ms)	759·5 [711·2, 818·2]	823·0 [767·0, 902·0]	200	43	35	0·387	**0·02**
Lung T2 HASTE heterogeneity (coefficient of variance)	46·2 (±5·1)	51·6 (±6·1)	142	30	38	0·574	**0·003**
Right superior temporal gyrus grey matter (ml)	3,147 [2,721, 3,635]	3,106 [2,828, 3,949]	180	37	31	0·406	**0·008**
Right middle temporal gyrus grey matter (ml)	1,669 (±352)	1,537 (±356)	180	37	31	-0·332	**0·04**
Left middle temporal gyrus grey matter (ml)	3,478 [2,981, 4,098]	3,628 [3,071, 4,150]	180	37	31	0·326	**0·045**
Left inferotemporal gyrus involving temporo-occipital regional grey matter (ml)	3,077 (±649)	3,331 (±654)	180	37	31	0·536	**0·001**
Left frontal operculum grey matter (ml)	1,480 [1,349, 1,699]	1,633 [1,389, 1,811]	180	37	31	0·332	**0·042**
Left pallidum grey matter (ml)	23·5 [16·9, 33·6]	16·6 [10·8, 27·1]	180	37	31	-0·438	**0·01**
Brainstem grey matter (ml)	4,887 [4,407, 5,394]	5,298 [4,452, 5,826]	180	37	31	0·457	**0·004**
Amydala: Amygdala in the right hemisphere generated by subcortical volumetric segmentation (aseg) (ml)	1,749 (±235)	1,659 (±219)	191	39	33	-0·328	**0·034**
Amydala: Accessory-Basal-nucleus in the left hemisphere (ml)	247·6 (±33·2)	237·4 (±32·1)	191	39	33	-0·296	**0·048**
Amydala: Corticoamygdaloid-transitio in the left hemisphere (ml)	170·4 (±25·0)	163·6 (±25·7)	191	39	33	-0·311	**0·05**
Amydala: Whole-amygdala in the left hemisphere (ml)	1,666 (±194)	1,595 (±203)	191	39	33	-0·29	**0·05**
Amydala: Accessory-Basal-nucleus in the right hemisphere (ml)	260·9 (±35·0)	247·0 (±26·7)	191	39	33	-0·336	**0·031**
Amydala: Cortical-nucleus in the right hemisphere (ml)	28·4 (±5·4)	26·4 (±3·9)	191	39	33	-0·434	**0·011**
Amygdala: Corticoamygdaloid-transitio in the right hemisphere (ml)	176·4 (±26·5)	166·2 (±22·8)	191	39	33	-0·408	**0·01**
Thalamus: MGN in the left hemisphere (ml)	115·2 [107·4, 128·5]	109·6 [99·5, 115·5]	191	39	33	-0·38	**0·024**
Thalamus: CM in the left hemisphere (ml)	240·5 [219·6, 261·6]	218·3 [202·8, 246·6]	191	39	33	-0·32	**0·05**
Bankssts in the right hemisphere (ml)	2,355 (±380)	2,418 (±412)	191	39	33	0·373	**0·021**
Pericalcarine in the right hemisphere (ml)	2,377 (±476)	2,167 (±435)	191	39	33	-0·491	**0·005**
G-cingul-Post-dorsal in the left hemisphere (ml)	1,791 (±330)	1,653 (±273)	191	39	33	-0·336	**0·042**
G-subcallosal in the left hemisphere (ml)	1,271 (±358)	1,050 (±328)	191	39	33	-0·426	**0·012**
G-cuneus in the right hemisphere (ml)	3,058 (±517)	2,863 (±426)	191	39	33	-0·331	**0·049**
G-temp-sup-G-T-transv in the right hemisphere (ml)	898·5 (±180·2)	815·7 (±150·0)	191	39	33	-0·434	**0·011**
G-temp-sup-Lateral in the right hemisphere (ml)	5,247 (±712)	5,376 (±865)	191	39	33	0·334	**0·038**

C-reactive protein, HASTE half-Fourier acquisition single-shot turbo spin-echo, cT1 Iron corrected T1

Supplementary Table 3. A) Comparison of heart MRI in patients vs controls; B) Clinical characteristics of patients with and without heart MRI abnormalities; C) Clinical characteristics of patients with and without heart MRI abnormalities after excluding pre-existing cardiac conditions.

Part A: Comparison of cardiac health between patients vs controls

					Multivariate analysis - adjusted inverse probability weighting (IPW)		Multivariate analysis - adjusted with IPW and excluding patients with WHO >= 7		
**Variable**	**Controls (n= 52)**	**Patients (n= 259)**	**P-value**	**Univariate test**	**Odds Ratio or Beta coefficient**	**p-value**	**Odds Ratio or Beta coefficient**	**p-value**	**Test**
Cardiac	2 (3.8%)	40 (15.6%)	**0.043**	X²	1.67 (0.66, 5.19)	0.321	1.67 (0.65, 5.20)	0.326	Logistic
Diabetes	7 (13.5%)	55 (21.4%)	0.265	X²	0.57 (0.28, 1.19)	0.125	0.58 (0.28, 1.22)	0.139	Logistic
Hypertension	15 (28.8%)	128 (50.4%)	**0.007**	X²	0.88 (0.46, 1.66)	0.684	0.86 (0.45, 1.64)	0.649	Logistic
High cholesterol	7 (13.5%)	46 (17.9%)	0.567	X²	0.79 (0.37, 1.81)	0.551	0.75 (0.35, 1.75)	0.490	Logistic
Acute cardiac injury		38 (14.9%)							
Either BNP > 1x limit		21 (36.8%)							
Follow-up any BNP > 1x	6 (12.5%)	32 (18.4%)	0.458	X²	1.13 (0.47, 3.00)	0.792	1.08 (0.44, 2.90)	0.873	Logistic
Any troponin > 1x limit		24 (17.5%)							
Follow-up any troponin > 1x		5 (3.6%)	0.329	Fisher					
**Cardiac MRI metrics**									
**Cardiac abnormalities**	12 (24.5%)	54 (20.8%)	0.704	X²	0.68 (0.33, 1.45)	0.297	0.69 (0.33, 1.49)	0.332	Logistic
Quantitative cardiac MRI abnormalities									
LVEF < 52%	1 (2.0%)	18 (7.0%)	0.330	Fisher	4.55 (0.68, 237.04)	0.237	3.87 (0.56, 202.96)	0.294	Logistic
RVEF < 48%	1 (2.0%)	11 (4.3%)	0.698	Fisher	2.61 (0.36, 138.48)	0.461	2.50 (0.34, 133.62)	0.483	Logistic
Left ventricular end diastolic volume index (ml/m2)	75.0 (±12.9)	71.7 (±13.1)	0.067	Mann-U	-0.18 (-0.50, 0.14)	0.281	-0.18 (-0.50, 0.14)	0.266	Linear
Left ventricular end systolic volume index (ml/m2)	28.1 (±7.0)	28.6 (±8.1)	0.891	Mann-U	0.05 (-0.27, 0.37)	0.747	0.02 (-0.30, 0.34)	0.893	Linear
left ventricular stroke volume index (ml/m2)	46.9 (±8.1)	43.1 (±7.4)	**0.003**	Mann-U	-0.35 (-0.66, -0.03)	**0.032**	-0.32 (-0.63, 0.00)	0.050	Linear
Left ventricular mass index (g/m2)	54.3 (±9.1)	53.2 (±10.0)	0.408	Mann-U	-0.26 (-0.59, 0.06)	0.113	-0.28 (-0.61, 0.04)	0.085	Linear
Right ventricular end diastolic volume index (ml/m2)	79.6 (±15.9)	71.4 (±14.4)	**<0.001**	Mann-U	-0.44 (-0.76, -0.12)	**0.007**	-0.45 (-0.77, -0.13)	**0.006**	Linear
Right ventricular end systolic volume index (ml/m2)	33.0 (±9.6)	29.1 (±9.4)	**0.003**	Mann-U	-0.38 (-0.71, -0.06)	**0.021**	-0.42 (-0.75, -0.09)	**0.012**	Linear
Right ventricular stroke volume index (ml/m2)	46.6 (±8.4)	42.3 (±7.3)	**<0.001**	Mann-U	-0.43 (-0.75, -0.11)	**0.008**	-0.41 (-0.72, -0.09)	**0.012**	Linear
Left ventricular mass (g)	108.5 (±27.1)	109.4 (±27.1)	0.827	Mann-U	-0.17 (-0.49, 0.16)	0.315	-0.18 (-0.50, 0.15)	0.285	Linear
Left ventricular ejection fraction (%)	62.9 (±5.5)	60.5 (±6.0)	**0.015**	Mann-U	-0.32 (-0.64, -0.00)	**0.048**	-0.28 (-0.60, 0.04)	0.088	Linear
Left ventricular cardiac output (ml/min)	6.38 (±1.60)	6.02 (±1.50)	0.122	Mann-U	-0.21 (-0.54, 0.13)	0.229	-0.18 (-0.52, 0.15)	0.280	Linear
Right ventricular end diastolic volume (ml)	158.1 (±40.5)	146.8 (±38.6)	0.064	Mann-U	-0.32 (-0.64, 0.01)	0.055	-0.32 (-0.65, 0.00)	0.050	Linear
Right ventricular end systolic volume (ml)	65.7 (±22.4)	60.2 (±22.9)	0.080	Mann-U	-0.29 (-0.62, 0.04)	0.080	-0.32 (-0.65, 0.01)	0.056	Linear
Right ventricular stroke volume (ml)	92.4 (±21.3)	86.6 (±19.5)	0.092	Mann-U	-0.28 (-0.60, 0.04)	0.084	-0.26 (-0.58, 0.06)	0.116	Linear
Right ventricular ejection fraction (%)	59.1 (±6.1)	59.9 (±6.7)	0.347	Mann-U	0.15 (-0.17, 0.48)	0.360	0.21 (-0.12, 0.54)	0.213	Linear
Right ventricular cardiac output	6.33 (±1.63)	5.90 (±1.46)	0.078	Mann-U	-0.21 (-0.55, 0.12)	0.211	-0.20 (-0.53, 0.14)	0.247	Linear
Average extra-cellular volume	0.29 (±0.03)	0.28 (±0.03)	0.200	Mann-U	-0.33 (-0.69, 0.04)	0.078	-0.32 (-0.68, 0.05)	0.086	Linear
Average T1 normalised Z score	0.02 (±1.92)	-0.00672 (±1.75)	0.879	Mann-U	-0.21 (-0.54, 0.12)	0.211	-0.19 (-0.52, 0.14)	0.258	Linear
Average T2 normalised Z score	0.71 [0.11, 1.74]	0.61 [-0.07, 1.25]	0.162	Mann-U	-0.50 (-0.81, -0.19)	-	-0.50 (-0.81, -0.19)	-	Linear
Qualitative cardiac MRI analysis									

LGE pathological	6 (12.2%)	34 (13.5%)	0.988	X²	0.71 (0.31, 1.72)	0.418	0.73 (0.32, 1.78)	0.462	Logistic
LGE probable myocarditis	6 (12.2%)	22 (8.8%)	0.426	Fisher	0.41 (0.17, 1.04)	0.050	0.41 (0.17, 1.04)	0.050	Logistic
LGE ischaemic	1 (2.0%)	14 (5.6%)	0.479	Fisher	1.21 (0.34, 6.59)	0.795	1.32 (0.37, 7.24)	0.698	Logistic
LGE mixed	0 (0.0%)	2 (0.8%)	1.000	Fisher					
Pericardial effusion	1 (2.0%)	3 (1.2%)	0.512	Fisher					

Part B: Clinical characteristics of patients with and without cardiac MRI abnormalities

Variable	No cardiac abnormalities (n= 205)	Cardiac abnormalities (n= 54)	P-value	Univariate test	Odds Ratio or Beta coefficient	p-value	Test	Total N	No-abn N	Abn N
Age (years)	55·6 (±12·0)	62·4 (±12·0)	**<0.001**	T/Welch	6.82 yrs [3.22, 10.42]*			259	205	54
Female n (%)	84 (41·0%)	17 (31·5%)						259	205	54
Non-white ethnicity n (%)	57 (27·8%)	15 (27·8%)	1.000	X²	1·25 (0·58, 2·61)	0.562	Logistic	259	205	54
Pre-existing comorbidities										
Obesity n (%)	101 (49·3%)	28 (51·9%)	0.853	X²	1·20 (0·64, 2·25)	0.566	Logistic	259	205	54
Cardiac n (%)	22 (10·8%)	18 (34·0%)	**<0.001**	X²	3·16 (1·39, 7·18)	**0.006**	Logistic	257	204	53
Diabetes n (%)	46 (22·5%)	9 (17·0%)	0.489	X²	0·45 (0·15, 1·15)	0.116	Logistic	257	204	53
Hypercholestrolemia n (%)	34 (16·7%)	12 (22·6%)	0.418	X²	1·05 (0·42, 2·50)	0.915	Logistic	257	204	53
Hypertension n (%)	96 (47·8%)	32 (60·4%)	0.139	X²	1·10 (0·56, 2·18)	0.782	Logistic	254	201	53
Charlson index of zero or one n (%)	174 (84·9%)	41 (75·9%)	0.152	Fisher				259	205	54
Charlson index of two or more n (%)	31 (15·1%)	13 (24·1%)								
WHO clinical progress scale n (%)										
WHO – class 3-4	36 (17·8%)	9 (17·0%)	0.994	Fisher				255	202	53
WHO – class 5	112 (55·4%)	29 (54·7%)								
WHO – class 6	40 (19·8%)	11 (20·8%)								
WHO – class 7-9	14 (6·9%)	4 (7·5%)								
Admission duration (days)	6·00 [3·00, 10·00]	6·0 [3·0, 10·0]	0.898	Mann-U	-0·11 (-0·42, 0·20)	0.486	Linear	255	202	53
Acute cardiac injury n (%)	27 (13.4%)	11 (20.8%)	0.2595	X²	1.56 (0.66, 3.49)	0.289	Logistic	255	202	53
Abnormal chest x-ray n (%)	19 (18·8%)	18 (54·5%)	**<0.001**	X²	4·95 (1·88, 13·50)	**0.001**	Logistic	134	101	33
Acute biochemistry										
Acute D-dimer > 500 u n (%)	91 (68·4%)	26 (86·7%)	0.075	X²	3·83 (1·24, 14·88)	**0.031**	Logistic	163	133	30
Acute kidney injury n (%)	27 (13·4%)	15 (28·3%)	**0.016**	X²	1·53 (0·65, 3·45)	0.312	Logistic	255	202	53
Acute eGFR (ml/min per 1.73m2)	86·0 [70·2, 90·0]	75·0 [58·0, 89·0]	**0.007**	Mann-U	-0·15 (-0·43, 0·13)	0.280	Linear	251	198	53
Acute eGFR < 60ml/min per 1.73m2 n (%)	26 (13·1%)	15 (28·3%)	**0.015**	X²	1·59 (0·68, 3·62)	0.273	Logistic	251	198	53
Treatments										
Remdesivir n (%)	24 (26·7%)	5 (20·0%)	0.675	X²	0·41 (0·11, 1·36)	0.165	Logistic	115	90	25
Systemic (oral or IV) steroids n (%)	149 (74·1%)	42 (79·2%)	0.556	X²	1·33 (0·63, 2·97)	0.470	Logistic	254	201	53
Therapeutic dose anti-coagulation n (%)	107 (53·0%)	25 (47·2%)	0.550	X²	0·87 (0·45, 1·68)	0.682	Logistic	255	202	53
Non-steroidal anti-inflammatory (NSAID) n (%)	21 (10·4%)	7 (13·2%)	0.737	X²	1·03 (0·36, 2·60)	0.958	Logistic	255	202	53
Follow-up lung function										
Forced vital capacity (L)	3·59 (±0·97)	3·67 (±1·01)	0.636	T/Welch	0·12 (-0·19, 0·42)	0.452	Linear	174	134	40
Forced expiratory volume in 1 second (L/s)	2·82 (±0·80)	2·79 (±0·88)	0.819	T/Welch	0·05 (-0·25, 0·34)	0.764	Linear	174	134	40
Ratio of FEV1 and FVC	0·79 (±0·09)	0·76 (±0·12)	0.099	T/Welch	-0·38 (-0·70, -0·05)	**0.026**	Linear	174	134	40
KCO mmol/min/kPa (SI)	1·46 [1·31, 1·61]	1·41 [1·20, 1·47]	0.120	Mann-U	-0·43 (-0·86, -0·01)	**0.044**	Linear	81	66	15
Biochemistry										
Follow-up cardiac injury n (%)	20 (12·8%)	17 (37·0%)	**<0.001**	X²	3·14 (1·35, 7·24)	**0.007**	Logistic	202	156	46
Follow-up Pro-NT BNP	43·0 [35·0, 77·5]	76·5 [38·2, 227·2]	**0.003**	Mann-U	0·61 (0·25, 0·97)	**0.001**	Linear	157	127	30
Follow-up any BNP > 1x n (%)	17 (12·6%)	15 (38·5%)	**<0.001**	X²	3·50 (1·40, 8·77)	**0.007**	Logistic	174	135	39

Follow-up high sensitivity troponin	2·50 [2·00, 3·20]	3·50 [2·00, 7·20]	**0.043**	Mann-U	0·12 (-0·29, 0·53)	0.568	Norm linear	106	77	29
Follow-up eGFR (ml/min per 1.73m2)	90·0 [79·0, 90·0]	82·0 [68·0, 90·0]	**0.004**	Mann-U	-0·29 (-0·58, -0·00)	**0.049**	Linear	220	173	47
Serum creatinine (μmol/L)	74·0 [63·0, 84·0]	80·0 [71·0, 96·0]	**0.007**	Mann-U	0·27 (-0·00, 0·55)	0.051	Linear	224	175	49
Serum creatinine (>104 men, >84 women) n (%)	14 (8·0%)	11 (22·4%)	**0.010**	X²	3·12 (1·20, 8·03)	**0.018**	Logistic	224	175	49
Do you feel recovered?										
No	95 (51·1%)	26 (54·2%)	0.954	Fisher				234	186	48
Not sure	39 (21·0%)	10 (20·8%)								
Yes	52 (28·0%)	12 (25·0%)								
Reported outcomes										
Chest pain n (%)	41 (21·9%)	9 (18·4%)	0.729	X²	0·96 (0·39, 2·18)	0.932	Logistic	236	187	49
Chest tightness n (%)	64 (34·4%)	15 (30·6%)	0.741	X²	1·09 (0·52, 2·26)	0.810	Logistic	235	186	49
Palpitations n (%)	52 (28·0%)	11 (22·9%)	0.604	X²	0·85 (0·38, 1·78)	0.671	Logistic	234	186	48
Dizziness or light headedness n (%)	60 (32·1%)	12 (25·0%)	0.439	X²	0·79 (0·37, 1·63)	0.537	Logistic	235	187	48
Breathlessness n (%)	97 (52·7%)	25 (52·1%)	1.000	X²	0·96 (0·50, 1·87)	0.907	Logistic	232	184	48
Fatigue n (%)	116 (63·0%)	30 (63·8%)	1.000	X²	1·11 (0·56, 2·27)	0.767	Logistic	231	184	47
Dyspnoea-12 score	3·00 [0·00, 10·00]	4·50 [0·00, 9·00]	0.443	Mann-U	0·15 (-0·18, 0·47)	0.385	Linear	224	178	46
FACIT fatigue score	35·4 (±11·9)	36·7 (±11·8)	0.498	T/Welch	-0·01 (-0·33, 0·30)	0.938	Linear	224	178	46
Anxiety (GAD-7 score)	3·50 [0·00, 8·00]	2·50 [0·00, 8·00]	0.657	Mann-U	0·13 (-0·18, 0·45)	0.413	Norm linear	226	180	46
Anxiety (GAD-7>8) n (%)	38 (21·1%)	9 (19·6%)	0.978	X²	1·39 (0·55, 3·31)	0.468	Logistic	226	180	46
Depression (PHQ-9) score	6·92 (±6·46)	6·67 (±6·18)	0.811	T/Welch	0·13 (-0·19, 0·45)	0.431	Norm linear	224	179	45
Depression (PHQ-9 >= 10) n (%)	54 (30·2%)	14 (31·1%)	1.000	X²	1·48 (0·67, 3·21)	0.318	Logistic	224	179	45
EQ-5D-5L utility	76·7 [65·7, 90·6]	76·8 [67·9, 87·9]	0.560	Mann-U	0·01 (-0·32, 0·33)	0.963	Linear	226	181	45
EQ-5D-5L less than 60% n (%)	33 (18·2%)	6 (13·3%)	0.577	X²	0·81 (0·28, 2·08)	0.684	Logistic	226	181	45

Part C: Clinical characteristics of patients with and without cardiac MRI abnormalities after excluding pre-existing cardiac conditions

Variable	No cardiac abnormalities (n= 183)	Cardiac abnormalities (n= 36)	P-value	Univariate test	Odds Ratio or Beta coefficient	p-value	Test	Total N	No-abn N	Abn N
Age (years)	54.8 (±11.8)	59.8 (±11.9)	**0.027**	T/Welch	4.67 yrs [0.36, 8.98]*			219	183	36
Female n (%)	73 (39.9%)	12 (33.3%)						219	183	36
Non-white ethnicity n (%)	53 (29.0%)	6 (16.7%)	0.189	X²	0.52 (0.17, 1.34)	0.196	Logistic	219	183	36
Pre-existing comorbidities										
Obesity n (%)	90 (49.2%)	18 (50.0%)	1.000	X²	1.08 (0.52, 2.27)	0.830	Logistic	219	183	36
Cardiac n (%)	0 (0.0%)	0 (0.0%)						217	182	35
Diabetes n (%)	41 (22.5%)	5 (14.3%)	0.386	X²	0.48 (0.13, 1.45)	0.224	Logistic	217	182	35
High cholesterol n (%)	27 (14.8%)	6 (17.1%)	0.927	X²	0.91 (0.28, 2.67)	0.873	Logistic	217	182	35
Hypertension n (%)	79 (44.1%)	20 (57.1%)	0.220	X²	1.38 (0.63, 3.05)	0.416	Logistic	214	179	35
Charlson index zero or one n (%)	160 (87.4%)	31 (86.1%)	0.788	Fisher				219	183	36
Charlson index of two or more n (%)	23 (12.6%)	5 (13.9%)								
WHO clinical progression scale										
WHO – class 3-4	32 (17.8%)	4 (11.4%)	0.623	Fisher				215	180	35
WHO – class 5	100 (55.6%)	23 (65.7%)								
WHO – class 6	36 (20.0%)	5 (14.3%)								
WHO – class 7-9	12 (6.7%)	3 (8.6%)								
Admission duration (days)	6.00 [3.00, 10.00]	6.0 [3.0, 10.0]	0.989	Mann-U	-0.07 (-0.44, 0.29)	0.705	Linear	215	180	35
Acute cardiac injury n (%)	25 (13.9%)	4 (11.4%)	1.000	Fisher	0.78 (0.21, 2.25)	0.666	Logistic	215	180	35
Abnormal chest x-ray n (%)	15 (17.2%)	10 (52.6%)	**0.002**	Fisher	6.24 (1.92, 21.70)	**0.003**	Logistic	106	87	19
Acute biochemistry										
Acute D-dimer (u/L)	700 [408, 1299]	881.5 [617.5, 1750]	0.157	Mann-U	0.43 (-0.06, 0.91)	0.085	Linear	145	125	20
Acute D-dimer > 500 u/L	84 (67.2%)	18 (90.0%)	0.070	X²	6.25 (1.57, 42.55)	**0.023**	Logistic	145	125	20
Acute kidney injury n (%)	19 (10.6%)	8 (22.9%)	0.054	Fisher	1.93 (0.67, 5.18)	0.203	Logistic	215	180	35

Acute eGFR (ml/min/1.73 m^2)	87.5 [72.0, 90.0]	85.0 [60.5, 90.0]	0.096	Mann-U	-0.14 (-0.48, 0.20)	0.422	Linear	211	176	35
Acute eGFR < 60	18 (10.2%)	8 (22.9%)	**0.049**	Fisher	2.03 (0.70, 5.52)	0.175	Logistic	211	176	35
Treatments										
Remdesivir n (%)	22 (27.5%)	3 (18.8%)	0.550	Fisher	0.33 (0.06, 1.37)	0.155	Logistic	96	80	16
Systemic (oral or IV) steroids n (%)	134 (74.9%)	30 (85.7%)	0.242	X²	1.90 (0.73, 5.93)	0.222	Logistic	214	179	35
Therapeutic dose anti-coagulation n (%)	98 (54.4%)	15 (42.9%)	0.284	X²	0.59 (0.26, 1.29)	0.187	Logistic	215	180	35
Non-steroidal anti-inflammatory (NSAID) n (%)	19 (10.6%)	2 (5.7%)	0.540	Fisher	0.46 (0.07, 1.78)	0.326	Logistic	215	180	35
Follow-up lung function										
Forced vital capacity (L)	3.64 (±0.93)	3.90 (±0.96)	0.196	T/Welch	0.31 (-0.04, 0.66)	0.081	Linear	148	120	28
Forced expiratory volume in 1 second (L/s)	2.87 (±0.78)	3.03 (±0.81)	0.334	T/Welch	0.25 (-0.10, 0.59)	0.157	Linear	148	120	28
Ratio of FEV1 and FVC	0.79 (±0.09)	0.78 (±0.11)	0.736	T/Welch	-0.25 (-0.65, 0.15)	0.213	Linear	148	120	28
KCO mmol/min/kPa (SI)	1.48 [1.39, 1.63]	1.41 [1.41, 1.59]	0.717	Mann-U	-0.18 (-0.76, 0.40)	0.534	Linear	68	59	9
Biochemistry										
Follow-up cardiac injury	15 (10.9%)	7 (23.3%)	0.078	Fisher	2.58 (0.83, 7.62)	0.089	Logistic	168	138	30
Follow-up Pro-NT BNP	40.5 [34.0, 76.5]	50.0 [35.0, 89.2]	0.255	Mann-U	0.34 (-0.10, 0.79)	0.132	Linear	132	112	20
Follow-up any BNP > 1x	12 (10.2%)	6 (24.0%)	0.090	Fisher	2.93 (0.85, 9.64)	0.079	Logistic	143	118	25
Follow-up hs-cTnI	2.50 [2.00, 3.20]	2.00 [2.00, 4.50]	0.868	Mann-U	-0.21 (-0.71, 0.30)	0.419	Linear	87	69	18
Follow-up eGFR (ml/min/1.73 m^2)	90.0 [81.0, 90.0]	87.0 [71.0, 90.0]	**0.031**	Mann-U	-0.33 (-0.69, 0.02)	0.065	Linear	184	153	31
Follow-up D-dimer (u/L)	205.0 [145.0, 297.2]	270.0 [195.0, 353.0]	**0.049**	Mann-U	0.30 (-0.10, 0.70)	0.145	Linear	161	132	29
Follow-up D-dimer > 500 u/L	9 (6.8%)	6 (20.7%)	**0.031**	Fisher	3.31 (0.94, 11.04)	0.052	Logistic	161	132	29
Serum creatinine (umol/L)	73.5 (±15.0)	81.5 (±20.0)	**0.039**	T/Welch	0.28 (-0.05, 0.60)	0.092	Linear	186	154	32
Serum creatinine (>104 for men, >84 for women)	9 (5.8%)	7 (21.9%)	**0.009**	Fisher	5.61 (1.70, 18.73)	**0.004**	Logistic	186	154	32
Do you feel recovered?										
No	86 (51.8%)	20 (62.5%)	0.451	Fisher				198	166	32
Not sure	36 (21.7%)	4 (12.5%)								
Yes	44 (26.5%)	8 (25.0%)								
Reported outcomes										
Chest pain n (%)	35 (21.0%)	6 (18.8%)	0.965	X²	0.87 (0.29, 2.25)	0.780	Logistic	199	167	32
Chest tightness n (%)	57 (34.3%)	11 (34.4%)	1.000	X²	1.10 (0.47, 2.51)	0.820	Logistic	198	166	32
Palpitations n (%)	42 (25.3%)	6 (18.8%)	0.571	X²	0.71 (0.25, 1.78)	0.491	Logistic	198	166	32
Dizziness or light headedness n (%)	52 (31.1%)	7 (21.9%)	0.401	X²	0.69 (0.26, 1.65)	0.424	Logistic	199	167	32
Breathlessness n (%)	85 (51.8%)	18 (56.2%)	0.791	X²	1.11 (0.51, 2.47)	0.789	Logistic	196	164	32
Fatigue n (%)	102 (62.2%)	22 (71.0%)	0.467	X²	1.50 (0.65, 3.68)	0.355	Logistic	195	164	31
Dyspnoea-12 score	3.00 [0.00, 10.00]	4.00 [0.00, 6.75]	0.989	Mann-U	-0.08 (-0.47, 0.31)	0.689	Linear	193	163	30
FACIT fatigue score	35.5 (±11.8)	39.6 (±10.1)	0.053	T/Welch	0.28 (-0.09, 0.65)	0.143	Linear	193	163	30
Anxiety (GAD-7 score)	3.00 [0.00, 7.00]	1.50 [0.00, 7.50]	0.401	Mann-U	-0.02 (-0.39, 0.36)	0.926	Linear	194	164	30
Anxiety (GAD7 >8)	31 (18.9%)	5 (16.7%)	0.973	X²	1.27 (0.37, 3.75)	0.683	Logistic	194	164	30
Depression (PHQ-9) score	6.67 (±6.21)	5.90 (±5.90)	0.523	T/Welch	-0.03 (-0.41, 0.35)	0.882	Linear	192	163	29
Depression (PHQ-9 >= 10)	46 (28.2%)	8 (27.6%)	1.000	X²	1.38 (0.50, 3.61)	0.518	Logistic	192	163	29
EQ5D-5L utility	76.7 [65.9, 90.6]	83.7 [73.1, 100.0]	0.085	Mann-U	0.32 (-0.05, 0.70)	0.090	Linear	192	162	30
EQ5D-5L less than 60%	30 (18.5%)	2 (6.7%)	0.182	X²	0.36 (0.05, 1.35)	0.187	Logistic	192	162	30

Abn N number of cases with abnormal cardiac MRI, BMI body mass index, BNP B-type natriuretic peptide, C-reactive protein, EQ-5D-5L quality of life, FACIT Functional Assessment of Chronic Illness Therapy, GAD-7 generalised anxiety disorder, GFR glomerular filtration rate, IV intravenous, KCO carbon monoxide transfer co-efficient, FEV1 forced expiratory volume in 1 second, FVC forced vital capacity, No-Abn N number of cases without abnormal cardiac MRI, NT N-terminal, cT1nI troponin I,PHQ-9 personal health questionnaire, SQR square, TLCO transfer capacity of lung, ULN upper limit of normal, WHO world health organisation. For Part A, inverse probability weighting was used to adjust imaging variable for confounders which included age, sex, body mass index, smoking, hypertension, hypercholesterolemia, diabetes, cardiac, brain, liver, lung and renal comorbidities, and scanner manufacturer. Part B and C (excludes corresponding organ comorbidities), regression models were adjusted for age, sex, smoking, hypertension, diabetes, Charlson comorbidity index, obesity and scanner manufacturer.

Supplementary Table 4. A) Comparison of brain MRI in patients vs controls; B) Clinical characteristics of patients with and without brain MRI abnormalities; C) Clinical characteristics of patients with and without brain MRI abnormalities after excluding pre-existing neurological conditions.

Part A: Comparison of brain health between patients and controls

					Multivariate analysis - adjusted inverse probability weighting (IPW)		Multivariate analysis - adjusted with IPW and excluding patients with WHO >= 7		
**Variable**	**Controls (n= 52)**	**Patients (n= 259)**	**P-value**	**Univariate test**	**Odds Ratio or Beta coefficient**	**p-value**	**Odds Ratio or Beta coefficient**	**p-value**	**Test**
Pre-existing neurological comorbidity	1 (1.9%)	9 (3.5%)	1.000	Fisher	0.54 (0.13, 3.12)	0.433	0.59 (0.14, 3.40)	0.502	Logistic
**Brain MRI metrics**									
**Brain abnormalities**	9 (18.0%)	109 (50.0%)	**<0.001**	X²	3.47 (1.60, 8.34)	**0.003**	3.32 (1.52, 8.01)	**0.004**	Logistic
White matter hyperintensities	8 (16.0%)	100 (47.8%)	**<0.001**	X²	3.54 (1.59, 8.88)	**0.004**	3.52 (1.57, 8.88)	**0.004**	Logistic
Small vessel disease	7 (14.0%)	94 (44.8%)	**<0.001**	X²	3.59 (1.54, 9.73)	**0.006**	3.50 (1.49, 9.55)	**0.007**	Logistic
Total brain volume (ml)	1,569 (±74)	1,534 (±78)	**0.006**	Mann-U	-0.49 (-0.90, -0.08)	**0.019**	-0.46 (-0.87, -0.04)	**0.032**	Linear
Total brain white matter volume (ml)	754.3 (±32.5)	752.4 (±42.9)	0.661	Mann-U	-0.00 (-0.41, 0.40)	0.990	0.10 (-0.31, 0.50)	0.647	Linear
Total brain grey matter volume (ml)	814.3 (±52.3)	781.7 (±57.0)	**<0.001**	Mann-U	-0.69 (-1.08, -0.30)	**<0.001**	-0.70 (-1.10, -0.31)	**<0.001**	Linear
Brain white matter hyperintensity volume (ml)	1.63 [0.84, 2.56]	2.71 [1.54, 4.96]	**<0.001**	Mann-U	0.27 (-0.14, 0.68)	0.201	0.23 (-0.19, 0.64)	0.288	Linear
Grey matter parcellation									
Left putamen (mm3)	5,164 [4,755, 5,508]	4,725 [4,314, 5,080]	**<0.001**	Mann-U	-0.63 (-0.98, -0.27)	**<0.001**	-0.63 (-0.99, -0.27)	**<0.001**	Linear
Right putamen (mm3)	5,088 (±565)	4,741 (±595)	**<0.001**	Mann-U	-0.47 (-0.83, -0.11)	**0.011**	-0.48 (-0.85, -0.11)	**0.011**	Linear
Left middle temporal gyrus (mm3)	2,033 (±401)	1,784 (±482)	**<0.001**	Mann-U	-0.62 (-1.01, -0.23)	**0.002**	-0.63 (-1.02, -0.24)	**0.002**	Linear
Right middle temporal gyrus (mm3)	5,962 [5,307, 6,531]	5,342 [4,844, 5,976]	**<0.001**	Mann-U	-0.62 (-0.99, -0.26)	**<0.001**	-0.59 (-0.96, -0.23)	**0.002**	Linear
Left intracalcarine grey matter (mm3)	2,335 (±649)	2,166 (±619)	0.114	Mann-U	-0.56 (-0.92, -0.19)	**0.003**	-0.58 (-0.95, -0.21)	**0.002**	Linear
Right intracalcarine grey matter (mm3)	2,407 (±591)	2,221 (±588)	0.062	Mann-U	-0.62 (-0.98, -0.26)	**<0.001**	-0.67 (-1.04, -0.30)	**<0.001**	Linear
Left cuneal grey matter (mm3)	1,993 [1,603, 2,274]	1,784 [1,494, 2,036]	**0.007**	Mann-U	-0.51 (-0.88, -0.13)	**0.009**	-0.51 (-0.89, -0.13)	**0.008**	Linear
Right cuneal grey matter (mm3)	2,389 [2,018, 2,832]	2,194 [1,922, 2,494]	**0.045**	Mann-U	-0.33 (-0.70, 0.03)	0.075	-0.40 (-0.76, -0.03)	**0.034**	Linear
left lingula grey matter (mm3)	5,904 [5,479, 6,353]	5,600 [4,871, 6,313]	**0.021**	Mann-U	-0.46 (-0.77, -0.15)	**0.004**	-0.49 (-0.80, -0.17)	**0.003**	Linear
Right lingual grey matter (mm3)	6,350 [5,861, 6,782]	5,942 [5,280, 6,570]	**0.026**	Mann-U	-0.56 (-0.86, -0.25)	**<0.001**	-0.62 (-0.94, -0.30)	**<0.001**	Linear
left supracalcarine grey matter (mm3)	505.6 (±130.9)	437.1 (±134.8)	**0.002**	Mann-U	-0.57 (-0.93, -0.22)	**0.002**	-0.61 (-0.97, -0.25)	**<0.001**	Linear
right supracalcarine grey matter (mm3)	724.5 (±174.1)	650.1 (±181.8)	**0.004**	Mann-U	-0.50 (-0.87, -0.13)	**0.008**	-0.60 (-0.97, -0.23)	**0.002**	Linear
right thalamic grey matter volume (mm3)	2,733 [2,492, 2,869]	2,598 [2,274, 2,820]	**0.038**	Mann-U	-0.47 (-0.82, -0.12)	**0.009**	-0.50 (-0.86, -0.14)	**0.007**	Linear
Left caudate grey matter (mm3)	2,864 [2,604, 3,227]	2,683 [2,377, 3,105]	**0.030**	Mann-U	-0.54 (-0.91, -0.17)	**0.005**	-0.60 (-0.97, -0.22)	**0.002**	Linear
Right caudate grey matter (mm3)	3,067 [2,822, 3,437]	2,866 [2,604, 3,217]	**0.025**	Mann-U	-0.45 (-0.81, -0.09)	**0.015**	-0.49 (-0.86, -0.12)	**0.009**	Linear
Left putamen grey matter (mm3)	1,919 [1,498, 2,163]	1,588 [1,282, 1,878]	**0.002**	Mann-U	-0.71 (-1.08, -0.33)	**<0.001**	-0.73 (-1.11, -0.36)	**<0.001**	Linear
Right putamen grey matter (mm3)	2,006 [1,509, 2,224]	1,647 [1,313, 1,962]	**<0.001**	Mann-U	-0.79 (-1.16, -0.42)	**<0.001**	-0.81 (-1.18, -0.44)	**<0.001**	Linear
Left amydala grey matter (mm3)	1,838 (±227)	1,652 (±307)	**<0.001**	Mann-U	-0.70 (-1.06, -0.35)	**<0.001**	-0.69 (-1.05, -0.33)	**<0.001**	Linear
Right ventral striatal grey matter(mm3)	577.3 [494.8, 617.9]	469.0 [395.1, 549.4]	**<0.001**	Mann-U	-0.68 (-1.03, -0.33)	**<0.001**	-0.73 (-1.08, -0.38)	**<0.001**	Linear
Cerebellum: Left cerebellar lobules (i-iv) grey matter(mm3)	1,720 [1,507, 1,863]	1,562 [1,391, 1,768]	**0.002**	Mann-U	-0.76 (-1.14, -0.38)	**<0.001**	-0.77 (-1.15, -0.38)	**<0.001**	Linear
Cerebellum: Right cerebellar lobules (i-iv) grey matter(mm3)	1,957 [1,785, 2,070]	1,746 [1,546, 1,981]	**<0.001**	Mann-U	-0.78 (-1.17, -0.40)	**<0.001**	-0.81 (-1.20, -0.43)	**<0.001**	Linear
Cerebellum: Left cerebellar lobule (v) grey matter (mm3)	2,440 [2,092, 2,671]	2,161 [1,893, 2,416]	**<0.001**	Mann-U	-1.15 (-1.51, -0.78)	**<0.001**	-1.14 (-1.51, -0.77)	**<0.001**	Linear
Cerebellum: Right cerebellar lobule (v) grey matter (mm3)	2,271 (±321)	2,061 (±408)	**<0.001**	Mann-U	-1.02 (-1.39, -0.65)	**<0.001**	-1.06 (-1.43, -0.69)	**<0.001**	Linear
Cerebellum:Vermis lobule (vi) grey matter (mm3)	1,301 (±180)	1,221 (±256)	**0.007**	Mann-U	-0.67 (-1.05, -0.29)	**<0.001**	-0.70 (-1.08, -0.32)	**<0.001**	Linear
Cerebellum: Left Crus lobule (ii) grey matter(mm3)	7,408 (±1,492)	6,760 (±1,515)	**0.004**	Mann-U	-0.65 (-1.04, -0.25)	**0.002**	-0.67 (-1.07, -0.27)	**0.001**	Linear
Cerebellum: Vermis vi-ib (mm3)	107.4 (±25.3)	96.5 (±31.7)	**0.024**	Mann-U	-0.66 (-1.06, -0.27)	**0.001**	-0.66 (-1.07, -0.26)	**0.001**	Linear

Cerebellum: Vermis_vii_ia(mm3)	698.3 (±154.4)	628.8 (±166.1)	**0.014**	Mann-U	-0.81 (-1.20, -0.42)	**<0.001**	-0.84 (-1.23, -0.45)	**<0.001**	Linear
Cerebellum: Left vii -ib (mm3)	2,306 (±441)	2,145 (±444)	**0.020**	Mann-U	-0.51 (-0.89, -0.14)	**0.008**	-0.51 (-0.89, -0.12)	**0.010**	Linear
Cerebellum: Left cerebellar lobule (ix) grey matter (mm3)	1,517 (±254)	1,389 (±291)	**0.005**	Mann-U	-0.62 (-1.00, -0.24)	**0.001**	-0.66 (-1.04, -0.28)	**<0.001**	Linear
Cerbellum: Vermis ix (mm3)	320.8 (±64.8)	294.4 (±84.9)	0.053	Mann-U	-0.65 (-1.05, -0.26)	**0.001**	-0.65 (-1.05, -0.25)	**0.001**	Linear
Cerebellum:Right cerebellar lobule (ix) grey matter (mm3)	1,697 (±310)	1,560 (±355)	**0.010**	Mann-U	-0.66 (-1.05, -0.28)	**<0.001**	-0.65 (-1.03, -0.27)	**<0.001**	Linear
Cerebellum: Left cerebellar lobule (x) grey matter (mm3)	534.4 (±84.1)	496.1 (±74.1)	**0.011**	Mann-U	-0.40 (-0.78, -0.02)	**0.040**	-0.38 (-0.78, 0.01)	0.054	Linear
Amydala: Right medial nucleas amydala grey matter (mm3)	27.4 (±7.1)	22.6 (±6.1)	**<0.001**	Mann-U	-1.06 (-1.50, -0.61)	**<0.001**	-1.06 (-1.51, -0.61)	**<0.001**	Linear
Hippocampus: GC-ML-DG-head in the left hemisphere (mm3)	178.6 (±22.6)	164.5 (±24.9)	**<0.001**	Mann-U	-0.54 (-0.96, -0.12)	**0.012**	-0.52 (-0.95, -0.09)	**0.018**	Linear
Hippocampus: CA3-body in the left hemisphere (mm3)	115.6 (±18.4)	108.7 (±16.4)	**0.029**	Mann-U	-0.75 (-1.19, -0.30)	**0.001**	-0.74 (-1.19, -0.28)	**0.002**	Linear
Hippocampus: CA3-head in the left hemisphere (mm3)	162.8 (±19.3)	143.3 (±23.6)	**<0.001**	Mann-U	-0.89 (-1.30, -0.47)	**<0.001**	-0.88 (-1.30, -0.45)	**<0.001**	Linear
Hippocampus: HATA in the left hemisphere (mm3)	62.3 (±10.6)	56.8 (±11.5)	**0.008**	Mann-U	-0.57 (-1.00, -0.13)	**0.011**	-0.54 (-0.98, -0.10)	**0.016**	Linear
Hippocampus: GC-ML-DG-head in the right hemisphere (mm3)	182.5 (±29.3)	169.7 (±26.4)	**0.012**	Mann-U	-0.65 (-1.07, -0.23)	**0.003**	-0.65 (-1.08, -0.22)	**0.003**	Linear
Thalamus: CA3-head in the right hemisphere (mm3)	164.0 (±25.4)	151.9 (±24.8)	**0.002**	Mann-U	-0.63 (-1.07, -0.20)	**0.005**	-0.62 (-1.06, -0.17)	**0.007**	Linear
Thalamus: CL in the left hemisphere (mm3)	30.4 [26.6, 33.2]	28.1 [24.2, 32.2]	**0.024**	Mann-U	-0.83 (-1.26, -0.39)	**<0.001**	-0.84 (-1.28, -0.40)	**<0.001**	Linear
Thalamus: LP in the left hemisphere (mm3)	123.1 (±18.1)	113.6 (±19.5)	**0.007**	Mann-U	-0.76 (-1.18, -0.34)	**<0.001**	-0.76 (-1.19, -0.33)	**<0.001**	Linear
Thalamus: LD in the left hemisphere (mm3)	25.7 (±7.3)	21.7 (±8.0)	**0.005**	Mann-U	-1.00 (-1.46, -0.54)	**<0.001**	-1.03 (-1.49, -0.57)	**<0.001**	Linear
Desikan-Killiany parcellation									
Cuneus in the left hemisphere (mm3)	3,286 (±549)	3,138 (±574)	0.108	Mann-U	-0.55 (-0.97, -0.13)	**0.010**	-0.61 (-1.04, -0.19)	**0.005**	Linear
Posteriorcingulate in the left hemisphere (mm3)	3,503 (±518)	3,320 (±545)	**0.026**	Mann-U	-0.56 (-0.98, -0.14)	**0.009**	-0.63 (-1.05, -0.21)	**0.004**	Linear
Supramarginal in the left hemisphere (mm3)	12,614 (±1,721)	11,825 (±1,969)	**0.018**	Mann-U	-0.40 (-0.82, 0.02)	0.060	-0.47 (-0.89, -0.04)	**0.030**	Linear
Desikan-Killiany-Tourville parcellation									
Caudalanteriorcingulate in the left hemisphere (mm3)	3,366 (±553)	3,080 (±580)	**0.005**	Mann-U	-0.76 (-1.18, -0.34)	**<0.001**	-0.78 (-1.21, -0.36)	**<0.001**	Linear
Cuneus in the left hemisphere generated by ml)	4,564 (±691)	4,342 (±751)	0.069	Mann-U	-0.53 (-0.95, -0.11)	**0.013**	-0.60 (-1.03, -0.17)	**0.006**	Linear
Posteriorcingulate in the left hemisphere (mm3)	3,755 (±537)	3,581 (±562)	**0.036**	Mann-U	-0.55 (-0.96, -0.15)	**0.008**	-0.61 (-1.02, -0.20)	**0.004**	Linear
Supramarginal in the left hemisphere (mm3)	11,475 (±1,580)	10,792 (±1,807)	**0.024**	Mann-U	-0.41 (-0.83, 0.01)	0.053	-0.48 (-0.90, -0.06)	**0.026**	Linear
Destrieux (a2009s) parcellation									
G+S-cingul-Mid-Post in the left hemisphere (mm3)	2,750 (±384)	2,637 (±426)	0.087	Mann-U	-0.58 (-1.00, -0.16)	**0.007**	-0.67 (-1.09, -0.25)	**0.002**	Linear
G-cuneus in the left hemisphere (mm3)	3,125 (±526)	2,973 (±510)	0.072	Mann-U	-0.55 (-0.97, -0.12)	**0.012**	-0.59 (-1.02, -0.16)	**0.008**	Linear
G-Ins-lg+S-cent-ins in the left hemisphere (mm3)	1,609 (±229)	1,498 (±233)	**0.004**	Mann-U	-0.48 (-0.92, -0.05)	**0.030**	-0.43 (-0.88, 0.02)	0.060	Linear
G-insular-short in the left hemisphere (mm3)	2,509 (±312)	2,378 (±310)	**0.016**	Mann-U	-0.39 (-0.81, 0.03)	0.069	-0.38 (-0.81, 0.05)	0.085	Linear
G-pariet-inf-Supramar in the left hemisphere (mm3)	7,296 (±1,084)	6,747 (±1,247)	**0.006**	Mann-U	-0.51 (-0.93, -0.09)	**0.018**	-0.59 (-1.01, -0.17)	**0.007**	Linear
G-subcallosal in the left hemisphere generated (mm3)	1,384 (±384)	1,211 (±354)	**0.005**	Mann-U	-0.45 (-0.87, -0.03)	**0.038**	-0.45 (-0.88, -0.02)	**0.040**	Linear

Part B: Clinical characteristics of patients with and without brain MRI abnormalities

Variable	No brain abnormalities (n= 109)	Brain abnormalities (n= 109)	P-value	Univariate test	Odds Ratio or Beta coefficient	p-value	Test	Total N	No-abn N	Abn N
Age (years)	53·0 (±11·5)	61·5 (±10·8)	**<0.001**	T/Welch	8.50 yrs [5.53, 11.48]*			218	109	109
Female n (%)	34 (31·2%)	47 (43·1%)						218	109	109
Non-white ethnicity n (%)	36 (33·0%)	19 (17·4%)	**0.013**	X²	0·73 (0·35, 1·53)	0.405	Logistic	218	109	109
Obesity n (%)	51 (46·8%)	55 (50·5%)	0.684	X²	1·27 (0·71, 2·27)	0.426	Logistic	218	109	109
Systolic blood pressure (mm/Hg)	130·4 (±14·3)	134·8 (±16·2)	**0.034**	T/Welch	0·01 (-0·22, 0·24)	0.962	Linear	218	109	109
Pre-existing comorbidities										
Diabetes n (%)	15 (13·9%)	31 (28·4%)	**0.014**	X²	2·29 (0·95, 5·82)	0.071	Logistic	217	108	109
Hypertension n (%)	44 (41·1%)	63 (58·9%)	**0.014**	X²	1·35 (0·73, 2·50)	0.340	Logistic	214	107	107
Neurological n (%)	1 (0·9%)	5 (4·6%)	0.212	Fisher	4·14 (0·51, 90·64)	0.240	Logistic	217	108	109
Charlson index of zero or one n (%)	96 (88·1%)	88 (80·7%)	0.191	Fisher				218	109	109

Charlson index of two or more n (%)	13 (11·9%)	21 (19·3%)								
WHO clinical progression scale n (%)										
WHO – class 3-4	21 (19·6%)	15 (13·9%)	0.264	Fisher				215	107	108
WHO – class 5	53 (49·5%)	68 (63·0%)								
WHO – class 6	25 (23·4%)	19 (17·6%)								
WHO – class 7-9	8 (7·5%)	6 (5·6%)								
Admission duration (days)	6·0 [3·0, 10·0]	5·50 [3·00, 9·25]	0.3622	Mann-U	-0·20 (-0·49, 0·10)	0.1923	Norm linear	215	107	108
Acute biochemistry										
Acute CRP > 5mg/L n (%)	103 (99·0%)	95 (92·2%)	**0.019**	Fisher	0·09 (0·00, 0·59)	**0.035**	Logistic	207	104	103
Acute bilirubin	9·0 [6·0, 12·0]	10·0 [7·0, 13·8]	0.081	Mann-U	0·39 (0·11, 0·67)	**0.006**	Linear	195	97	98
Treatments										
Remdesivir n (%)	11 (22·4%)	17 (32·1%)	0.386	X²	1·61 (0·62, 4·33)	0.336	Logistic	102	49	53
Systemic (oral or IV) steroids n (%)	80 (75·5%)	80 (74·1%)	0.938	X²	0·96 (0·49, 1·91)	0.913	Logistic	214	106	108
Therapeutic dose anti-coagulation n (%)	60 (56·1%)	47 (43·5%)	0.088	X²	0·45 (0·23, 0·84)	**0.013**	Logistic	215	107	108
Non-steroidal anti-inflammatory (NSAID) n (%)	13 (12·1%)	8 (7·4%)	0.347	X²	0·42 (0·15, 1·14)	0.095	Logistic	215	107	108
Follow-up investigations										
Albumin: creatinine ratio (ACR) Result (mg/mmol)	1·10 [0·60, 1·45]	1·25 [0·90, 3·05]	**0.034**	Mann-U	0·56 (0·06, 1·05)	**0.029**	Linear	79	43	36
ACR > 10 (mg/mmol) n (%)	2 (4·7%)	4 (11·1%)	0.403	Fisher	5·52 (0·56, 107·82)	0.184	Logistic	79	43	36
Platelets - Result (10^9/L)	247·9 (±66·7)	254·3 (±66·9)	0.520	T/Welch	0·25 (-0·06, 0·57)	0.109	Linear	184	89	95
Platelets > 400x10^9/L n (%)	1 (1·1%)	5 (5·3%)	0.213	Fisher	16·69 (1·51, 505·05)	**0.046**	Logistic	184	89	95
White blood cell count (10^9/L)	6·2 [5·2, 7·7]	6·8 [6·1, 8·5]	**0.011**	Mann-U	0·44 (0·12, 0·76)	**0.008**	Linear	184	89	95
Do you feel recovered? n (%)										
No	48 (51·1%)	46 (46·0%)	0.747	Fisher				194	94	100
Not sure	21 (22·3%)	23 (23·0%)								
Yes	25 (26·6%)	31 (31·0%)								
Reported outcomes										
Headache n (%)	33 (34·7%)	40 (39·6%)	0.578	X²	1·27 (0·66, 2·49)	0.476	Logistic	196	95	101
Confusion/ fuzzy head n (%)	36 (37·9%)	31 (30·7%)	0.362	X²	0·70 (0·36, 1·38)	0.307	Logistic	196	95	101
Difficulty with communication n (%)	21 (22·1%)	17 (16·8%)	0.452	X²	0·52 (0·22, 1·20)	0.128	Logistic	196	95	101
Dizziness or light headedness n (%)	27 (28·4%)	29 (29·0%)	1.000	X²	0·91 (0·46, 1·81)	0.791	Logistic	195	95	100
Fainting / blackouts n (%)	2 (2·1%)	1 (1·0%)	0.614	Fisher				195	95	100
Short term memory loss n (%)	39 (41·1%)	45 (44·6%)	0.726	X²	1·03 (0·55, 1·94)	0.920	Logistic	196	95	101
Loss of sense of smell n (%)	13 (13·7%)	12 (11·9%)	0.870	X²	0·69 (0·26, 1·78)	0.440	Logistic	196	95	101
Loss of taste n (%)	15 (15·8%)	12 (11·9%)	0.558	X²	0·65 (0·26, 1·62)	0.362	Logistic	196	95	101
FACIT fatigue score	37·3 (±11·7)	36·1 (±11·7)	0.475	T/Welch	-0·03 (-0·33, 0·27)	0.8456	Norm linear	186	92	94
Anxiety (GAD-7) score	3·00 [0·75, 6·25]	2·00 [0·00, 7·00]	0.607	Mann-U	0·07 (-0·23, 0·38)	0.648	Norm linear	188	92	96
Anxiety (GAD-7 >8) n (%)	14 (15·2%)	17 (17·7%)	0.792	X²	2·51 (0·97, 6·87)	0.063	Logistic	188	92	96
Depression (PHQ-9) score	4·00 [2·00, 10·00]	4·00 [1·00, 11·00]	0.996	Mann-U	0·09 (-0·22, 0·39)	0.570	Norm linear	186	90	96
Depression (PHQ-9 >= 10) n (%)	24 (26·7%)	26 (27·1%)	1.000	X²	1·27 (0·59, 2·77)	0.535	Logistic	186	90	96
EQ-5D-5L utility	78·2 [70·0, 87·9]	76·8 [67·6, 100·0]	0.711	Mann-U	0·19 (-0·11, 0·49)	0.203	Norm linear	188	92	96
EQ-5D-5L less than 60% n (%)	15 (16·3%)	13 (13·5%)	0.744	X²	0·58 (0·22, 1·51)	0.266	Logistic	188	92	96
MOCA total score corrected	26·0 [25·0, 28·0]	27·0 [25·0, 28·0]	0.900	Mann-U	0·06 (-0·27, 0·39)	0.723	Norm linear	175	87	88
MOCA (corrected) <23 n (%)	7 (8·0%)	9 (10·2%)	0.794	Fisher				175	87	88

Part C: Clinical characteristics of patients with and without brain MRI abnormalities after excluding pre-existing neurological conditions

Variable	No brain abnormalities (n= 108)	Brain abnormalities (n= 104)	P-value	Univariate test	Odds Ratio or Beta coefficient	p-value	Test	Total N	No-abn N	Abn N
Age (years)	53.1 (±11.5)	61.2 (±10.8)	**<0.001**	T/Welch	8.21 yrs [5.17, 11.25]*			212	108	104
Female n (%)	34 (31.5%)	44 (42.3%)						212	108	104
Non-white ethnicity n (%)	36 (33.3%)	19 (18.3%)	**0.019**	X²	0.76 (0.36, 1.58)	0.457	Logistic	212	108	104
Obesity n (%)	51 (47.2%)	52 (50.0%)	0.789	X²	1.23 (0.69, 2.23)	0.481	Logistic	212	108	104
Systolic blood pressure (mm/Hg)	130.6 (±14.3)	134.0 (±16.0)	0.102	T/Welch	-0.02 (-0.25, 0.22)	0.890	Linear	212	108	104
Pre-existing comorbidities										
Diabetes n (%)	15 (14.0%)	30 (28.8%)	**0.014**	X²	2.66 (1.08, 6.95)	**0.038**	Logistic	211	107	104
Hypertension n (%)	44 (41.5%)	58 (56.9%)	**0.038**	X²	1.26 (0.68, 2.34)	0.462	Logistic	208	106	102
Neurological n (%)	0 (0.0%)	0 (0.0%)						211	107	104
Charlson index of zero or one n (%)	95 (88.0%)	86 (82.7%)	0.333	Fisher				212	108	104
Charlson index of two or more n (%)	13 (12.0%)	18 (17.3%)								
WHO clinical progression scale n (%)										
WHO – class 3-4	21 (19.8%)	14 (13.6%)	0.291	Fisher				209	106	103
WHO – class 5	53 (50.0%)	65 (63.1%)								
WHO – class 6	24 (22.6%)	18 (17.5%)								
WHO – class 7-9	8 (7.5%)	6 (5.8%)								
Admission duration (days)	6.0 [3.0, 10.0]	5.00 [3.00, 10.00]	0.387	Mann-U	-0.19 (-0.49, 0.10)	0.202	Linear	209	106	103
Acute biochemistry										
Acute CRP > 5mg/L n (%)	102 (99.0%)	91 (91.9%)	**0.017**	Fisher	0.09 (0.00, 0.59)	**0.034**	Logistic	202	103	99
Acute bilirubin	9.0 [6.0, 12.0]	10.0 [7.0, 13.0]	0.094	Mann-U	0.37 (0.09, 0.65)	**0.009**	Linear	190	96	94
Treatments										
Remdesivir n (%)	10 (20.8%)	17 (32.7%)	0.267	X²	1.75 (0.66, 4.80)	0.263	Logistic	100	48	52
Systemic (oral or IV) steroids n (%)	79 (75.2%)	76 (73.8%)	0.935	X²	0.96 (0.48, 1.91)	0.908	Logistic	208	105	103
Therapeutic dose anti-coagulation n (%)	59 (55.7%)	44 (42.7%)	0.083	X²	0.44 (0.23, 0.83)	**0.013**	Logistic	209	106	103
Non-steroidal anti-inflammatory (NSAID) n (%)	13 (12.3%)	8 (7.8%)	0.395	X²	0.44 (0.15, 1.18)	0.111	Logistic	209	106	103
Follow-up investigations										
Albumin: creatinine ratio (ACR) Result (mg/mmol)	1.10 [0.60, 1.45]	1.35 [0.93, 3.15]	**0.020**	Mann-U	0.59 (0.09, 1.08)	**0.022**	Linear	77	43	34
ACR > 10 (mg/mmol) n (%)	2 (4.7%)	4 (11.8%)	0.397	Fisher	5.48 (0.57, 104.84)	0.182	Logistic	77	43	34
Platelets - Result (10^9/L)	247.9 (±66.7)	255.1 (±67.7)	0.477	T/Welch	0.27 (-0.04, 0.59)	0.088	Linear	179	89	90
Platelets > 400x10^9/L n (%)	1 (1.1%)	5 (5.6%)	0.211	Fisher	18.32 (1.59, 595.14)	**0.044**	Logistic	179	89	90
White blood cell count (10^9/L)	6.20 [5.24, 7.68]	6.8 [6.1, 8.6]	**0.006**	Mann-U	0.50 (0.18, 0.83)	**0.003**	Linear	179	89	90
Do you feel recovered? n (%)										
No	48 (51.6%)	43 (45.3%)	0.713	Fisher				188	93	95
Not sure	20 (21.5%)	23 (24.2%)								
Yes	25 (26.9%)	29 (30.5%)								
Reported outcomes										
Headache n (%)	33 (35.1%)	38 (39.6%)	0.626	X²	1.25 (0.64, 2.45)	0.515	Logistic	190	94	96
Confusion/ fuzzy head n (%)	36 (38.3%)	28 (29.2%)	0.239	X²	0.65 (0.33, 1.29)	0.223	Logistic	190	94	96
Difficulty with communication n (%)	21 (22.3%)	16 (16.7%)	0.421	X²	0.50 (0.21, 1.15)	0.109	Logistic	190	94	96
Dizziness or light headedness n (%)	27 (28.7%)	26 (27.4%)	0.964	X²	0.86 (0.43, 1.73)	0.677	Logistic	189	94	95
Fainting / blackouts n (%)	2 (2.1%)	1 (1.1%)	0.621	Fisher				189	94	95
Short term memory loss n (%)	39 (41.5%)	42 (43.8%)	0.866	X²	0.98 (0.52, 1.86)	0.953	Logistic	190	94	96
Loss of sense of smell n (%)	13 (13.8%)	12 (12.5%)	0.955	X²	0.73 (0.28, 1.88)	0.514	Logistic	190	94	96
Loss of taste n (%)	15 (16.0%)	12 (12.5%)	0.635	X²	0.69 (0.27, 1.69)	0.415	Logistic	190	94	96
FACIT fatigue score	37.2 (±11.7)	36.3 (±11.6)	0.617	T/Welch	-0.02 (-0.33, 0.28)	0.879	Linear	180	91	89
Anxiety (GAD-7) score	3.00 [0.50, 6.50]	2.00 [0.00, 7.00]	0.505	Mann-U	0.05 (-0.26, 0.35)	0.772	Linear	182	91	91
Anxiety (GAD-7 >8) n (%)	14 (15.4%)	15 (16.5%)	1.000	X²	2.30 (0.87, 6.37)	0.098	Logistic	182	91	91

Depression (PHQ-9) score	4.00 [2.00, 10.00]	4.00 [1.00, 10.50]	0.960	Mann-U	0.07 (-0.24, 0.38)	0.640	Linear	180	89	91
Depression (PHQ-9 >= 10) n (%)	24 (27.0%)	24 (26.4%)	1.000	X^2	1.23 (0.57, 2.67)	0.604	Logistic	180	89	91
EQ-5D-5L utility	76.8 [69.7, 87.9]	76.8 [67.2, 100.0]	0.648	Mann-U	0.21 (-0.09, 0.51)	0.170	Linear	182	91	91
EQ-5D-5L less than 60% n (%)	15 (16.5%)	12 (13.2%)	0.677	X^2	0.54 (0.20, 1.42)	0.214	Logistic	182	91	91
MOCA total score corrected	26.0 [25.0, 28.0]	27.0 [25.0, 28.0]	0.817	Mann-U	0.09 (-0.24, 0.43)	0.580	Linear	169	86	83
MOCA (corrected) <23 n (%)	7 (8.1%)	8 (9.6%)	0.792	Fisher						

Abn N number of cases with abnormal brain MRI, BMI body mass index, BNP B-type natriuretic peptide, C-reactive protein, EQ-5D-5L quality of life, FACIT Functional Assessment of Chronic Illness Therapy, GFR glomerular filtration rate, GAD-7 generalised anxiety disorder, IV intravenous, KCO carbon monoxide transfer co-efficient, FEV1 forced expiratory volume in 1 second, FVC forced vital capacity, No-Abn N number of cases without abnormal brain MRI, NT N-terminal, cT1n I troponin I, MOCA montreal cognitive assessment, PHQ-9 personal health questionnaire, SQR square, TLCO transfer capacity of lung, ULN upper limit of normal, WHO World health organisation. For Part A, inverse probability weighting was used to adjust imaging variable for confounders which included age, sex, body mass index, smoking, hypertension, hypercholesterolemia, diabetes, cardiac, brain, liver, lung and renal comorbidities, and scanner manufacturer, head size, date of imaging and brain and table position within the scanner. Part B and C (excludes corresponding organ comorbidities) regression models were adjusted for age, sex, smoking, hypertension, diabetes, Charlson comorbidity index, obesity and scanner manufacturer. *Confidence intervals for age difference are not adjusted.

Supplementary Table 5. A) Comparison of liver MRI in patients vs controls; B) Clinical characteristics of patients with and without liver MRI abnormalities; C) Clinical characteristics of patients with and without liver MRI abnormalities after excluding pre-existing liver conditions.

Part A: Comparison of liver health between patients and controls

					Multivariate analysis - adjusted inverse probability weighting (IPW)		Multivariate analysis - adjusted with IPW and excluding patients with WHO >= 7		
**Variable**	**Controls (n= 52)**	**Patients (n= 259)**	**P-value**	**Univariate test**	**Odds Ratio or Beta coefficient**	**p-value**	**Odds Ratio or Beta coefficient**	**p-value**	**Test**
Liver disease	0 (0.0%)	14 (5.4%)	0.138	Fisher		0.992		0.992	Logistic
Abnormal acute liver function n (%)		141 (57.8%)							
Abnormal follow-up liver function n (%)	16 (31.4%)	30 (13.6%)	**0.004**	X^2	0.33 (0.15, 0.70)	**0.004**	0.29 (0.14, 0.65)	**0.002**	Logistic
**Liver MRI abnormalities**									
**Liver abnormality**	28 (58.3%)	142 (60.2%)	0.940	X^2	0.69 (0.34, 1.38)	0.306	0.71 (0.34, 1.42)	0.337	Logistic
Liver iron-corrected T1	733.0 [680.8, 786.2]	771.0 [721.2, 839.8]	**0.011**	Mann-U	0.12 (-0.21, 0.45)	0.471	0.13 (-0.20, 0.46)	0.430	Linear
Liver iron concentration (mg/g)	0.82 [0.66, 1.09]	0.55 [0.48, 0.67]	**<0.001**	Mann-U	-1.22 (-1.52, -0.91)	**<0.001**	-1.22 (-1.53, -0.92)	**<0.001**	Linear
Liver proton density fat fraction	5.95 [3.58, 9.57]	5.20 [2.90, 11.70]	0.838	Mann-U	-0.22 (-0.56, 0.11)	0.191	-0.19 (-0.52, 0.15)	0.269	Linear

Part B: Clinical characteristics of patients with and without liver MRI abnormalities

**Variable**	**No liver abnormalities (n= 86)**	**Liver abnormalities (n= 137)**	**P-value**	**Univariate test**	**Odds Ratio or Beta coefficient**	**p-value**	**Test**	**Total N**	**No-abn N**	**Abn N**
Age (years)	56·8 (±13·2)	56·5 (±11·6)	0.826	T/Welch	-0.37 yrs [-3.58, 2.84]*			236	94	142
Female n (%)	36 (38·3%)	55 (38·7%)						236	94	142
Non-white thnicity n (%)	31 (33·0%)	36 (25·4%)	0.261	X^2	1·04 (0·54, 2·02)	0.912	Logistic	236	94	142
Pre-existing comorbidities n (%)										
Obesity n (%)	27 (28·7%)	92 (64·8%)	**<0.001**	X^2	5·05 (2·84, 9·23)	**<0.001**	Logistic	236	94	142
Diabetes n (%)	11 (11·8%)	34 (24·1%)	**0.031**	X^2	5·82 (2·20, 17·55)	**<0.001**	Logistic	234	93	141
Hypercholestrolemia n (%)	17 (18·3%)	23 (16·3%)	0.831	X^2	1·65 (0·70, 4·08)	0.263	Logistic	234	93	141
Hypertension n (%)	45 (48·9%)	69 (49·6%)	1.000	X^2	1·11 (0·60, 2·06)	0.743	Logistic	231	92	139
Liver disease n (%)	8 (8·6%)	5 (3·5%)	0.174	X^2	0·27 (0·06, 1·14)	0.083	Logistic	234	93	141
Charlson comorbidity index n (%)	0·00 [0·00, 1·00]	0·00 [0·00, 1·00]	0.911	Mann-U	0·49 (0·07, 0·90)	**0.022**	Linear	234	93	141
Charlson index of zero or one n (%)	71 (75·5%)	126 (88·7%)	**0.012**	Fisher				236	94	142
Charlson index of two or more n (%)	23 (24·5%)	16 (11·3%)								
WHO clinical progression scale n (%)										
WHO – class 3-4	20 (21·5%)	23 (16·5%)	0.3134	Fisher				232	93	139
WHO – class 5	51 (54·8%)	76 (54·7%)								
WHO – class 6	13 (14·0%)	31 (22·3%)								
WHO – class 7-9	9 (9·7%)	9 (6·5%)								
Acute liver injury n (%)	48 (54·5%)	78 (58·6%)	0.6426	X^2	1·05 (0·57, 1·91)	0.8721	Logistic	221	88	133
Acute CRP > 5mg/L	80 (88·9%)	128 (97·0%)	**0.0315**	X^2	6·40 (1·59, 32·28)	**0.014**	Logistic	222	90	132
Admission duration (days)	6·0 [2·0, 10·0]	6·0 [3·0, 10·0]	0.587	Mann-U	0·09 (-0·19, 0·38)	0.5164	Norm linear	232	93	139
Treatments										
Remdesivir n (%)	8 (21·6%)	14 (24·6%)	0.937	X^2	1·15 (0·37, 3·62)	0.813	Logistic	94	37	57
Systemic (oral or IV) steroids n (%)	61 (65·6%)	111 (80·4%)	**0.017**	X^2	1·99 (1·03, 3·88)	**0.041**	Logistic	231	93	138
Therapeutic dose anti-coagulation n (%)	47 (50·5%)	82 (59·0%)	0.256	X^2	1·13 (0·62, 2·04)	0.697	Logistic	232	93	139
NSAID n (%)	12 (12·9%)	10 (7·2%)	0.220	X^2	0·53 (0·19, 1·40)	0.198	Logistic	232	93	139
Follow-up lung function										
Forced expiratory volume in 1 second (L)	2·93 (±0·85)	2·76 (±0·79)	0.222	T/Welch	-0·38 (-0·66, -0·11)	**0.007**	Linear	154	63	91
FEV1 < 80% of predicted n (%)	7 (16·3%)	20 (27·4%)	0.254	X^2	2·89 (0·99, 9·74)	0.065	Logistic	116	43	73
Forced vital capacity (L)	3·79 (±1·01)	3·49 (±0·94)	0.059	T/Welch	-0·49 (-0·76, -0·21)	**<0.001**	Linear	154	63	91
FVC < 80% of predicted n (%)	5 (11·6%)	21 (28·8%)	0.057	X^2	4·31 (1·42, 15·73)	**0.016**	Logistic	116	43	73

Ratio of FEV1 and FVC	0·77 (±0·08)	0·79 (±0·10)	0.085	T/Welch	0·32 (0·00, 0·63)	**0.048**	Linear	154	63	91
Follow-up biochemistry/blood										
Abnormal follow-up liver function	6 (7·7%)	22 (18·0%)	0.065	X²	2·89 (1·07, 9·07)	**0.048**	Logistic	200	78	122
Follow-up BNP (ng/L)	69·0 [42·0, 142·0]	11·0 [10·0, 19·5]	**0.029**	Mann-U	-1·76 (-2·92, -0·61)	**0.009**	Linear	16	9	7
Follow-up ALT (U/L)	20·0 [17·0, 29·0]	27·0 [21·0, 40·0]	**<0.001**	Mann-U	0·59 (0·30, 0·88)	**<0.001**	Linear	199	77	122
Follow-up ALP (U/L)	70·0 [60·5, 82·0]	78·0 [67·8, 89·2]	**0.013**	Mann-U	0·30 (-0·01, 0·61)	0.057	Linear	195	75	120
Haemoglobin - Result (g/L)	139·0 [132·2, 148·8]	147·0 [137·0, 156·0]	**0.004**	Mann-U	0·31 (0·06, 0·56)	**0.014**	Linear	201	78	123
HbA1C - Result (%)	5·50 [5·19, 5·70]	5·80 [5·50, 6·45]	**<0.001**	Mann-U	0·87 (0·59, 1·14)	**<0.001**	Linear	180	71	109
HbA1C above 7% n(%)	3 (4·2%)	19 (17·4%)	**0.016**	X²	6·50 (1·77, 34·23)	**0.011**	Logistic	180	71	109
White blood cell count (10^9/L)	6·10 [5·25, 6·77]	7·2 [6·1, 8·8]	**<0.001**	Mann-U	0·65 (0·35, 0·94)	**<0.001**	Linear	201	78	123
Do you feel recovered?										
No	48 (56·5%)	66 (52·4%)	0.845	Fisher				211	85	126
Not sure	14 (16·5%)	24 (19·0%)								
Yes	23 (27·1%)	36 (28·6%)								
Reported outcomes										
Breathlessness n (%))	38 (44·2%)	71 (57·7%)	0.074	X²	1.58 (0.87, 2.90)	0.136	Logistic	209	86	123
Fatigue n (%)	48 (56·5%)	82 (66·7%)	0.178	X²	1·53 (0·81, 2·89)	0.187	Logistic	208	85	123
Confusion/ fuzzy head n (%)	32 (36·8%)	50 (39·7%)	0.776	X²	0·93 (0·49, 1·76)	0.821	Logistic	213	87	126
Dizziness or light headedness n (%)	25 (29·1%)	43 (34·1%)	0.532	X²	1·24 (0·66, 2·38)	0.507	Logistic	212	86	126
Joint pain n (%)	40 (46·0%)	57 (45·6%)	1.000	X²	0·88 (0·48, 1·61)	0.674	Logistic	212	87	125
Abdominal pain n (%)	22 (25·3%)	26 (20·6%)	0.527	X²	0·66 (0·32, 1·38)	0.272	Logistic	213	87	126
Nausea/vomiting n (%)	8 (9·2%)	9 (7·1%)	0.775	X²	0·80 (0·27, 2·38)	0.680	Logistic	213	87	126
Diarrhea n (%)	7 (8·0%)	16 (12·8%)	0.384	X²	1·21 (0·44, 3·60)	0.718	Logistic	212	87	125
FACIT fatiugue Score	36·1 (±11·5)	35·2 (±11·7)	0.594	T/Welch	0·07 (-0·22, 0·35)	0.654	Norm linear	201	83	118
Anxiety (GAD-7 score)	3·00 [1·00, 8·00]	3·50 [0·00, 8·00]	0.855	Mann-U	-0·02 (-0·30, 0·25)	0.867	Norm linear	203	83	120
Anxiety (GAD-7 >8) n (%)	16 (19·3%)	27 (22·5%)	0.706	X²	1·06 (0·48, 2·39)	0.884	Logistic	203	83	120
Depression (PHQ-9) score	5·99 (±5·64)	7·46 (±6·71)	0.095	T/Welch	0·14 (-0·15, 0·42)	0.345	Norm linear	201	83	118
Depression (PHQ-9 >= 10) n (%)	17 (20·5%)	43 (36·4%)	**0.023**	X²	2·10 (1·01, 4·51)	**0.050**	Logistic	201	83	118
EQ-5D-5L utility	76·7 [61·2, 87·9]	76·8 [67·3, 90·6]	0.292	Mann-U	0·28 (-0·01, 0·57)	0.061	Linear	203	81	122
EQ-5D-5L less than 60% n (%)	19 (23·5%)	16 (13·1%)	0.085	X²	0·28 (0·12, 0·66)	**0.004**	Logistic	203	81	122

Part C: Clinical characteristics of patients with and without liver MRI abnormalities after excluding pre-existing liver conditions

Variable	No liver abnormalities (n= 86)	Liver abnormalities (n= 137)	P-value	Univariate test	Odds Ratio or Beta coefficient	p-value	Test	Total N	No-abn N	Abn N
Age (years)	56.3 (±13.4)	56.6 (±11.7)	0.870	T/Welch	0.22 yrs [-3.16, 3.60]*			223	86	137
Female n (%)	31 (36.0%)	51 (37.2%)						223	86	137
Non-white thnicity n (%)	28 (32.6%)	34 (24.8%)	0.270	X²	1.05 (0.54, 2.09)	0.877	Logistic	223	86	137
Pre-existing comorbidities n (%)										
Obesity n (%)	25 (29.1%)	87 (63.5%)	**<0.001**	X²	4.62 (2.56, 8.56)	**<0.001**	Logistic	223	86	137
Diabetes n (%)	10 (11.8%)	33 (24.3%)	**0.033**	X²	9.42 (3.01, 36.03)	**<0.001**	Logistic	221	85	136
Hypercholestrolemia n (%)	15 (17.6%)	22 (16.2%)	0.921	X²	1.63 (0.67, 4.16)	0.290	Logistic	221	85	136
Hypertension n (%)	43 (51.2%)	67 (50.0%)	0.975	X²	0.95 (0.50, 1.80)	0.865	Logistic	218	84	134
Liver disease n (%)	0 (0.0%)	0 (0.0%)						221	85	136
Charlson comorbidity index n (%)	0.00 [0.00, 1.00]	0.00 [0.00, 1.00]	0.730	Mann-U				221	85	136
Charlson index of zero or one n (%)	68 (79.1%)	124 (90.5%)	**0.027**	Fisher				223	86	137
Charlson index of two or more n (%)	18 (20.9%)	13 (9.5%)								
WHO clinical progression scale n (%)										
WHO – class 3-4	16 (18.8%)	21 (15.7%)	0.363	Fisher				219	85	134
WHO – class 5	48 (56.5%)	74 (55.2%)								
WHO – class 6	12 (14.1%)	30 (22.4%)								
WHO – class 7-9	9 (10.6%)	9 (6.7%)								
Acute liver injury n (%)	45 (56.2%)	76 (59.4%)	0.764	X²	0.99 (0.53, 1.84)	0.966	Logistic	208	80	128
Acute CRP > 5mg/L	75 (91.5%)	123 (96.9%)	0.115	Fisher	4.11 (0.90, 22.45)	0.079	Logistic	209	82	127
Admission duration (days)	6.0 [3.0, 10.0]	6.0 [3.0, 10.0]	0.677	Mann-U	0.06 (-0.24, 0.35)	0.709	Linear	219	85	134
Treatments										
Remdesivir n (%)	7 (21.2%)	13 (23.6%)	1.000	X²	1.21 (0.38, 4.05)	0.747	Logistic	88	33	55

Systemic (oral or IV) steroids n (%)	58 (68.2%)	108 (81.2%)	**0.043**	X^2	1.74 (0.88, 3.48)	0.113	Logistic	218	85	133
Therapeutic dose anti-coagulation n (%)	43 (50.6%)	78 (58.2%)	0.334	X^2	1.10 (0.59, 2.03)	0.766	Logistic	219	85	134
NSAID n (%)	10 (11.8%)	8 (6.0%)	0.204	X^2	0.51 (0.17, 1.47)	0.216	Logistic	219	85	134
Follow-up lung function										
Forced expiratory volume in 1 second (L)	2.98 (±0.86)	2.77 (±0.80)	0.152	T/Welch	-0.39 (-0.68, -0.11)	**0.008**	Linear	145	56	89
FEV1 < 80% of predicted n (%)	7 (17.9%)	20 (27.8%)	0.357	X^2	2.78 (0.93, 9.72)	0.082	Logistic	111	39	72
Forced vital capacity (L)	3.86 (±1.00)	3.50 (±0.95)	**0.035**	T/Welch	-0.51 (-0.80, -0.22)	**<0.001**	Linear	145	56	89
FVC < 80% of predicted n (%)	4 (10.3%)	21 (29.2%)	**0.042**	X^2	5.41 (1.62, 23.34)	**0.011**	Logistic	111	39	72
Ratio of FEV1 and FVC	0.77 (±0.08)	0.79 (±0.10)	0.094	T/Welch	0.34 (0.01, 0.66)	**0.041**	Linear	145	56	89
Follow-up biochemistry/blood										
Abnormal follow-up liver function	4 (5.7%)	21 (17.6%)	**0.034**	X^2	3.60 (1.20, 13.74)	**0.035**	Logistic	189	70	119
Follow-up BNP (ng/L)	97.6 (±68.5)	21.6 (±22.5)	**0.017**	T/Welch	-1.77 (-3.06, -0.48)	**0.015**	Linear	15	8	7
Follow-up ALT (U/L)	20.0 [17.0, 29.0]	27.0 [21.0, 40.0]	**<0.001**	Mann-U	0.58 (0.29, 0.87)	**<0.001**	Linear	188	69	119
Follow-up ALP (U/L)	72.8 (±19.4)	79.1 (±21.0)	**0.041**	T/Welch	0.31 (-0.01, 0.62)	0.056	Linear	186	68	118
Haemoglobin - Result (g/L)	140.0 [133.0, 149.8]	147.0 [138.0, 156.2]	**0.006**	Mann-U	0.33 (0.07, 0.59)	**0.014**	Linear	190	70	120
HbA1C - Result (%)	5.50 [5.19, 5.70]	5.80 [5.50, 6.45]	**<0.001**	Mann-U	0.86 (0.58, 1.14)	**<0.001**	Linear	170	63	107
HbA1C above 7% n(%)	3 (4.8%)	18 (16.8%)	**0.039**	X^2	6.50 (1.65, 38.52)	**0.017**	Logistic	170	63	107
White blood cell count (10^9/L)	6.15 [5.25, 7.02]	7.2 [6.1, 8.7]	**<0.001**	Mann-U	0.62 (0.31, 0.93)	**<0.001**	Linear	190	70	120
Do you feel recovered? n (%)										
No	44 (57.1%)	64 (52.5%)	0.768	Fisher				199	77	122
Not sure	12 (15.6%)	23 (18.9%)								
Yes	21 (27.3%)	35 (28.7%)								
Reported outcomes										
Breathlessness n (%)	35 (44.9%)	69 (58.0%)	0.098	X^2	1.54 (0.83, 2.87)	0.170	Logistic	197	78	119
Fatigue n (%)	42 (54.5%)	78 (65.5%)	0.163	X^2	1.55 (0.82, 2.95)	0.182	Logistic	196	77	119
Confusion/ fuzzy head n (%)	29 (36.7%)	49 (40.2%)	0.732	X^2	0.97 (0.50, 1.87)	0.918	Logistic	201	79	122
Dizziness or light headedness n (%)	21 (26.9%)	41 (33.6%)	0.401	X^2	1.32 (0.68, 2.63)	0.418	Logistic	200	78	122
Joint pain n (%)	34 (43.0%)	55 (45.5%)	0.849	X^2	1.01 (0.54, 1.90)	0.966	Logistic	200	79	121
Abdominal pain n (%)	18 (22.8%)	25 (20.5%)	0.833	X^2	0.72 (0.33, 1.57)	0.411	Logistic	201	79	122
Nausea/vomiting n (%)	7 (8.9%)	8 (6.6%)	0.740	X^2	0.79 (0.25, 2.54)	0.687	Logistic	201	79	122
Diarrhea n (%)	5 (6.3%)	15 (12.4%)	0.247	X^2	1.46 (0.48, 5.06)	0.522	Logistic	200	79	121
FACIT fatigue score	36.1 (±11.7)	35.5 (±11.6)	0.744	T/Welch	0.08 (-0.21, 0.38)	0.578	Linear	189	75	114
Anxiety (GAD- 7) Score	2.00 [1.00, 8.00]	3.00 [0.00, 8.00]	0.842	Mann-U	-0.04 (-0.33, 0.26)	0.814	Linear	191	75	116
Anxiety (GAD-7 >8) n (%)	15 (20.0%)	25 (21.6%)	0.940	X^2	0.97 (0.43, 2.26)	0.948	Logistic	191	75	116
Depression (PHQ-9) score	5.99 (±5.83)	7.24 (±6.59)	0.173	T/Welch	0.12 (-0.18, 0.42)	0.431	Linear	189	75	114
Depression (PHQ-9 >= 10) n (%)	16 (21.3%)	40 (35.1%)	0.062	X^2	1.88 (0.89, 4.12)	0.105	Logistic	189	75	114
EQ-5D-5L utility	76.7 [61.2, 88.3]	77.3 [67.9, 97.7]	0.254	Mann-U	0.29 (-0.01, 0.59)	0.054	Linear	191	73	118
EQ-5D-5L less than 60% n (%)	17 (23.3%)	14 (11.9%)	0.060	X^2	0.28 (0.11, 0.68)	**0.006**	Logistic	191	73	118

Abn N number of cases with abnormal liver MRI,, ALP Alkaline phosphatase, Aspartate aminotransaminase AST, BMI body mass index, BNP B-type natriuretic peptide, C-reactive protein, EQ-5D-5L quality of life, GFR glomerular filtration rate, GAD-7 generalised anxiety disorder, IV intravenous, FACIT Functional Assessment of Chronic Illness Therapy, FEV1 forced expiratory volume in 1 second, FVC forced vital capacity, MOCA montreal cognitive assessment, No-Abn N number of cases without abnormal liver MRI, NSAID Non steroidal anti-inflammatory, PHQ-9 personal health questionnaire, SQR square, TLCO transfer capacity of lung, ULN upper limit of normal, WHO world health organisation. For Part A, inverse probability weighting was used to adjust imaging variable for confounders which included age, sex, body mass index, smoking, hypertension, hypercholesterolemia, diabetes, cardiac, brain, liver, lung and renal comorbidities, and scanner manufacturer. Part B and Part C regression models were adjusted for following covariates: age, sex, smoking, hypertension, diabetes, Charlson comorbidity index, obesity and scanner manufacturer. *Confidence intervals for age difference are not adjusted.

Supplementary Table 6. A) Comparison of renal MRI in patients vs controls; B) Clinical characteristics of patients with and without renal abnormalities c) Clinical characteristics of patients with and without renal abnormalities after excluding pre-existing renal conditions

Part A: Comparison of renal health between patients vs controls

					Multivariate analysis - adjusted inverse probability weighting (IPW)		Multivariate analysis - adjusted with IPW and excluding patients with WHO >= 7		
**Variable**	**Controls (n= 52)**	**Patients (n= 259)**	**P-value**	**Univariate test**	**Odds Ratio or Beta coefficient**	**p-value**	**Odds Ratio or Beta coefficient**	**p-value**	**Test**
Kidney disease	1 (1.9%)	14 (5.4%)	0.480	Fisher	2.08 (0.44, 25.06)	0.441	1.87 (0.38, 22.81)	0.514	Logistic
Acute kidney injury		42 (16.5%)							
Follow-up eGFR (ml/min per 1.73m2)	90.0 [85.0, 90.0]	90.0 [76.0, 90.0]	**0.016**	Mann-U	-0.37 (-0.70, -0.04)	**0.029**	-0.39 (-0.71, -0.07)	**0.018**	Linear
Follow-up eGFR < 60	0 (0.0%)	13 (5.9%)	0.136	Fisher		0.992		0.992	Logistic
**Renal MRI metrics**									
**Kidney abnormalities**	3 (6.2%)	57 (23.2%)	**0.014**	X²	2.36 (0.89, 8.02)	0.117	2.36 (0.88, 8.03)	0.119	Logistic
Kidney MRI									
Left and right kidney volumes (ml)	312.1 (±50.4)	343.3 (±85.3)	0.117	Mann-U	0.26 (-0.26, 0.78)	0.331	0.24 (-0.28, 0.76)	0.369	Linear
Left kidney volume (ml)	153.8 (±25.9)	170.0 (±45.0)	0.106	Mann-U	0.57 (0.05, 1.09)	**0.033**	0.56 (0.04, 1.07)	**0.036**	Linear
Left and right kidney volume index (ml/m2)	164 [156, 175]	167 [145, 186]	0.690	Mann-U	0.10 (-0.43, 0.63)	0.702	0.06 (-0.46, 0.59)	0.813	Linear
Left average cortical T1 (ms)	1,545 (±67)	1,525 (±77)	0.064	Mann-U	-0.13 (-0.45, 0.19)	0.432	-0.14 (-0.46, 0.19)	0.408	Linear
left average cortical T1 standard deviation	39.8 (±8.3)	42.7 (±9.1)	**0.022**	Mann-U	0.34 (0.02, 0.67)	**0.040**	0.33 (-0.00, 0.66)	0.051	Linear
Right average cortical T1 (ms)	1,534 (±63)	1,525 (±76)	0.245	Mann-U	-0.04 (-0.37, 0.28)	0.787	-0.05 (-0.38, 0.28)	0.757	Linear
Right cortical T1 standard deviation (ms)	38.7 (±9.1)	41.0 (±8.6)	**0.049**	Mann-U	0.17 (-0.16, 0.50)	0.304	0.15 (-0.18, 0.47)	0.375	Linear
Left average medullary T1 (ms)	1,934 (±75)	1,895 (±90)	**0.005**	Mann-U	-0.26 (-0.58, 0.05)	0.105	-0.27 (-0.59, 0.04)	0.091	Linear
Left medullary cortical T1 standard deviation	62.9 (±13.1)	56.0 (±13.4)	**0.003**	Mann-U	-0.12 (-0.43, 0.19)	0.448	-0.09 (-0.40, 0.22)	0.565	Linear
Right average medullary T1 (ms)	1,938 (±73)	1,896 (±89)	**0.001**	Mann-U	-0.34 (-0.66, -0.02)	**0.037**	-0.34 (-0.66, -0.02)	**0.038**	Linear
Right medullary T1 standard deviation (ms)	60.9 (±12.5)	55.4 (±13.4)	**0.011**	Mann-U	-0.18 (-0.51, 0.15)	0.276	-0.14 (-0.47, 0.18)	0.384	Linear
Left corticomedullary differentiation (ms)	399.6 (±51.0)	370.3 (±59.8)	**0.002**	Mann-U	-0.28 (-0.61, 0.05)	0.093	-0.30 (-0.63, 0.03)	0.079	Linear
Right corticomedullary differentiation (ms)	403.6 (±55.2)	371.3 (±61.1)	**0.002**	Mann-U	-0.34 (-0.68, -0.01)	**0.041**	-0.35 (-0.68, -0.01)	**0.042**	Linear
Average cortical T1 (ms)	1,539 (±64)	1,525 (±75)	0.136	Mann-U	-0.08 (-0.40, 0.25)	0.641	-0.09 (-0.41, 0.24)	0.605	Linear
Average medullary T1 (ms)	1,936 (±72)	1,895 (±88)	**0.002**	Mann-U	-0.30 (-0.61, 0.02)	0.066	-0.30 (-0.62, 0.01)	0.062	Linear
Average corticomedullary differentiation (ms)	401.6 (±50.2)	370.5 (±58.5)	**0.001**	Mann-U	-0.32 (-0.66, 0.01)	0.055	-0.33 (-0.66, 0.00)	0.053	Linear

Part B: Clinical characteristics of patients with and without renal MRI abnormalities

**Variable**	**No renal abnormalities (n= 189)**	**Renal abnormalities (n= 57)**	**P-value**	**Univariate test**	**Odds Ratio or Beta coefficient**	**p-value**	**Test**	**Total N**	**No-Abn N**	**Abn N**
Age (years)	55·5 (±11·6)	61·3 (±13·4)	**0.004**	T/Welch	5.8 yrs (2.2, 9.4)*	<0.001	Linear	246	189	57
Female n (%)	68 (36·0%)	27 (47·4%)						246	189	57
Non-white ethnicity n (%)	48 (25·4%)	16 (28·1%)	0.817	X²	1·54 (0·71, 3·28)	0.270	Logistic	246	189	57
Body mass index (kg/m2)	30·9 (±5·4)	29·4 (±7·1)	0.139	T/Welch	-0·36 (-0·66, -0·06)	**0.018**	Linear	246	189	57
Obesity n (%)	101 (53·4%)	22 (38·6%)	0.070	X²	0·54 (0·29, 1·02)	0.059	Logistic	246	189	57
Systolic blood pressure (mm Hg)	131·7 (±14·5)	136·7 (±16·4)	**0.042**	T/Welch	0·14 (-0·10, 0·39)	0.252	Linear	246	189	57
Kidney disease n (%)	5 (2·7%)	8 (14·0%)	**0.003**	Fisher	5·92 (1·34, 32·19)	**0.024**	Logistic	244	187	57
Cardiac n (%)	21 (11·2%)	17 (29·8%)	**0.002**	X²	2·80 (1·20, 6·53)	**0.016**	Logistic	244	187	57
Hypertension n (%)	84 (45·4%)	37 (66·1%)	**0.011**	X²	1·93 (0·98, 3·86)	0.060	Logistic	241	185	56
Charlson index of zero or one n (%)	163 (86·2%)	43 (75·4%)	0.065	Fisher				246	189	57
Charlson index of two or more n (%)	26 (13·8%)	14 (24·6%)								
WHO clinical progression scale n (%)										
WHO – class 3-4	33 (17·7%)	10 (17·9%)	0.994	Fisher				242	186	56
WHO – class 5	103 (55·4%)	32 (57·1%)								

WHO – class 6	37 (19·9%)	10 (17·9%)								
WHO – class 7-9	13 (7·0%)	4 (7·1%)								
Admission duration (days)	5·50 [3·00, 10·00]	6·0 [4·0, 10·0]	0.481	Mann-U	0·10 (-0·21, 0·42)	0.513	Norm linear	242	186	56
Acute biochemistry*										
Acute cTnI Troponin	6·0 [2·0, 10·0]	179·0 [179·0, 179·0]			3·25 (0·60, 5·90)	**0.022**	Linear	18	17	1
Acute kidney injury n (%)	25 (13·4%)	16 (28·6%)	**0.015**	X²	1·97 (0·83, 4·60)	0.117	Logistic	242	186	56
Acute eGFR(ml/min per 1.73m2)	85·0 [69·8, 90·0]	75·0 [55·8, 90·0]	**0.012**	Mann-U	-0·22 (-0·50, 0·06)	0.127	Linear	238	184	54
Acute eGFR < 60ml/min per 1.73m2 n (%)	24 (13·0%)	16 (29·6%)	**0.008**	X²	2·16 (0·89, 5·14)	0.082	Logistic	238	184	54
Treatments										
Remdesivir n (%)	21 (23·6%)	8 (33·3%)	0.480	X²	1·60 (0·52, 4·71)	0.397	Logistic	113	89	24
Systemic (oral or IV) steroids n (%)	143 (77·3%)	37 (66·1%)	0.129	X²	0·57 (0·28, 1·15)	0.113	Logistic	241	185	56
Therapeutic dose anti-coagulation n (%)	95 (51·1%)	25 (44·6%)	0.489	X²	0·66 (0·33, 1·28)	0.217	Logistic	242	186	56
Non-steroidal anti-inflammatory (NSAID) n (%)	14 (7·5%)	10 (17·9%)	**0.044**	X²	2·66 (1·01, 6·81)	**0.042**	Logistic	242	186	56
Follow-up investigations										
Ratio of FEV1 and FVC (L)	0·79 (±0·09)	0·75 (±0·10)	**0.008**	T/Welch	-0·35 (-0·70, -0·00)	**0.049**	Linear	165	124	41
Follow-up eGFR (ml/min per 1.73m2)	90·0 [79·0, 90·0]	82·5 [65·2, 90·0]	**0.018**	Mann-U	-0·25 (-0·55, 0·06)	0.112	Linear	209	161	48
Follow-up eGFR < 60 (ml/min per 1.73m2) n (%)	6 (3·7%)	7 (14·6%)	**0.013**	Fisher	3·00 (0·69, 13·41)	0.139	Logistic	209	161	48
Albumin: creatinine ratio (ACR, mg/mmol)	1·20 [0·70, 2·12]	1·9 [0·9, 6·1]	0.067	Mann-U	0·37 (-0·13, 0·87)	0.142	Linear	100	80	20
ACR>10 mg/mmol (%)	3 (3·8%)	5 (25·0%)	**0.008**	Fisher	8·78 (1·59, 64·80)	**0.017**	Logistic	100	80	20
Serum creatinine (µmol/L)	76·0 [65·0, 86·2]	72·0 [63·0, 89·2]	0.435	Mann-U	-0·07 (-0·37, 0·22)	0.627	Linear	212	164	48
Serum creatinine (>104 for men, >84 for women) n (%)	13 (7·9%)	12 (25·0%)	**0.003**	X²	3·31 (1·25, 8·76)	**0.015**	Logistic	212	164	48
Follow-up Pro-NT BNP pg/ml	42·5 [35·0, 76·8]	76·0 [37·5, 152·0]	**0.004**	Mann-U	0·44 (0·08, 0·81)	**0.018**	Linear	157	122	35
Follow-up any BNP > normal limit n (%)	18 (13·8%)	14 (36·8%)	**0.003**	X²	3·08 (1·16, 8·20)	**0.023**	Logistic	168	130	38
Haemoglobin - Result (g/L)	146·0 [136·5, 156·0]	138·5 [130·8, 145·2]	**<0.001**	Mann-U	-0·27 (-0·56, 0·02)	0.067	Linear	211	163	48
Platelets - Result (10^9/L)	252·3 (±65·0)	259·5 (±92·9)	0.622	T/Welch	0·06 (-0·27, 0·39)	0.721	Linear	211	163	48
Platelets > 400 10^9/L n (%)	5 (3·1%)	4 (8·3%)	0.122	Fisher	4·85 (1·00, 24·06)	**0.045**	Logistic	211	163	48
White blood cell count (10^9/L)	6·60 [5·55, 7·90]	7·24 [6·10, 8·43]	0.101	Mann-U	0·23 (-0·12, 0·58)	0.192	Linear	211	163	48
WBC count <4 (10^9/L) n (%)	3 (1·8%)	3 (6·2%)	0.133	Fisher	6·81 (1·04, 49·92)	**0.043**	Logistic	211	163	48
Do you feel recovered? n (%)										
No	87 (50·6%)	27 (55·1%)	0.667	Fisher				221	172	49
Not sure	35 (20·3%)	11 (22·4%)								
Yes	50 (29·1%)	11 (22·4%)								
Reported outcomes										
Abdominal pain n (%)	33 (19·1%)	15 (30·0%)	0.144	X²	1·87 (0·84, 4·10)	0.118	Logistic	223	173	50
Nausea/vomiting n (%)	13 (7·5%)	2 (4·0%)	0.530	Fisher	0·55 (0·08, 2·30)	0.469	Logistic	223	173	50
Loss of control of passing urine n (%)	15 (8·7%)	6 (12·0%)	0.582	Fisher	1·07 (0·32, 3·20)	0.911	Logistic	223	173	50
Fatigue n (%)	107 (62·9%)	30 (61·2%)	0.959	X²	0·89 (0·44, 1·83)	0.757	Logistic	219	170	49
FACIT fatigue score	36·3 (±11·8)	34·3 (±12·5)	0.317	T/Welch	-0·04 (-0·36, 0·27)	0.788	Norm linear	211	162	49
Anxiety (GAD- 7) Score	3·00 [0·00, 8·00]	2·00 [0·00, 8·00]	0.955	Mann-U	0·14 (-0·18, 0·46)	0.388	Norm linear	213	164	49
Anxiety (GAD-7 >8) n (%)	33 (20·1%)	10 (20·4%)	1.000	X²	1·52 (0·60, 3·71)	0.359	Logistic	213	164	49
Depression (PHQ-9) score	6·45 (±5·99)	7·92 (±7·78)	0.229	T/Welch	0·14 (-0·18, 0·47)	0.375	Norm linear	212	163	49
Depression (PHQ-9 >= 10) n (%)	47 (28·8%)	17 (34·7%)	0.545	X²	1·70 (0·78, 3·71)	0.179	Logistic	212	163	49
EQ-5D-5L utility	76·8 [67·9, 90·6]	73·6 [63·0, 100·0]	0.359	Mann-U	-0·08 (-0·41, 0·24)	0.623	Norm linear	213	166	47
EQ-5D-5L less than 60% n (%)	25 (15·1%)	9 (19·1%)	0.653	X²	1·44 (0·55, 3·56)	0.444	Logistic	213	166	47

Part C: Clinical characteristics of patients with and without renal abnormalities after excluding pre-existing renal conditions

Variable	No renal abnormalities (n= 184)	Renal abnormalities (n= 49)	P-value	Univariate test	Odds Ratio or Beta coefficient	p-value	Test	Total N	No-Abn N	Abn N
Age (years)	55.3 (±11.6)	60.0 (±13.7)	**0.029**	T/Welch	4.9 yrs [1.0, 8.7]*			233	184	49
Female n (%)	67 (36.4%)	26 (53.1%)						233	184	49
Body mass index (kg/m2)	30.9 (±5.4)	29.7 (±7.4)	0.290	T/Welch	-0.32 (-0.64, -0.00)	**0.049**	Linear	233	184	49
Obesity n (%)	97 (52.7%)	21 (42.9%)	0.286	X²	0.66 (0.34, 1.28)	0.224	Logistic	233	184	49
Systolic blood pressure (mmHg)	131.5 (±14.5)	136.3 (±16.0)	0.060	T/Welch	0.15 (-0.11, 0.41)	0.258	Linear	233	184	49
Kidney disease n (%)	0 (0.0%)	0 (0.0%)						231	182	49
Cardiac n (%)	20 (11.0%)	12 (24.5%)	**0.028**	X²	2.31 (0.89, 5.86)	0.079	Logistic	231	182	49

Hypertension n (%)	80 (44.4%)	30 (62.5%)	**0.039**	X²	1.91 (0.95, 3.92)	0.072	Logistic	228	180	48
Charlson index zero or one n (%)	163 (88.6%)	42 (85.7%)	0.622	Fisher				233	184	49
Charlson index of two or more n (%)	21 (11.4%)	7 (14.3%)								
WHO clinical progression scale n (%)										
WHO – class 3-4	33 (18.2%)	9 (18.8%)	1.000	Fisher				229	181	48
WHO – class 5	99 (54.7%)	27 (56.2%)								
WHO – class 6	36 (19.9%)	9 (18.8%)								
WHO – class 7-9	13 (7.2%)	3 (6.2%)								
Admission duration (days)	6.00 [3.00, 10.00]	6.0 [3.8, 9.2]	0.796	Mann-U	0.07 (-0.26, 0.41)	0.662	Linear	229	181	48
Acute biochemistry										
Acute cTnI Troponin	6.0 [2.0, 10.0]	179.0 [179.0, 179.0]			3.25 (0.60, 5.90)	**0.022**	Linear	18	17	1
Acute kidney injury n (%)	21 (11.6%)	10 (20.8%)	0.154	X²	1.58 (0.58, 4.09)	0.352	Logistic	229	181	48
Acute eGFR (ml/min per 1.73m2)	86.0 [71.0, 90.0]	80.0 [63.0, 90.0]	0.090	Mann-U	-0.12 (-0.42, 0.19)	0.449	Linear	225	179	46
Acute eGFR < 60 ml/min per 1.73m2 n (%)	20 (11.2%)	10 (21.7%)	0.102	X²	1.74 (0.63, 4.60)	0.273	Logistic	225	179	46
Treatments										
Remdesivir n (%)	20 (23.8%)	5 (26.3%)	0.775	Fisher	1.19 (0.33, 3.88)	0.781	Logistic	103	84	19
Systemic (oral or IV) steroids n (%)	139 (77.2%)	33 (68.8%)	0.306	X²	0.59 (0.28, 1.27)	0.171	Logistic	228	180	48
Therapeutic dose anti-coagulation n (%)	92 (50.8%)	23 (47.9%)	0.844	X²	0.70 (0.35, 1.40)	0.316	Logistic	229	181	48
Non-steroidal anti-inflammatory (NSAID)	13 (7.2%)	9 (18.8%)	**0.025**	Fisher	3.24 (1.17, 8.78)	**0.021**	Logistic	229	181	48
Follow-up investigations										
Ratio of FEV1 and FVC (L)	0.80 (±0.09)	0.74 (±0.10)	**0.006**	T/Welch	-0.42 (-0.78, -0.06)	**0.023**	Linear	158	121	37
TLCO mmol/min/kPa (SI)	8.27 (±1.91)	6.74 (±2.13)	**0.006**	T/Welch	-0.49 (-0.97, -0.01)	**0.045**	Linear	79	57	22
Follow-up eGFR (ml/min per 1.73m2) n (%)	90.0 [80.0, 90.0]	87.0 [71.0, 90.0]	0.082	Mann-U	-0.21 (-0.54, 0.12)	0.215	Linear	198	157	41
Follow-up eGFR < 60 (ml/min per 1.73m2) n (%)	3 (1.9%)	4 (9.8%)	**0.035**	Fisher	2.50 (0.38, 16.95)	0.330	Logistic	198	157	41
Albumin: creatinine ratio (ACR) Result (mg/mmol)	1.15 [0.70, 2.03]	1.25 [0.88, 2.33]	0.438	Mann-U	0.15 (-0.41, 0.71)	0.590	Linear	94	78	16
ACR > 10 mg/mmol (%)	3 (3.8%)	2 (12.5%)	0.200	Fisher				94	78	16
Serum creatinine (umol/L)	76.0 (±14.0)	73.1 (±21.8)	0.417	T/Welch	-0.15 (-0.47, 0.16)	0.332	Linear	201	160	41
Serum creatinine (>104 for men, >84 for women) n (%)	10 (6.2%)	7 (17.1%)	0.052	Fisher	2.49 (0.79, 7.48)	0.108	Logistic	201	160	41
Follow-up Pro-NT BNP pg/ml	43.0 [35.0, 77.0]	74.5 [36.5, 143.5]	**0.012**	Mann-U	0.42 (0.03, 0.81)	**0.033**	Linear	151	121	30
Follow-up any BNP > normal n (%)	18 (14.1%)	10 (31.2%)	**0.043**	X²	2.60 (0.91, 7.25)	0.068	Logistic	160	128	32
Haemoglobin - Result (g/L)	146.0 [137.0, 156.0]	135.0 [130.0, 145.0]	**<0.001**	Mann-U	-0.36 (-0.66, -0.05)	**0.021**	Linear	200	159	41
Platelets - Result (10^9/L)	253.4 (±65.2)	262.3 (±94.4)	0.573	T/Welch	-0.01 (-0.37, 0.35)	0.954	Linear	200	159	41
Platelets > 400 10^9/L	5 (3.1%)	3 (7.3%)	0.365	Fisher	3.30 (0.58, 17.21)	0.152	Logistic	200	159	41
White blood cell count (10^9/L)	6.60 [5.55, 7.90]	7.20 [6.10, 8.40]	0.245	Mann-U	0.15 (-0.22, 0.53)	0.415	Linear	200	159	41
WBC count < 4 (10^9/L) n (%)	3 (1.9%)	3 (7.3%)	0.102	Fisher	7.59 (1.15, 57.92)	**0.034**	Logistic	200	159	41
Do you feel recovered? n (%)										
No	83 (49.4%)	23 (53.5%)	0.712	Fisher				211	168	43
Not sure	35 (20.8%)	10 (23.3%)								
Yes	50 (29.8%)	10 (23.3%)								
Reported outcomes										
Abdominal pain n (%)	32 (18.9%)	12 (27.9%)	0.278	X²	1.75 (0.74, 3.98)	0.189	Logistic	212	169	43
Nausea/vomiting n (%)	13 (7.7%)	2 (4.7%)	0.741	Fisher	0.60 (0.09, 2.48)	0.526	Logistic	212	169	43
Loss of control of passing urine n (%)	15 (8.9%)	4 (9.3%)	1.000	Fisher	0.75 (0.18, 2.50)	0.655	Logistic	212	169	43
Fatigue n (%)	105 (63.3%)	27 (64.3%)	1.000	X²	0.99 (0.46, 2.16)	0.978	Logistic	208	166	42
FACIT fatigue score	36.2 (±11.9)	33.6 (±12.1)	0.213	T/Welch	-0.04 (-0.37, 0.30)	0.833	Linear	201	158	43
Anxiety (GAD- 7) Score	3.00 [0.00, 8.00]	2.00 [0.00, 7.50]	0.874	Mann-U	0.07 (-0.27, 0.41)	0.686	Linear	203	160	43
Anxiety (GAD-7 >8) n (%)	33 (20.6%)	8 (18.6%)	0.937	X²	1.17 (0.42, 3.03)	0.754	Logistic	203	160	43
Depression (PHQ-9) score	6.46 (±6.04)	7.70 (±7.40)	0.316	T/Welch	0.05 (-0.29, 0.38)	0.792	Linear	202	159	43
Depression (PHQ-9 >= 10) n (%)	46 (28.9%)	15 (34.9%)	0.571	X²	1.58 (0.69, 3.60)	0.271	Logistic	202	159	43
EQ-5D-5L utility	76.8 [67.9, 90.6]	73.3 [64.3, 100.0]	0.542	Mann-U	0.01 (-0.33, 0.35)	0.945	Linear	203	161	42
EQ-5D-5L less than 60% n (%)	25 (15.5%)	7 (16.7%)	1.000	X²	1.05 (0.36, 2.83)	0.918	Logistic	203	161	42

Abn N number of cases with abnormal renal MRI, BMI body mass index, BNP B-type natriuretic peptide, C-reactive protein, EQ-5D-5L quality of life, FACIT Functional Assessment of Chronic Illness Therapy, GFR glomerular filtration rate, IV intravenous, KCO carbon monoxide transfer co-efficient, FEV1 forced expiratory volume in 1 second, FVC forced vital capacity, No-Abn N number of cases without abnormal renal MRI, NT N-terminal, cT1nI troponin I, MOCA montreal cognitive assessment, GAD-7 generalised anxiety disorder, PHQ-9 personal health questionnaire, SQR square, TLCO transfer capacity of lung, ULN upper limit of normal. For Part A, inverse probability weighting was used to adjust imaging variable for confounders which included age, sex, body mass index, smoking, hypertension, hypercholesterolemia, diabetes, cardiac, brain, liver, lung and renal comorbidities, and scanner manufacturer. Part B and Part C (without corresponding organ comorbidities) regression models were adjusted for following covariates: age, sex, smoking, hypertension, diabetes, Charlson comorbidity index, obesity and scanner manufacturer. *Confidence intervals for age difference are not adjusted.

Supplementary Table 7. Post-hoc comparison of clinical characteristics and MRI organ abnormalities between post-hospitalised COVID-19 patients and controls matched for age, sex and obesity.

Variables		Controls	Cases	Sample size (all)	Missing data n (%)	Control (n)	Cases (n)	P-value	Standardised β or OR	P-value	Regression
Age (years)		49.3 (±13.9)	53.5 (±10.7)	259	0 (0.0%)	52	207	0.064			
Sex	Female	22 (42.3%)	85 (41.1%)	259	0 (0.0%)	52	207				
Non-white ethnicity	n(%)	14 (26.9%)	57 (27.5%)	259	0 (0.0%)	52	207	1.000			
Obesity (BMI>30kg/m2)	n(%)	19 (36.5%)	102 (49.3%)	259	0 (0.0%)	52	207	0.136			
Systolic Blood pressure (mm of Hg)		134.9 (±21.2)	131.3 (±15.2)	259	0 (0.0%)	52	207	0.247			
Smoker - non	0	43 (82.7%)	139 (67.1%)	259	0 (0.0%)	52	207	0.051			
ex-smoker	1	6 (11.5%)	56 (27.1%)								
Current smoker	2	3 (5.8%)	12 (5.8%)								
Alcohol intake (units/week)		1.00 [0.00, 1.00]	1.00 [0.00, 6.00]	233	26(10%)	52	181	**0.042**			
Index of multiple deprivation	1 - most deprived		37 (18.0%)	205	54 (20.8%)	0	205				
	2		40 (19.5%)								
	3		34 (16.6%)								
	4		50 (24.4%)								
	5 - least deprived		44 (21.5%)								
Respiratory comorbidity	n(%)	8 (15.4%)	60 (29.1%)	258	1 (0.4%)	52	206	0.067	2.29 (1.03, 5.66)	0.055	Logistic
Autoimmune disease	n(%)	0 (0.0%)	28 (13.6%)	258	1 (0.4%)	52	206	**0.010**		0.990	Logistic
Cardiac comorbidity	n(%)	2 (3.8%)	20 (9.7%)	258	1 (0.4%)	52	206	0.266	2.36 (0.61, 15.58)	0.273	Logistic
Diabetes	n(%)	7 (13.5%)	38 (18.4%)	258	1 (0.4%)	52	206	0.521	0.98 (0.37, 2.87)	0.976	Logistic
Gastrointestinal comorbidity	n(%)	NA (NA%)	17 (8.3%)	206	53 (20.5%)	0	206				
Hypercholestrolemia	n(%)	7 (13.5%)	29 (14.1%)	258	1 (0.4%)	52	206	1.000	0.69 (0.26, 2.01)	0.481	Logistic
Hypertension	n(%)	15 (28.8%)	90 (44.1%)	256	3 (1.2%)	52	204	0.066	1.69 (0.83, 3.55)	0.155	Logistic
Renal comorbidity	n(%)	1 (1.9%)	9 (4.4%)	258	1 (0.4%)	52	206	0.692	1.27 (0.18, 25.56)	0.836	Logistic
Liver comorbidity	n(%)	0 (0.0%)	13 (6.3%)	258	1 (0.4%)	52	206	0.077		0.994	Logistic
Neurological comorbidity	n(%)	1 (1.9%)	6 (2.9%)	258	1 (0.4%)	52	206	1.000	0.79 (0.08, 17.30)	0.851	Logistic
Anxiety, depression or other mental diagnosis	n(%)	NA (NA%)	32 (15.5%)	206	53 (20.5%)	0	206				
Charlson comorbidity index		0.00 [0.00, 0.00]	0.00 [0.00, 1.00]	258	1 (0.4%)	52	206	**0.042**	0.28 (-0.16, 0.73)	0.212	Normalised linear
Charlson comorbdiity category	0	45 (86.5%)	183 (88.4%)	259	0 (0.0%)	52	207	0.811			
	1	7 (13.5%)	24 (11.6%)								
WHO clinical progression scale		52 (100.0%)		257	2 (0.8%)	52	205	**<0.001**			
	WHO – class 3-4		39 (19.0%)								
	WHO – class 5		107 (52.2%)								
	WHO – class 6		43 (21.0%)								
	WHO – class 7-9		16 (7.8%)								
Multiorgan MRI abnormality count	0	12 (23.1%)	30 (14.5%)	259	0 (0.0%)	52	207	**<0.001**			
	1	26 (50.0%)	61 (29.5%)								
	2 0r more	14 (26.9%)	116 (56.0%)								
Lung MRI abnormalities (2 or more organs)	n(%)	3 (6.1%)	76 (36.7%)	256	3 (1.2%)	49	207	**<0.001**	9.84 (3.25, 43.02)	**<0.001**	Logistic
Brain MRI abnormalities	n(%)	9 (18.0%)	76 (43.2%)	226	33 (12.7%)	50	176	**0.002**	2.47 (1.08, 6.13)	**<0.001**	Logistic
Cardiac MRI abnormalities	n(%)	12 (24.5%)	36 (17.4%)	256	3 (1.2%)	49	207	0.347	0.56 (0.25, 1.28)	0.157	Logistic

Kidney MRI abnormalities	n(%)	3 (6.2%)	38 (19.2%)	246	13 (5.0%)	48	198	0.052	2.86 (0.92, 12.63)	0.105	Logistic
Liver MRI abnormalities	n(%)	28 (58.3%)	115 (60.8%)	237	22 (8.5%)	48	189	0.879	0.71 (0.33, 1.48)	0.369	Logistic

BMI body mass index, WHO world health organisation, MRI Magnetic Resonance Imaging.

References

1. Evans RA, McAuley H, Harrison EM, et al. Physical, cognitive, and mental health impacts of COVID-19 after hospitalisation (PHOSP-COVID): a UK multicentre, prospective cohort study. *Lancet Respir Med* 2021; **9**(11): 1275-87.
2. A minimal common outcome measure set for COVID-19 clinical research. *Lancet Infect Dis* 2020; **20**(8): e192-e7.
3. World Health Organisation. Clinical management of COVID-19 Interim Guidance. Accessed on Aug 2, 2020. https://www.who.int/publications/i/item/clinical-management-of-covid-19.
4. World Health Organisation. COVID-19 Therapeutic Trial Synopsis Accessed Aug 2, 2020. https://www.who.int/blueprint/priority-diseases/key-action/COVID-19_Treatment_Trial_Design_Master_Protocol_synopsis_Final_18022020.pdf (accessed August 2 2020).
5. Avants BB, Tustison NJ, Song G, Cook PA, Klein A, Gee JC. A reproducible evaluation of ANTs similarity metric performance in brain image registration. *Neuroimage* 2011; **54**(3): 2033-44.
6. Lin YR, Tsai SY, Huang TY, et al. Inflow-weighted pulmonary perfusion: comparison between dynamic contrast-enhanced MRI versus perfusion scintigraphy in complex pulmonary circulation. *J Cardiovasc Magn Reson* 2013; **15**(1): 21.
7. Piechnik SK, Ferreira VM, Dall'Armellina E, et al. Shortened modified look-locker inversion recovery (ShMOLLI) for clinical myocardial T1-mapping at 1.5 And 3 T within a 9 heartbeat breathhold. *J Cardiovasc Magn Reson* 2010; **12**.
8. Carapella V, Puchta H, Lukaschuk E, et al. Standardized image post-processing of cardiovascular magnetic resonance T1-mapping reduces variability and improves accuracy and consistency in myocardial tissue characterization. *International Journal of Cardiology* 2020; **298**: 128-34.
9. Karamitsos TD, Hudsmith LE, Selvanayagam JB, Neubauer S, Francis JM. Operator induced variability in left ventricular measurements with cardiovascular magnetic resonance is improved after training. *Journal of Cardiovascular Magnetic Resonance* 2007; **9**(5): 777-83.
10. Schulz-Menger J, Bluemke DA, Bremerich J, et al. Standardized image interpretation and post processing in cardiovascular magnetic resonance: Society for Cardiovascular Magnetic Resonance (SCMR) board of trustees task force on standardized post processing. *J Cardiovasc Magn Reson* 2013; **15**: 35.
11. Dass S, Cochlin LE, Suttie JJ, et al. Exacerbation of cardiac energetic impairment during exercise in hypertrophic cardiomyopathy: a potential mechanism for diastolic dysfunction. *Eur Heart J* 2015; **36**(24): 1547-54.
12. Huang L, Zhao P, Tang D, et al. Cardiac Involvement in Patients Recovered From COVID-2019 Identified Using Magnetic Resonance Imaging. *JACC: Cardiovascular Imaging* 2020.
13. Piechnik SK, Jerosch-Herold M. Myocardial T1 mapping and extracellular volume quantification: an overview of technical and biological confounders. *Int J Cardiovasc Imaging* 2018; **34**(1): 3-14.
14. Vidalakis E, Kolentinis M, Gawor M, Vasquez M, Nagel E. CMR in Pericardial Diseases-an Update. *Current Cardiovascular Imaging Reports* 2020; **13**(4): 1-9.
15. Messroghli DR, Moon JC, Ferreira VM, et al. Clinical recommendations for cardiovascular magnetic resonance mapping of T1, T2, T2* and extracellular volume: A consensus statement by the Society for Cardiovascular Magnetic Resonance (SCMR) endorsed by the European Association for Cardiovascular Imaging (EACVI). *J Cardiovasc Magn Reson* 2017; **19**(1): 75.
16. Zhang Q, Werys K, Popescu IA, et al. Quality assurance of quantitative cardiac T1-mapping in multicenter clinical trials - A T1 phantom program from the hypertrophic cardiomyopathy registry (HCMR) study. *Int J Cardiol* 2021; **330**: 251-8.
17. Popescu IA, Werys K, Zhang Q, et al. Standardization of T1-mapping in cardiovascular magnetic resonance using clustered structuring for benchmarking normal ranges. *Int J Cardiol* 2021; **326**: 220-5.
18. Alfaro-Almagro F, Jenkinson M, Bangerter NK, et al. Image processing and Quality Control for the first 10,000 brain imaging datasets from UK Biobank. *Neuroimage* 2018; **166**: 400-24.
19. Alfaro-Almagro F, McCarthy P, Afyouni S, et al. Confound modelling in UK Biobank brain imaging. *NeuroImage* 2020: 117002.
20. Miller KL, Alfaro-Almagro F, Bangerter NK, et al. Multimodal population brain imaging in the UK Biobank prospective epidemiological study. *Nature neuroscience* 2016; **19**(11): 1523-36.
21. Alfaro-Almagro F, Jenkinson M, Bangerter NK, et al. Image processing and Quality Control for the first 10,000 brain imaging datasets from UK Biobank. *Neuroimage* 2018; **166**: 400-24.
22. Jenkinson M, Beckmann CF, Behrens TE, Woolrich MW, Smith SM. Fsl. *Neuroimage* 2012; **62**(2): 782-90.

23. Dale AM, Fischl B, Sereno MI. Cortical surface-based analysis. I. Segmentation and surface reconstruction. *Neuroimage* 1999; **9**(2): 179-94.
24. Lange FJ, Ashburner J, Smith SM, Andersson JLR. A Symmetric Prior for the Regularisation of Elastic Deformations: Improved anatomical plausibility in nonlinear image registration. *Neuroimage* 2020; **219**: 116962.
25. Andersson JLR, Jenkinson M, Smith S. High resolution nonlinear registration with simultaneous modelling of intensities. *bioRxiv* 2019: 646802.
26. Zhang Y, Brady M, Smith S. Segmentation of brain MR images through a hidden Markov random field model and the expectation-maximization algorithm. *IEEE transactions on medical imaging* 2001; **20**(1): 45-57.
27. Patenaude B, Smith SM, Kennedy DN, Jenkinson M. A Bayesian model of shape and appearance for subcortical brain segmentation. *Neuroimage* 2011; **56**(3): 907-22.
28. Griffanti L, Zamboni G, Khan A, et al. BIANCA (Brain Intensity AbNormality Classification Algorithm): A new tool for automated segmentation of white matter hyperintensities. *Neuroimage* 2016; **141**: 191-205.
29. DeCarli C, Fletcher E, Ramey V, Harvey D, Jagust WJ. Anatomical mapping of white matter hyperintensities (wmh) exploring the relationships between periventricular WMH, deep WMH, and total WMH burden. *Stroke* 2005; **36**(1): 50-5.
30. Griffanti L, Jenkinson M, Suri S, et al. Classification and characterization of periventricular and deep white matter hyperintensities on MRI: a study in older adults. *Neuroimage* 2018; **170**: 174-81.
31. Andersson A, Kelly M, Imajo K, et al. Clinical Utility of Magnetic Resonance Imaging Biomarkers for Identifying Nonalcoholic Steatohepatitis Patients at High Risk of Progression: A Multicenter Pooled Data and Meta-Analysis. *Clin Gastroenterol Hepatol* 2022; **20**(11): 2451-61.e3.
32. Banerjee R, Pavlides M, Tunnicliffe EM, et al. Multiparametric magnetic resonance for the non-invasive diagnosis of liver disease. *J Hepatol* 2014; **60**(1): 69-77.
33. Triay Bagur A, Hutton C, Irving B, Gyngell ML, Robson MD, Brady M. Magnitude-intrinsic water-fat ambiguity can be resolved with multipeak fat modeling and a multipoint search method. *Magn Reson Med* 2019; **82**(1): 460-75.
34. Hernando D, Zhao R, Yuan Q, et al. Multicenter Reproducibility of Liver Iron Quantification with 1.5-T and 3.0-T MRI. *Radiology*; **0**(0): 213256.
35. Tunnicliffe EM, Banerjee R, Pavlides M, Neubauer S, Robson MD. A model for hepatic fibrosis: the competing effects of cell loss and iron on shortened modified Look-Locker inversion recovery T(1) (shMOLLI-T(1)) in the liver. *J Magn Reson Imaging* 2017; **45**(2): 450-62.
36. Bachtiar V, Kelly MD, Wilman HR, et al. Repeatability and reproducibility of multiparametric magnetic resonance imaging of the liver. *PLoS One* 2019; **14**(4): e0214921.
37. Harrison SA, Rossi SJ, Paredes AH, et al. NGM282 Improves Liver Fibrosis and Histology in 12 Weeks in Patients With Nonalcoholic Steatohepatitis. *Hepatology* 2020; **71**(4): 1198-212.
38. Harrison SA, Gawrieh S, Roberts K, et al. Prospective evaluation of the prevalence of non-alcoholic fatty liver disease and steatohepatitis in a large middle-aged US cohort. *J Hepatol* 2021; **75**(2): 284-91.
39. Mojtahed A, Kelly CJ, Herlihy AH, et al. Reference range of liver corrected T1 values in a population at low risk for fatty liver disease-a UK Biobank sub-study, with an appendix of interesting cases. *Abdom Radiol (NY)* 2019; **44**(1): 72-84.
40. Cox EF, Buchanan CE, Bradley CR, et al. Multiparametric renal magnetic resonance imaging: validation, interventions, and alterations in chronic kidney disease. *Frontiers in physiology* 2017; **8**: 696.
41. Bane O, Mendichovszky IA, Milani B, et al. Consensus-based technical recommendations for clinical translation of renal BOLD MRI. *Magnetic Resonance Materials in Physics, Biology and Medicine* 2020: 1-17.
42. Daniel AJ, Buchanan CE, Allcock T, et al. Automated renal segmentation in healthy and chronic kidney disease subjects using a convolutional neural network. *Magnetic Resonance in Medicine* 2021; **86**(2): 1125-36.
43. Cox EF, Buchanan CE, Bradley CR, et al. Multiparametric Renal Magnetic Resonance Imaging: Validation, Interventions, and Alterations in Chronic Kidney Disease. *Front Physiol* 2017; **8**: 696.
44. Miller MR, Hankinson J, Brusasco V, et al. Standardisation of spirometry. *European respiratory journal* 2005; **26**(2): 319-38.
45. Kroenke K, Spitzer RL, Williams JB. The PHQ-9: validity of a brief depression severity measure. *Journal of general internal medicine* 2001; **16**(9): 606-13.
46. Spitzer RL, Kroenke K, Williams JB, Löwe B. A brief measure for assessing generalized anxiety disorder: the GAD-7. *Archives of internal medicine* 2006; **166**(10): 1092-7.
47. Nasreddine ZS, Phillips NA, Bédirian V, et al. The Montreal Cognitive Assessment, MoCA: a brief screening tool for mild cognitive impairment. *Journal of the American Geriatrics Society* 2005; **53**(4): 695-9.

48. Yorke J, Moosavi SH, Shuldham C, Jones PW. Quantification of dyspnoea using descriptors: development and initial testing of the Dyspnoea-12. *Thorax* 2010; **65**(1): 21-6.
49. Butt Z, Lai J-s, Rao D, Heinemann AW, Bill A, Cella D. Measurement of fatigue in cancer, stroke, and HIV using the functional assessment of chronic illness therapy—fatigue (FACIT-F) scale. *Journal of psychosomatic research* 2013; **74**(1): 64-8.
50. Weathers FW, Litz BT, Keane TM, Palmieri PA, Marx BP, Schnurr PP. The ptsd checklist for dsm-5 (pcl-5). 2013.
